# Screening fructosamine-3-kinase (FN3K) inhibitors, a deglycating enzyme of oncogenic Nrf2: Human FN3K homology modelling, docking and molecular dynamics simulations

**DOI:** 10.1371/journal.pone.0283705

**Published:** 2023-11-01

**Authors:** Narasimha M. Beeraka, Jin Zhang, Subhankar Mandal, Hemanth Vikram P. R., Junqi Liu, Namitha B. M., Di Zhao, Prashanth Vishwanath, Gurupadayya B. M., Ruitai Fan

**Affiliations:** 1 Cancer Center, The First Affiliated Hospital of Zhengzhou University, Zhengzhou, China; 2 Department of Pharmaceutical Chemistry, JSS College of Pharmacy, JSS Academy of Higher Education & Research (JSS AHER), Mysuru, Karnataka, India; 3 Department of Human Anatomy, I.M. Sechenov First Moscow State Medical University (Sechenov University), Moscow, Russian Federation; 4 Raghavendra Institute of Pharmaceutical Education and Research (RIPER), Anantapuramu, Chiyyedu, Andhra Pradesh, India; 5 Herman B. Wells Center for Pediatric Research, Department of Pediatrics, Indiana University School of Medicine, Indianapolis, IN, United States of America; 6 Department of Pharmacology and Toxicology, University of Mississippi Medical Center, Jackson, Mississippi, United States of America; 7 Department of Radiation Oncology, The First Affiliated Hospital of Zhengzhou University, Zhengzhou, China; 8 Department of Pharmacology, JSS College of Pharmacy, JSS Academy of Higher Education & Research (JSS AHER), Mysuru, Karnataka, India; 9 Department of Endocrinology, The First Affiliated Hospital of Zhengzhou University, Zhengzhou, China; 10 Department of Biochemistry, Center of Excellence in Molecular Biology and Regenerative Medicine, JSS Medical College, JSS Academy of Higher Education and Research, Mysore, India; Gauhati University, INDIA

## Abstract

Fructosamine-3-kinase (FN3K) is involved in the deglycation of Nrf2, a significant regulator of oxidative stress in cancer cells. However, the intricate functional aspects of FN3K and Nrf2 in breast cancers have not been explored vividly. The objectives of this study are to design the human FN3K protein using homology modeling followed by the screening of several anticancer molecules and examining their efficacy to modulate FN3K activity, Nrf2-mediated antioxidant signalling. Methods pertinent to homology modeling, virtual screening, molecular docking, molecular dynamics simulations, assessment of ADME properties, cytotoxicity assays for anticancer molecules of natural/synthetic origin in breast cancer cells (BT-474, T-47D), and Western blotting were used in this study. The screened anticancer molecules including kinase inhibitors of natural and synthetic origin interacted with the 3-dimensional structure of the catalytic domain in human FN3K protein designed through homology modeling by significant CDOCKER interaction energies. Subsequently, gefitinib, sorafenib, neratinib, tamoxifen citrate, and cyclosporine A enhanced the expression of FN3K in BT-474 cell lines with simultaneous alteration in Nrf2-driven antioxidant signalling. Oxaliplatin significantly downregulated FN3K expression and modulated Nrf2-driven antioxidant signalling when compared to cisplatin and other anticancer drugs. Hence, the study concluded the potential implications of existing anticancer drugs to modulate FN3K activity in breast cancers.

## Introduction

Obesity, inheritance, lifestyle patterns, and pollution can cause the risk of breast cancer incidence as age increases in women [1]. In addition, severe tobacco usage, and postmenopausal hormone therapy can be other risk factors to induce breast cancer. Mortality depending on age-standardized breast cancer incidence is enhanced in recent years [1]. However, the lack of effective timely prognostic strategies leads to late detection or diagnosis and poor clinical outcomes due to several tumor-wise, node-wise, and metastasis-wise invasions. Additionally, the stemness-inducing factors across the tumor microenvironment are another hindrance to the overall survival in breast cancer patients [2]. Therefore, efficient therapeutic strategies are required to inhibit breast tumor growth and metastasis. Since existing therapies such as surgery and chemoradiotherapy are significant therapeutic modalities but the recurrence rate of breast cancer is still higher. Hence, novel combination therapies that specifically deliver drugs with sustained release properties are urgently needed to treat breast cancers. Current therapies for treating metastatic advanced stages of breast cancers are either less effective or target only a set of population [3]. In addition, the development of drug-resistant phenotypes worsens this situation as the majority of existing treatments are found to be ineffective against these cancers. Albeit several chemotherapies have been preferred to target breast cancer, the cancer cells in the metastatic stage have been acquiring chemoresistance due to stemness. Henceforth typically a higher requirement of chemotherapy dosage regimen is preferred to the breast cancer patients which recurrently induce dose-related toxicity and early mortality.

Activation of Nrf2 is higher in the dividing cancer cells due to mutations in the majority of tumors such as liver, lung, head, and neck [4], bladder, and pancreas [4]. The Nrf2 could be considered the crucial oncogene involved in driving oncogenesis [5]. Previous studies delineated that the less-frequent somatic mutations, which could affect Nrf2 glycation sites including R499W, R569C, and R569H in major cancers such as endometrial, colorectal carcinoma, and melanomas. FN3K is reported to be involved in deglycating the Nrf2 through phosphorylation of lysine, and arginine and enhancing Nrf2 activity in hepatocellular carcinoma, consequently protecting the cancer cells through antioxidant mechanisms [4]. Therefore, the development of small molecule inhibitors is a significant approach to selectively block FN3K in cancers. Previous study described the profound efficacy of Nrf2 inhibitors for fostering the retardation of breast cancer [6]. FN3K is a deglycating enzyme reported to induce deglycation of Nrf2 which results in the activation of antioxidant responses to regulate oxidative stress to offer cancer cell protection [4]. Deglycated Nrf2 predominantly induces antioxidant responses by binding to sMAF. It may be a highly significant targeting molecule to promote the development of therapeutic molecules of either synthetic or natural origin to control the activity of FN3K, subsequently to confer Nrf2 in the glycated state in cancer cells including breast cancers.

Rational drug design through virtual screening, molecular docking, and molecular dynamics could enhance the rapid screening process of existing anticancer molecules to modulate the several oncogenic proteins involved in oncogenesis [4, 7]. Thus, it is possible to develop more efficient drug candidates. For instance, the kinases can have a prominent function in modulating the oncogenesis in many cancers and several kinase modulators could be implicated as antineoplastic agents for the retardation of cancers [8]. Furthermore, the phytochemicals like brusatol, topotecan, and platinum derivatives such as cisplatin, and oxaliplatin could be used as combinatorial regimens along with kinase modulators, which require substantial studies. In this study, we have designed the human FN3K three-dimensional protein through homology modeling and subsequently screened several anticancer kinase inhibitors of synthetic and natural origin and analyzed the modulating efficacy of platinum derivatives, and other anticancer drugs onto FN3K protein expression patterns using in vitro models. The overall workflow of the study was depicted in the following diagram.

## Methods

### Ethical statement

This study does not involve animals or human subjects. Therefore, ethical permission is not required.

### Chemicals and reagents

DMEM with high glucose (4.5 g/L) (cat#: AL111), trypsin-EDTA (0.25%) (cat#: T001) were procured from HiMedia Laboratories Pvt. Ltd. (Bengaluru, Karnataka, India). DMSO pertinent to cell culture (cat#: D2650), radioimmunoprecipitation assay buffer (cat#: R0278), protease inhibitor cocktail (cat#: S8820), bovine serum albumin (BSA) (cat#: 05479), and cisplatin (cat#:1134357), oxaliplatin (CAS: 61825-94-3), brusatol (cat#:SML 1868) were procured from Sigma Chemical Company (St. Louis, MO, USA). **Primary antibodies** for FN3K were procured (Catalog # PA5-28603) from Invitrogen whereas the primary antibodies labeling for Nrf2 (cat#: 12721, cell signalling), NQO1 (cat#: 62262), HO-1 (cat#: ab13248), Keap1 (cat#: ab227828) were procured from Abcam (Cambridge, MA, USA) and Cell Signalling Technologies (Danvers, MA, USA). Secondary antibodies (Rabbit cat#: SC2357 and Goat cat#: SC2020 were procured from Santa Cruz Biotechnology (Santa Cruz, CA, USA). FBS (cat#:10270106), Pen-Strep (cat#:150763) were procured from ThermoFisher Scientific (Waltham, MA, USA) Dilutions of all the primary antibodies and secondary antibodies were carried out as per the dilution factors given by the specification of the manufacturer. **Kinase inhibitors** such as sorafenib, tamoxifen citrate, neratinib, gefitinib, and other anti-cancer drugs including cyclosporine A, methotrexate, topotecan, and brusatol were obtained from the Bi Biotech, New Delhi, India.

### 3D-protein homology sequence modeling

As there is no availability of three-dimensional structure of Human FN3K (*Homo sapiens*) available in either Uniprot or Protein Data Bank (PDB) databases, the 3-dimensional homology modeled FN3K was constructed using the available ‘specific template’ of FN3K (*Arabidopsis thaliana*, 6OID) using ‘SWISS EXPASY Tools’ (SIB) [9].

Primarily, the amino acid FASTA sequences (Uniprot, 309 AA, accession number: Q9H479), of FN3K in *Homo sapiens* and *A*. *thaliana* (6OID, 317AA (PDB)) were collected from NCBI (http://www.ncbi.nlm.nih.gov). Subsequently, the phylogenetic similarities in the sequences of these two were ascertained with the aid of Clustal W tools (https://www.ebi.ac.uk/Tools /msa/clustalo/). A template for homology structural modeling was developed with the help of the SWISS-Model (https://swissmodel.expasy.org/) [9]. Subsequently, these models were subjected to refining using Galaxy Web (http://galaxy.seoklab.org/index.html), and verified using Ramachandran plots, and PROCHECK (https://servicesn.mbi.ucla.edu/PROCHECK/) analysis, global model quality estimation (GMQE) score and qualitative model energy analysis (QMEAN) values. All these procedures were executed as per Bharadwaja Vadloori et al [10]. As a control, the 3D structure of FN3K reported from *A*. *thaliana* was considered to be a reference to validate the designed 3D homology model of human FN3K using Ramachandran plots and PROCHECK. During the modeling process, the crystallized ligand was placed in its original position to rebuild the binding cavity. The resulting human FN3K model was subjected to PROCHECK validation [11].

### Computer-aided drug design [Screening of anticancer molecules including protein kinase inhibitors for molecular docking]: Ligand preparation

For our study, we have selected anticancer molecules including synthetic protein kinase inhibitors, and natural protein kinase inhibitors to check their binding affinity with ATP binding loop of designed human FN3K 3D-homology sequence to optimize kinase inhibitors using CADD techniques. A report by Kiyoaki Yonesu et al (2011) reported that the lysates of red blood cells incubated with 1-deoxy-1-morpholinofructose (DMF), a competitive inhibitor of FN3K, could inhibit ∼10% of total FN3K activity [12]. Hence, the DMF structure file was obtained from Pubchem (CID, 220877) and subsequently copied to Chem 3D ultra 10.0 and generated a three-dimensional model; later, the model was subjected to energy minimization with the aid of molecular mechanics (MM2) until the root mean square gradient attained specific value lower than 0.001 Kcal/mol. These kinds of structures with energy minimization were generally required for efficient docking. MDL MOL files of these structures were generated and saved as /MDL MOL. The ligand preparation was executed as per the procedures described by Awwad Radwan et al 2020 [13].

### 3D homology model protein preparation

X-ray diffraction properties and resolution of the structure (2.0–2.5 Ǻ) as well as the presence of co-crystallized ligand are the most significant factors required to choose a three-dimensional protein preparation for an efficient docking process. However, we do not have the ‘existing 3D of FN3K of *Homo sapiens*’, henceforth designed using SWISS Expasy tools. Ramachandran plot statistics were considered as the crucial validation filter to perform protein selection in which none of the amino acid residues were in disallowed regions. Assignment of Kollmann charges was executed before docking. All the protein preparation procedures were executed according to the procedures given by Awwad Radwan et al 2020 [13].

### Molecular docking

Molecular docking was executed onto the monomer of FN3K using Discovery studio. Primarily, the blind docking was executed and subsequently, the docking within the specified search space around the probable binding site of FN3K was performed. Docking conformations were chosen as per the binding affinity. Pymol and discovery studio visualizer was used to visualize 3D graphical representations (https://www.pymol.org/). Natural kinase inhibitors and FDA-approved synthetic kinase inhibitors were collected from the literature reports and databases for molecular docking to dock onto the designed 3-dimensional FN3K protein. Preliminary screening of >100 anticancer molecules was executed through LibDock Program, [14] a screen-based docking procedure to screen the number of hits from the database. Based on the LibDock score, we identified a total of ‘40’ molecules as hits. Among them, the potential lead molecules were identified and docked using a simulated annealing-based CDocker approach [14].

Specific algorithms were used in the Discovery studio docker tool to examine the intermolecular interactions of numerous ligands with macromolecules like 3-dimensional proteins. CDOCKER was one of the significant algorithms applied in a simulated annealing-based method to allow structural parameterization in CHARMm force fields [15–17]. Later, any structural inappropriacy was eliminated by performing adequate and suitable corrections of loop regions or side chains, or conformations.

On the other hand, the ligands were also subjected to processing to fix valences and charges in CHARMm force field. Specific ATP binding region was identified by ‘searching for potential receptor cavity [binding site specifications: volume: 1098 Å^3^, point count: 8340 0.5(X), 0.5(Y), and 0.5(Z) directions-equal gird spacing]’ subsequently executed the docking procedures [sphere site: 23. 28 (X), 21.01 (Y), and 2.16 (Z)] by ligand hits onto active binding site cavities using docking algorithm.

Pose-resolved interactions pertinent to the docked ligands were collected for subsequent analysis and MD simulation [14]. All the molecular docking procedures were executed as described by Awwad Radwan et al 2020 [13].

### Molecular dynamics simulations [Gromacs 5.0]

Before MD simulations of 3D-modeled human FN3K, we initially performed the protein structure alignment of the homology FN3K model (cyan) with MEK1 PDB structure (yellow) (**Fig 2A**) to explore the binding and interaction efficacy; subsequently, the chosen ligands such as synthetic anticancer kinase inhibitors such as ‘neratinib (PubChem ID: 9915743), AZD1480 (PubChem ID: 16659841), Jadomycin B (PubChem ID: 164484), tamoxifen citrate (PubChem ID: 2733526), sorafenib (PubChem ID: 216239) ponatinib (PubChem ID: 24826799), gefitinib (PubChem ID: 123631), tricirbine (PubChem ID: 65399), and natural multikinase inhibitor, EGCG (PubChem ID: 65064)’, DMF (PubChem ID: 6454484), an FN3K inhibitor were subjected to the MD simulations in Gromacs 5.0 as per the procedures described by Vadloori et al 2018. Topological parameters pertinent to ligands were collected using the ATB server (https://atb.uq.edu.au/). Primarily, the protein-complex was placed in the cubix box having water with the aid of SPC/E water models. GROMOS54a7 force field was used to execute energy minimization and subjected to equilibration at 300K with the aid of V-rescale for the duration of 200 ps. NVT ensemble was used consequently and equilibration was performed at one atmospheric pressure with the aid of Parrinello-Rahman Algorithm. The NPT ensemble duration was 200 ps. The equilibrated conformation has been conducted for the duration of 20 nanoseconds simulation. Verlet was used for applying LINCS algorithm to perform bond constraints. Root mean square deviations (RMSD) and radius of gyration (Rg) at the time of simulation were calculated with the aid of gmx_rms tool procured through Gromacs 5.0. Primarily, all the hits from virtual screening were subjected to a 20 ns simulation run to estimate the binding efficiency, which successfully validated and adapted the molecular docking protocol. However, the confirmation of protein-ligand stability under a thermodynamically equilibrated environment was assessed by extending the production run for a 100 ns time frame of unbound protein and specific ligands (sorafenib and gefitinib) bound protein complexes. These ligands were associated with a significant binding affinity than the other molecules. The extended MD run was further employed to estimate the binding energies through MM-PBSA calculation [18–22].

### HPLC characterization

of sorafenib, tamoxifen citrate, neratinib, gefitinib, cisplatin, oxaliplatin, cyclosporine A, topotecan, brusatol at specific chromatography conditions was given as supplementary attachment (**S1 Table**).

### ADME

(absorption, distribution, metabolism, and excretion) properties of molecules screened (**Table 2**) deciphered using the computational SWISS ADMET Server. Descriptors with significant molecular properties pertinent to pharmaceutical compliance to Lipinski’s rule of five RO5 were calculated through the ‘SWISS ADMET Server’ as described by Ramesh Itteboina et al 2017 [23].

### Cytotoxicity assays [sulforhodamine B proliferation (SRB) assay]

BT-474 (luminal B) and T47D (luminal A) ductal invasive carcinomas type of breast cancer cells were cultured into 96 well plates (10000 cells/well) and subsequently subjected to incubation for 24 and 48 hours respectively. Later, the cells were treated with drugs for 24 hours and 48 hours respectively:

### BT-474/T47D

1. Neratinib: 10 μM, 5 μM, 2.5 μM, 1.25 μM, 0.625 μM, 0.312 μM, 0.156 μM
2. Sorafenib/tamoxifen citrate/topotecan: 1.5 μM, 3.125 μM, 6.25 μM, 12.5 μM, 25 μM, 50 μM, 100 μM
3. Cisplatin/oxaliplatin: 15.6 μM, 31.25 μM, 62. 5 μM, 125 μM, 250 μM, 500 μM, 1000 μM

### BT-474

1. Methotrexate: 7.8 μM, 15.6 μM, 31.25 μM, 62. 5 μM, 125 μM, 250 μM, 500 μM
2. Cyclosporine A: 3.9 μM, 7.8 μM, 15.6 μM, 31. 2 μM, 62. 5 μM, 125 μM, 250 μM
3. Brusatol: 20 μM, 10 μM, 5 μM, 2.5 μM, 1.25 μM, 0.625 μM, 0.312 μM

### T47D

1. Gefitinib: 7.8 μM, 15.6 μM, 31. 2 μM, 62. 5 μM, 125 μM, 250 μM, 500 μM
2. Cyclosporine A: 7.8 μM, 15.6 μM, 31. 2 μM, 62. 5 μM, 125 μM, 250 μM, 500 μM
3. Methotrexate: 15.6 μM, 31.25 μM, 62. 5 μM, 125 μM, 250 μM, 500 μM, 1000 μM
4. Brusatol: 20 μM, 10 μM, 5 μM, 2.5 μM, 1.25 μM, 0.625 μM, 0.312 μM

DMF (known FN3K inhibitor for BT-474 and T-47D: 0. 15 mM, 0.31 mM, 0.625 mM, 1.25 mM, 2.5 mM, 5 mM treated for 48 hours.

Vehicle control (DMSO) was maintained at <1%; cisplatin (100 μM) was used as a positive control; finally, each well in the plates was fixed using 100 μl cold 25% trichloracetic acid. Later, the plates were washed using distilled water and subjected to drying and, consequently, 50 μl SRB dye (0.4% SRB dissolved in 1% glacial acetic acid) was added to every well. Later, the plates were subjected to incubation for thirty minutes at 37°C. 1% glacial acetic acid was used for washing consequently the plates were dried for 3 hours. Later, every well in the plates was added 100 μl of Tris-NaOH buffer (pH 10.5, 10 mM) and subjected to incubation for one hour, and the reading was taken using Perkin Elmer multimode plate reader (UK) for ascertaining optical density at 540 nm. IC-50 calculations were performed with the aid of GraphPad Prism 7 package.

### Western blotting

Primarily, the cell lines such as MDA-MB-231, MDA-MB-468, MCF-7, BT-474, T-47D, A549, and Beas2B were screened for the expression patterns of FN3K, Nrf2 and Keap1. Later, the drug-treated lysates of BT-474 alone were analyzed for the FN3K, Nrf2, and their downstream antioxidant signaling. The total protein content of drug-treated BT-474 cells (sorafenib/tamoxifencitrate/neratininb/gefitnib/cisplatin/oxaliplatin/cyclosporine/methotrexate) was collected with the aid of lysis buffer subsequently protein content was determined through BCA method. Total of 50 μg of equal quantities were taken for SDS-PAGE running in 12% gel by applying 100 V for two hours. Later, the proteins were separated into PVDF/nitrocellulose membrane with the aid of semidry equipment. Skimmed milk of 5% was used to block the membranes for one hour in TBST buffer. Blots were subjected to TBST washing 3 times subsequently subjected to primary antibody loading and incubated overnight at 4°C. Later, the blots were washed using TBST thrice and subjected to incubation with ‘HRP-linked secondary antibody’ for two hours. ECL (1:1 ratio of reagent 1 and reagent 2) was added to the above samples and observed for banding pattern in Chemi-UV tech in the dark till the bands appeared. Additionally, FN3K expression alone was probed in T47D cell lines using Western blotting.

### NQO1 activity assay

This procedure was executed as per the protocol described by Bovilla et al (2021) [6]. NQO1 activity for the drug-treated lysates of BT-474 cells was examined with the aid of a standard reaction of glucose-6-phosphate (G6P) when incubated with G6P dehydrogenase (G6Pdase). In this reaction, NADPH was generated and it can induce the reduction of menadione into menadiol. Subsequently, the formazan in blue color was generated and ascertained at 610 nm with the aid of a multimode plate reader. Furthermore, dicoumarol has been used to examine the background activity induced through reductases.

The total protein content of 10 μg obtained from the drug-treated cell lines was diluted to 40 μL with Millipore water and subjected to incubation using NQO1 cocktail (200 μL) in the presence/absence of dicoumarol [3 samples with inhibitor whereas 3 samples without dicoumarol; 6 wells/test]. Subsequently, absorbance was obtained at 610 nm for 60 minutes with one minute interval. The NQO1 activity was determined by performing subtraction of readings pertinent to ‘samples with inhibitor’ from readings pertinent to ‘samples without inhibitor’. OD values were deciphered per minute whereas the mole units were determined by multiplying the OD/minute/molar extinction coefficient of MTT with the protein concentration of each sample. Finally, the NQO1 activity was represented in ‘specific activity (μmol/min/mg protein)’.

### Statistics

Experiments were repeated in triplicates and the data was indicated in Mean ± SEM. The level of significance was calculated with the aid of GraphPad Prism version 6.0 (Graphpad Software, CA, USA). Other statistical tests such as one-way ANOVA and Tukey’s post hoc test were executed to compare different experimental groups. The “p” values <0.05, <0.01, <0.001 were considered statistically significant.

## Results

### Sequence alignment and homology modeling

**Fig 1:**
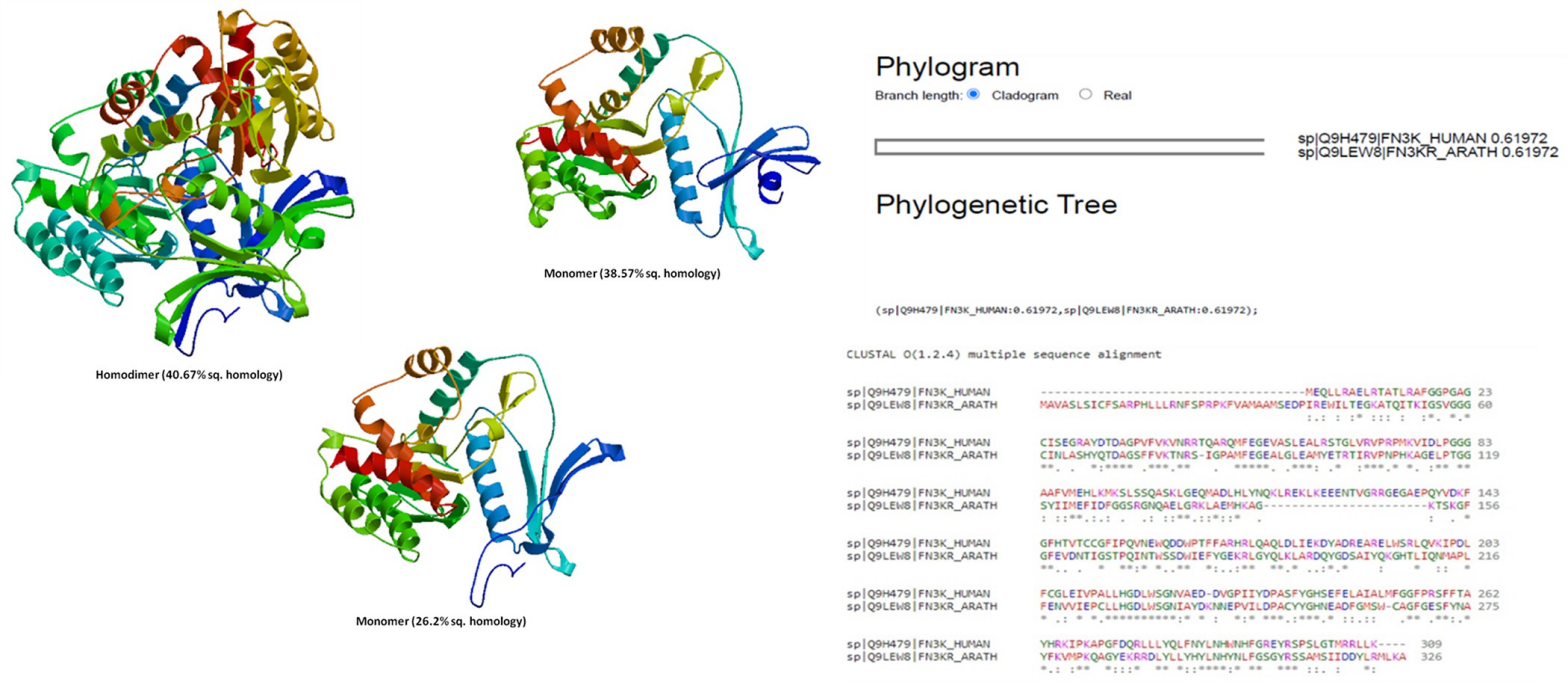
**A**. Human FN3K homology modeling was performed using Swiss-Model EXPASY tools and 3 reliable models were obtained including 1 ‘homodimer’, and 2 monomers. Homodimer with 40.67% of sequence similarity was further considered for molecular docking. **B**. Phylogenetic similarity was depicted by cladogram of human FN3K with FN3K of *Arabidopsis thaliana* (AtFN3K: PDB-ID: 6OID) and the multiple sequence alignment between Human FN3K and AtFN3K [9, 13].

According to a previous study by Roberto Sánchez et al (1999), if two proteins exhibit 30% of “segment identity,” then Cα atoms pertinent to these proteins are within 3.5 Å [24]. For instance, AtFN3K (*Arabdosis thaliana* FN3K) adopts a canonical protein kinase fold (PKL-fold) due to the presence of an N-terminal ATP binding lobe as well as due to a C-terminal substrate-binding lobe, and these features are making FN3K as specific for docking or for obtaining a specific template for homology modeling of human FN3K using SWISS EXPASY tools [25]. Hence, we developed a homology model of human FN3K using a sequence template of AtFN3K. The amino acid sequence of Human FN3K was blasted against the PDB-BLAST database to identify a suitable template for homology modeling. In our study, we found 50 templates and 3 best sequence homology models viz., model-1 with 40.67% (oligostate: homodimer), model-2 with 38.57% (oligostate: monomer), and model-3 with 26.2% (Oligostate: monomer) respectively. Among them, the sequence homology model-1 between the human FN3K and the selected template of FN3K of *Arabdosis thaliana* was found to be 40.67% as the highest (**Fig 1A**) and it could be a valid target for a drug after validation as minimum 30% of sequence similarity is required for CADD and MD simulations [13, 24].

### Validation

**Fig 2:**
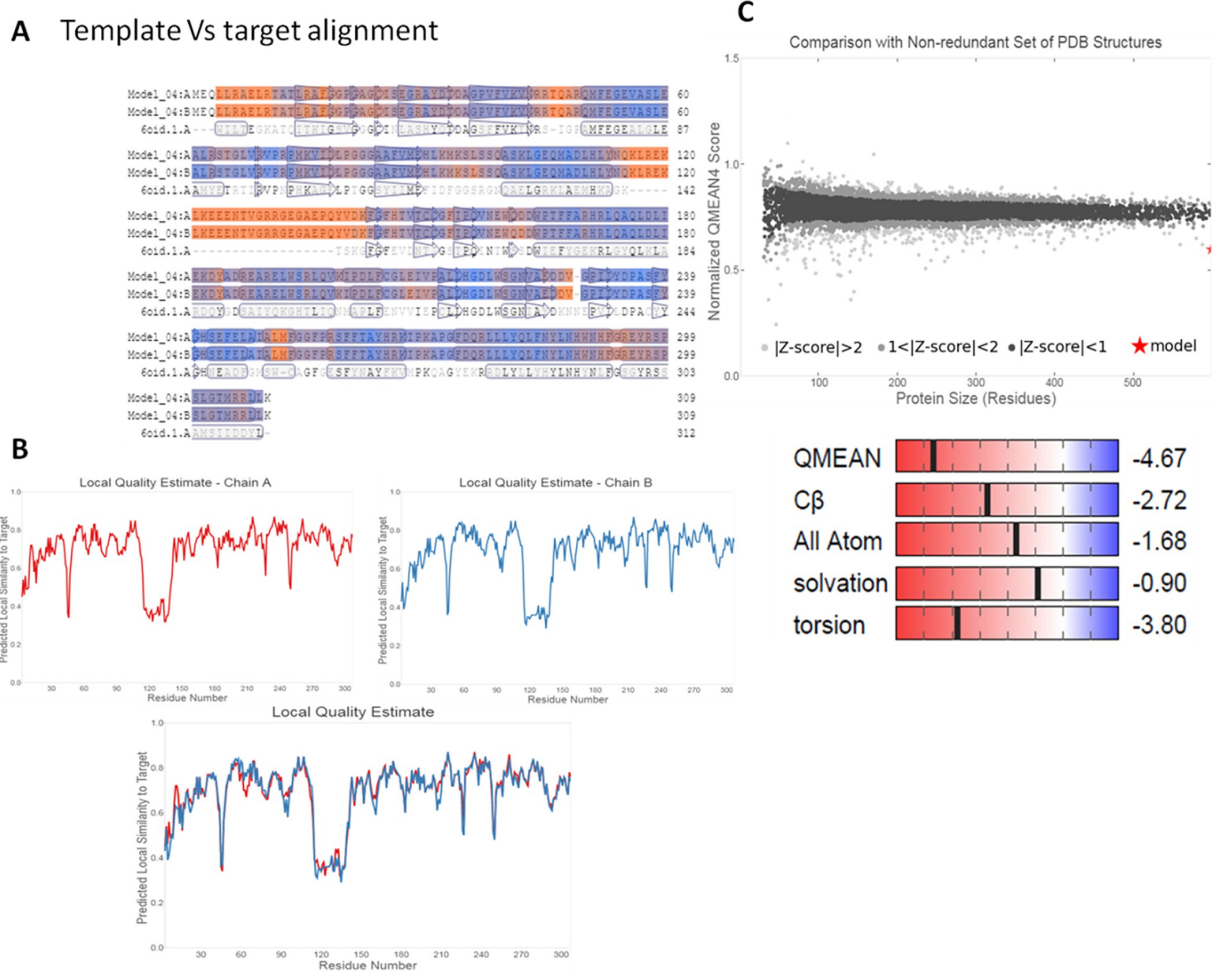
**(A).** Human FN3K target alignment with the template of AtFN3K. **(B).** The Local quality estimates of chain A and chain B for the homology of ‘human FN3K homodimer’. **(C).** Quality estimates by Z score and QMEAN of the FN3K homology homodimer with 40.67% seq. similarity.

**Fig 3:**
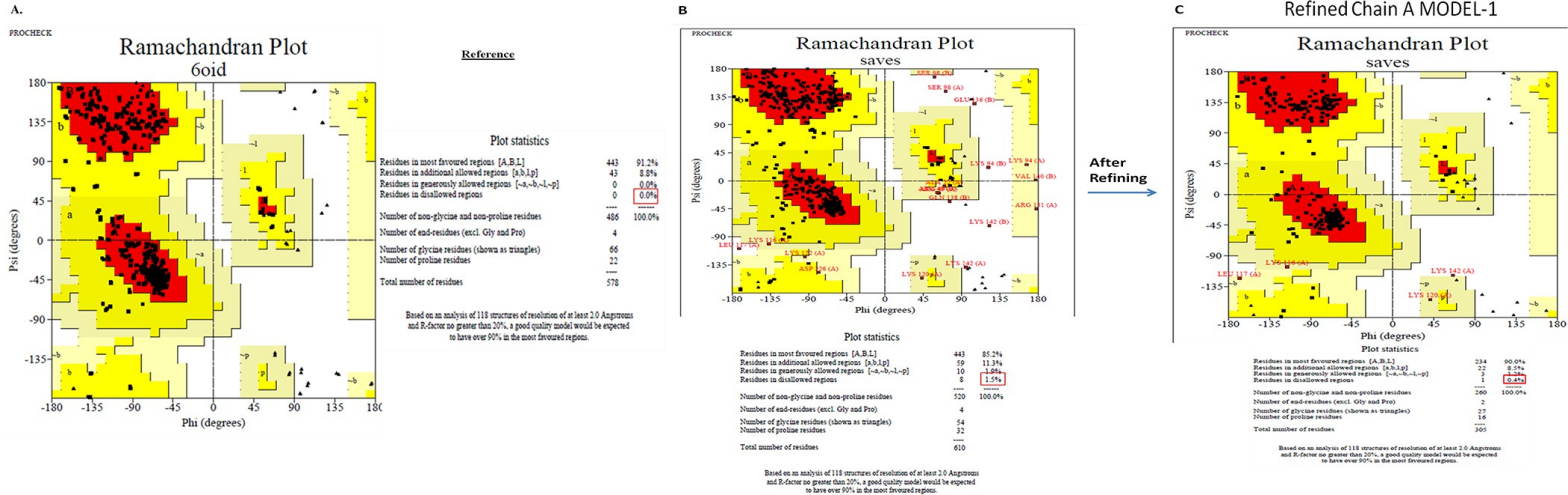
Chain-A ‘validation’ of the homology model of a 3D homodimer of FN3K using ProCHECK and Ramachandran plots, where the disallowed regions were completely minimized to 0.4% in Refined Chain A MODEL-1 of FN3K as depicted **(C)** compared to the non-refined model **(B)**, and reference model of *A thaliana* **(A)**. **Refined Chain A Model-1** was chosen out of 5 models (**S2 Fig.)** produced from ‘The galaxyweb protein refinement tool’ and comparatively considered as the **best model**, which was validated by the Ramachandran plots via ProCHECK and this was further used for molecular docking and MD simulation [9].

**Fig 4:**
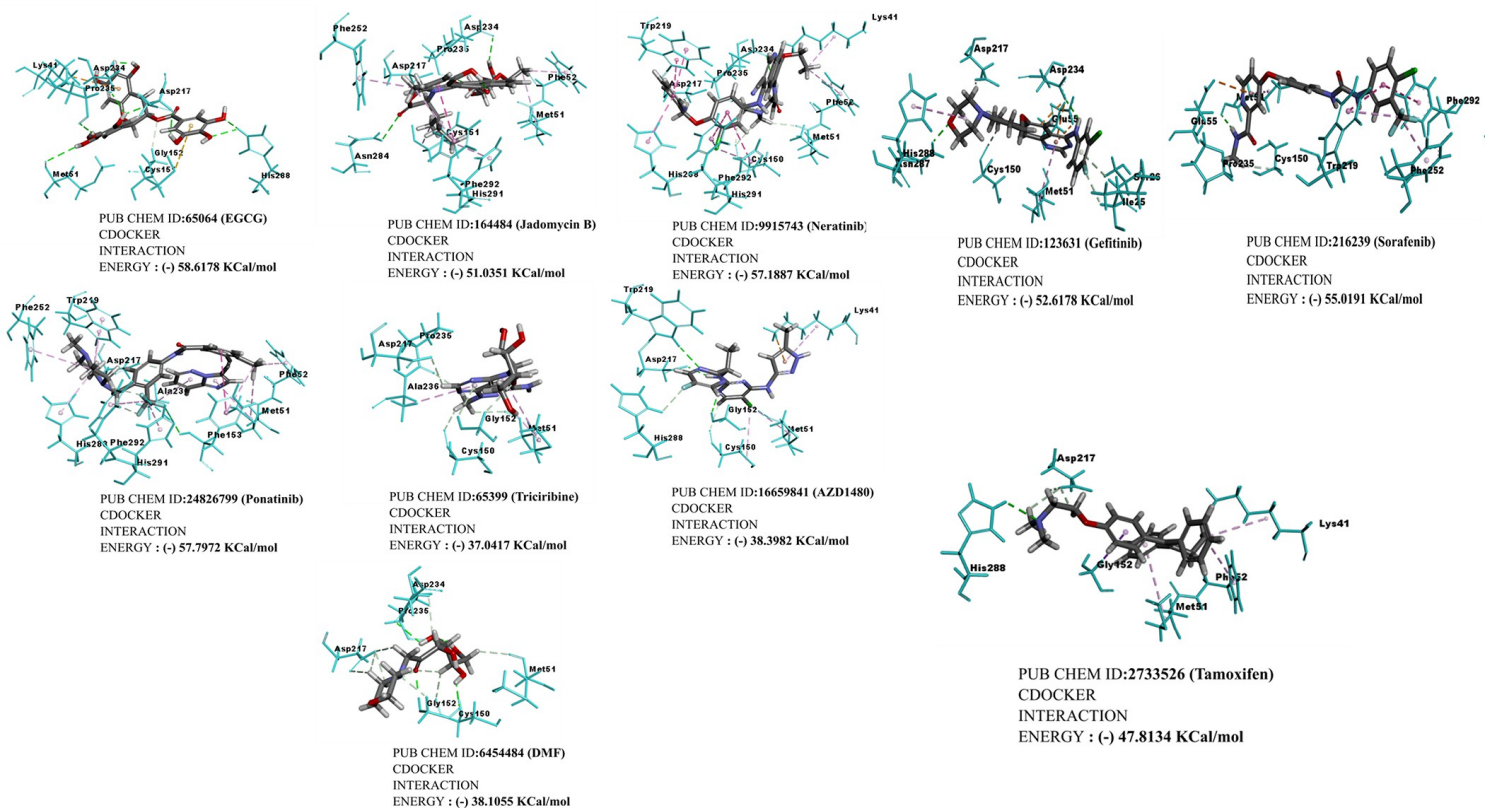
Contour maps were generated by the ‘3D model of FN3K chain A of homodimer’ with the optimal binding interaction of screened natural (EGCG, Jadomycin B) and synthetic (neratinib, AZD1480, gefitinib, sorafenib, tamoxifen citrate, ponatinib, tricirbine, DMF, an FN3K inhibitor) anticancer molecules represented in ball and stick models. These molecules interacted with active sites of chain A homodimer was shown by (-) CDOCKER interaction energy; and the interaction energies for the individual docked molecules were given along with Pubchem IDs. Coordination bonds and hydrogen bonds were depicted in dashed lines [8].

1. Clustal W analysis of multiple sequence alignment between human FN3K and *A thaliana* was given in **Fig 1B,** and a cladogram for phylogenetic similarity was depicted in **Fig 1B** [13].
2. Selected model-1 of FN3K has shown GMQE score of 0.70 and QMEAN score of −4.67 (**Fig 2A–2C**) [10].
3. Before the refinement using Galaxyweb, selected model-1 showed 8**%** residues in the disallowed regions of Ramachandran plots. After refinement using Galaxyweb (5 models in **S2 Fig**), the model-1 in PROCHECK validation of Ramachandran plots described the presence of only 0.4% residues in the disallowed regions (**Fig 3B and 3C**).
4. The complete FASTA sequence of *A*. *thaliana* (6OID) was taken as a ‘reference’. We have assessed the ‘3D model’ of this monomer and constructed Ramachandran plots (0% of residues in disallowed regions) for comparison (**Fig 3A**). In conclusion, ‘Modelled & Refined Chain A MODEL-1 of FN3K homodimer’ is considered the ideal model for molecular docking and MD simulation. We performed the molecular docking with DMF (a selective FN3K inhibitor), >50 synthetic and ≥50 natural kinase inhibitors onto the ‘Modelled & Refined Chain A MODEL-1 of FN3K homodimer’. [Note: we have obtained the best model of Chain A of FN3K modeled homodimer, attached as supplementary (**S2 Fig)**]. We performed molecular docking for the 3D model of AtFN3K as a reference standard attached as supporting information **(S1 Fig)** and also performed the docking of DMF onto the homology modeled FN3K (**Fig 4**).

### Molecular docking results

**Fig 5:**
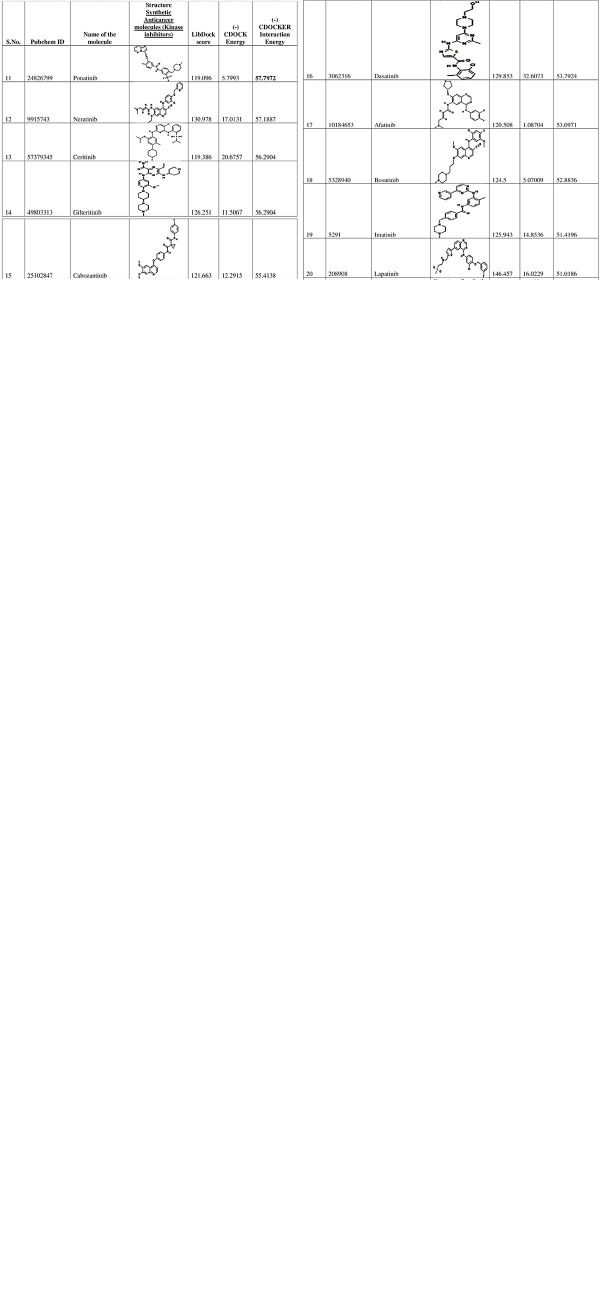
(-) CDOCKER interaction energies for the docked molecules onto homology modelled 3-dimentional human FN3K.

The anticancer molecules were screened after docking with the modeled human FN3K and CDocker score, and CDocker interaction energies were described for benzoic acid derivatives, cinnamic acid derivatives, synthetic & natural kinase inhibitors, and reference kinase inhibitor molecules. As a preliminary docking process, 40 molecules out of ≥ 100 molecules were screened according to the CDOCKER interaction energies (**Fig 5**) subsequently, molecular docking for 10 molecules was executed which resulted in identifying natural and synthetic anticancer small molecules as modulators for the activity of FN3K protein model (**Fig 4**) with most stable conformations in static mode of interactions of ligands in the binding pocket of the protein.

### CDocker interaction energies

of synthetic anticancer kinase inhibitors and DMF, a competitive FN3K inhibitor were selected as they could bind to the active pockets of modeled human FN3K with substantial binding energies (**Table 1**). Additionally, we have attached **S1 Fig** in which the DMF interaction energies when interacted with active sites of reported three-dimensional FN3K protein (PDB ID: 6OID) of *A*. *thaliana*.

**Table 1:** Hydrophobic and hydrogen bonding interactions of amino acid residues related to anticancer molecules with 3-dimensional homology modeled human FN3K, a deglycating enzyme of oncogenic Nrf2.

S.No.	Compounds	Pubchem ID	Interacting amino acid residues in catalytic ATP binding loop of human homology modeled FN3K	(-) CDOCKER interaction energy (Kcal/mol)
1	EGCG	65064	**Lys 41**, **Asp 234**, **Pro 235**, **Asp 217**, **Met 51**, Gly 152, Cys 15, His 288	58.61
2	Jadomycin B	164484	**Asp 234**, Phe 252, Pro 235, Asp 217, Phe 52, Asn 284, Phe 292, Cys 151, His 291, Met 51	51.03
3	Neratinib	9915743	Trp 219, Asp 234, Lys 41, Asp 217, Pro 235, His 208, Phe 292, His 291, Lys 150, Met 51, Phe 52	57.18
4	Ponatinib	24826799	Phe 252, Trp 219, Asp 217, Ala 23, Phe 52, Met 51, **Phe 153**, His 291, His 238, Phe 292	57.79
5	Tricirbine	65399	Pro 235, Asp 217, Ala 236, Gly 152, Met 51, Cys 150	37.04
6	AZD1480	16659841	**Trp 219**, Asp 217, His 288, **Gly 152**, Met 51, Cys 150, Lys 41	38.39
7	Sorafenib	216239	Glu 55, **Met 51**, Phe 292, Pro 235, Cys 150, Trp 219, Phe 252, Phe 292	55.01
8	Tamoxifen	2733526	Asp 217, **His 288**, Gly 152, Met 51, Phe 52, Lys 41	47.81
9	DMF	6454484	Asp 234, **Pro 235**, Asp 217, Met 51, **Gly 152**, **Cys 150**	38.10
10	Gefitinib	123631	Asp 217, Asp 234, **His 288**, Asn 287, Glu 55, Cys 150, Met 51, Ile 25, Ser 26	52.61

*Note: Bold and underlines in the above table correspond to amino acids with hydrophobic interactions depicted in the bright green color in **Fig 4**. and other colored interactions are hydrophobic or dipole interactions depicted between the three-dimensional homology modeled human FN3K with the anticancer molecules, and DMF respectively.

### ADME predictions of the screened anticancer molecules

Drug likeliness was ascertained by examining physicochemical properties as well as by applying Lipinski’s rule of five (**Table 2**). As per this rule, the molecular weight for any ideal molecule should be below 500 Daltons, H-bond donor < 5, ad H-bond acceptors < 10, as well as the log P value should be less than 5. The aqueous solubility (QPlogS) was in the acceptable range for sorafenib, ZD1480, and tamoxifen citrate, whereas the partition coefficient QPlogPo/w was within the acceptable range for sorafenib, jadomycin B, AZD 1480, neratinib, gefitinib, tamoxifen citrate, and ponatinib. The majority of the screened anticancer molecules can cross the blood-brain barrier and all the values were described in **Table 2**.

**Table 2:** ADME properties of screened anticancer molecules of natural and synthetic origin docked onto human FN3K model protein [23].

	Molecule ^a^	Pubchem ID	Molecular Weight (g/mol)	(-) CDOCKER Interaction energy (Kcal/mol)	TPSA (Topological Polar surface area) (A°)^2^	QPlogPo/w^b^	QPlogS^c^	QPPCaco^d^	Blood–brain barrier permeability	% Plasma protein Binding (PPB)	Lipinski’s Rule
Natural kinase Inhibitor	EGCG	65064	458.37	58.6178	197.37	1.53	-3.56	-6.652	Yes	87.686%	0 (No violation)
Natural kinase Inhibitor	Jadomycin B	164484	549.57	51.035	142.83	3.96	-5.64	-5.585	No	90.56%	1 violation
Reference molecule	Tricirbine or tricyclic phosphate (API-2)	65399	321.30	37.0417	144.47	1.03	-1.06	-6.034	No	37.088%	0 (No violation)
Reference molecule	AZD1480	16659841	348.77	38.3982	104.30	2.10	-3.68	-3.217	Yes	76.977%	0 (No violation)
Synthetic kinase Inhibitor	Neratinib	9915743	557.04	57.1887	112.40	3.96	-5.98	-3.287	Yes	89.826%	1 violation
Synthetic kinase Inhibitor	Gefitinib	123631	446.9	52.61	109.32	2.88	-4.32	-2.025	Yes	82.67%	0 (No violation)
Synthetic kinase Inhibitor	Sorafenib	216239	464.8	55.01	115.43	3.04	-2.98	-4.543	Yes	84.73%	1 violation
Synthetic kinase Inhibitor	Tamoxifen citrate	2733526	563.6	47.81	147.37	2.32	-3.63	-3.645	Yes	89.02%	0 (No violation)
Synthetic kinase Inhibitor	Ponatinib	24826799	532.56	57.79	65.77	4.42	-5.73	-3.040	Yes	93.802%	1 violation
FN3K Inhibitor	DMF (1-Deoxy-1-morpholino fructose)	6454484	265.26	38.1095	130.69	1.05	1.08	-6.029	No	15.43%	0 (No violation)

^a^Newly docked molecules.

^b^Predicted octanol/water partition coefficient log P (Acceptable range _2.0 to 6.5)

^c^Predicted aqueous solubility in mol/L (Acceptable range >-4).

^d^Predicted Caco cell permeability in nm/s (Acceptable range: >-5.15 is great).

### Molecular dynamic simulations

**Fig 6:**
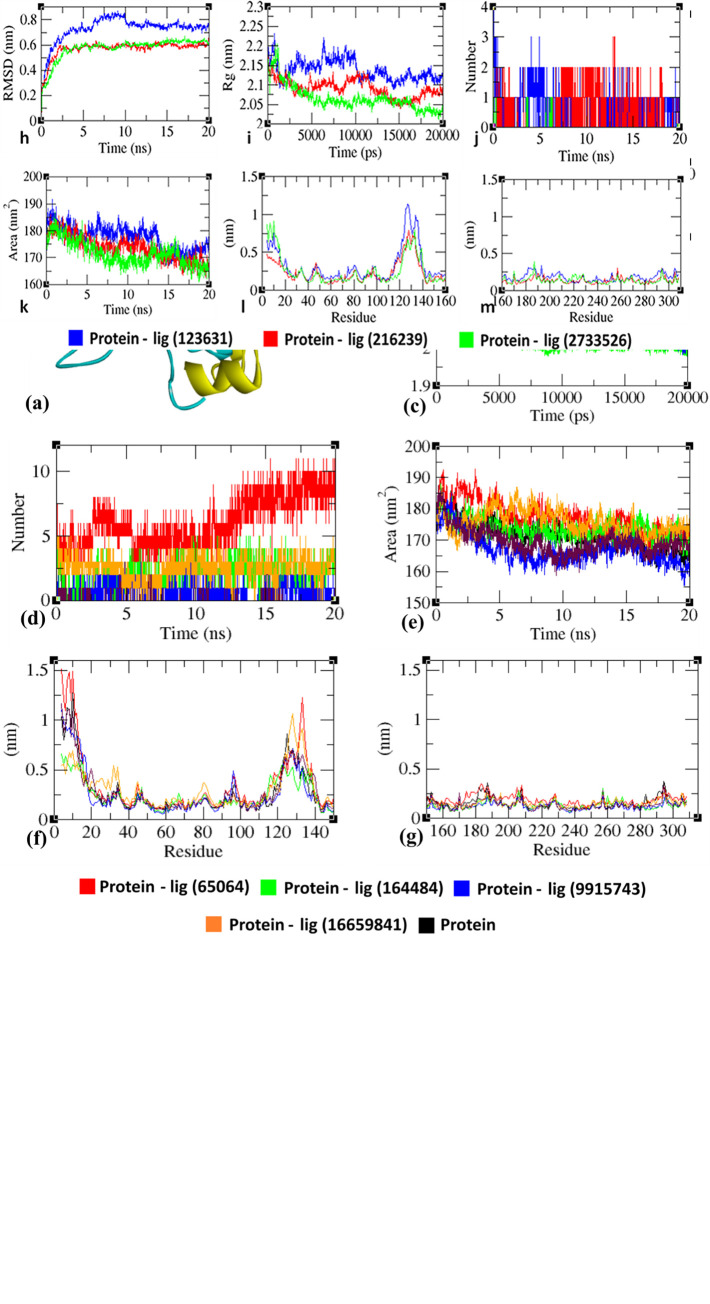
**(a).** Protein structure alignment of homology built FN3K model (cyan) with MEK1 PDB structure (yellow) (**PDB ID: 4U7Z**). Analysis of RMSD, *Rg*, hydrogen bond, SASA, and RMSF of FN3K homology model (protein) with 3g (ligand) complexes at 20000 ps (20 ns). **(b,h).** Time evolution of backbone RMSD of the FN3K-model protein (chain A of homodimer) alone and with kinase inhibitors of natural (EGCG Pubchem ID: 65064, Jadomycin B Pubchem ID: 164484), synthetic (neratinib Pubchem ID: 9915743, gefitinib Pubchem ID: 123631, sorafenib Pubchem ID: 216239, tamoxifen citrate Pubchem ID: 2733526) and reference ligand (AZD1480, Pubchem ID: 16659841) complexes. **(c, i).** Radius of gyration (*Rg*) of the protein backbone in its free and complex form over the entire simulation time. The ordinate is *Rg* (nm) and the abscissa is the time (ps) interval. **(d, j).** Hydrogen bonds occurring over the time (ns) of simulation between model protein and ligand. **(e, k)** SASA is indicated, where the ordinate is SASA (nm) and the abscissa is time (ns). **(f, l)** and (**g, m).** Residue-wise average RMSF plot of protein in the innate and ligand-bound form [8, 13, 76, 77].

Initially, we have examined the protein structure alignment of the ‘homology built FN3K model’ (cyan) with MEK1, a potent oncogenic kinase that has similar structural homology as FN3K (PDB structure (yellow) (**PDB ID: 4U7Z**) (**Fig 6A**). This alignment described the ATP binding site in modeled FN3K as the most significant region for the binding of the following molecules. The multikinase inhibitors which we have chosen above include neratinib: (-) 57.18 Kcal/mol (PubChem ID: 9915743), AZD1480: (-) 38.39 Kcal/mol (PubChem ID: 16659841), jadomycin B: (-) 51.03 Kcal/mol (PubChem ID: 164484), tamoxifen citrate: (-) 47.81 Kcal/mol (PubChem ID: 2733526), sorafenib: (-) 55.01 Kcal/mol (PubChem ID: 216239), ponatinib: (-) 57.79 Kcal/mol (PubChem ID: 24826799), gefitinib: (-) 52.61 Kcal/mol (PubChem ID: 123631), tricirbine: (-) 37.04 Kcal/mol (PubChem ID: 65399), and natural kinase inhibitor, EGCG (PubChem ID: 65064)’ exhibit a significant interacting ability with the human FN3K homology modeled protein.

Preliminary MD simulation for a 20 ns time frame was found useful for all the above molecules to draw a correlation between molecular docking and dynamic stability of protein-ligand complexes; however overall understanding of the attainment of stable conformation from the dynamics nature was achieved by extending the production run for 100 ns time frame for the specific molecules such as **sorafenib** and **gefitinib** which have significant (-) CDocker interaction score with the homology modeled 3-dimensional human FN3K.

In molecular dynamic (MD) simulation studies, the trajectory analysis (**Fig 6**) of protein-ligand complexes could conclude specific conformational changes occurring in the complexes during MD simulation. Dynamic nature was produced by the solvated protein systems which consequently generate fluctuations from initial conformations; hence, the **root mean square deviation (RMSD**) maps out the fluctuations and delivers the plot in time scale and provided ‘specific estimations pertinent to equilibrium as well as protein stability’ (**Fig 6B and 6H**) of the backbone C-α atoms that the apoprotein and ligand-bound protein complex reached equilibrium approximately at **4.5 to 5.0 ns time** and remaining were evident of stable trajectory with minimal deviation in **~0.10 to ~0.15 nm RMSD range**; this was indicating the attainment of the structural rigidity of protein being bound to ligand than its free form. The unbound protein exhibited stable trajectories, whereas, ligand-bound proteins reached equilibrium only after initial fluctuations during the simulation.

The **radius of gyration (R**_**g**_**)** was considered a significant parameter that depicts the altered masses calculated to root mean square distances while in motion around the central axis of rotation. R_g_ plot (**Fig 6C and 6I)** was indicative of capability, shape, and folding at the time of motion at each time step of the entire trajectory. The protein was in its ‘apo form’ and it was found with a specific reduction in gyration terms ~0.25 nm allying to the ligands bound protein showing stable trajectories only fluctuating in ~0.15 nm distances.

**H-bonds** are the significant characteristic for any protein-ligand interactions to evaluate in terms of stability. The biological functions were analyzed over the total simulation period. The intermolecular H-bond plot (**Fig 6D and 6J**) in time scale indicates that the number of H-bonds remains consistent with molecular docking, while simultaneously breaking and reforming of bonds being observed.

**Solvent accessible surface area (SASA)** measures the area around the hydrophobic core formed between protein-ligand complexes (**Fig 6E and 6K)**. Consistent SASA values were observed typically fluctuating in between ~20–40 nm^2^ areas.

**Fig 7:**
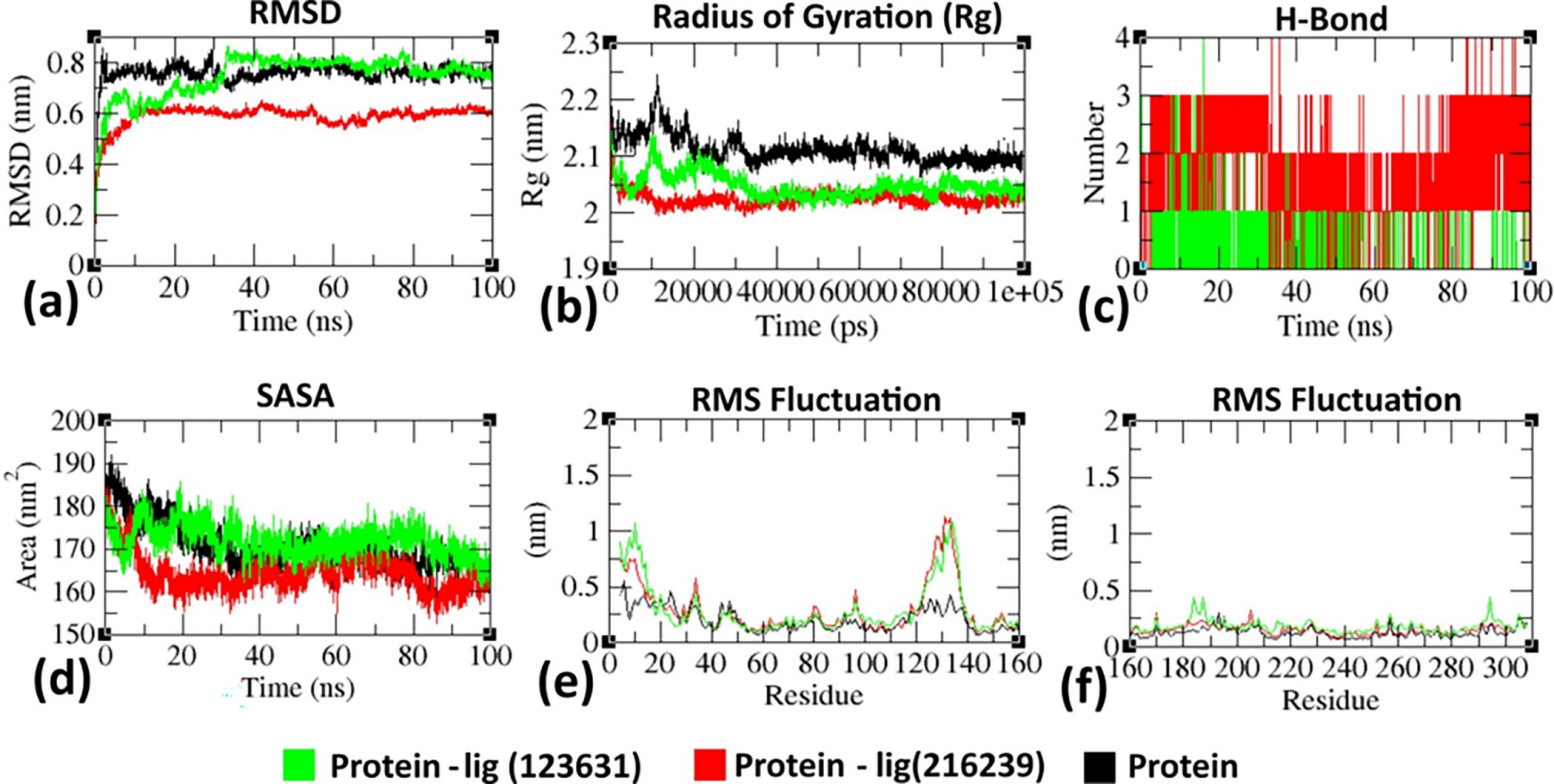
Analysis of RMSD, *Rg*, hydrogen bond, SASA, and RMSF of FN3K homology model (protein) with gefitinib (Pubchem ID: 123631), sorafenib (Pubchem ID: 216239) complexes at 100000 ps (100 ns) respectively. **(a)** Time evolution of backbone RMSD pertinent to the FN3K model protein (chain A of homodimer) alone and FN3K-gefitinib, FN3K-sorafenib complexes. **(b)** The radius of gyration (*Rg*) of the protein backbone in its free and complex form over the entire simulation time. The ordinate is *Rg* (nm) and the abscissa is the time (ps) interval. **(c)** Hydrogen bonds occurring over the time (ns) of simulation between FN3K model protein and ligand. **(d)** SASA was indicated, where ordinate was SASA (nm) and abscissa was time (ns). **(e, f)** Residue-wise average RMSF plot of protein in the innate and ligand-bound form [8, 13, 76, 77].

The residue-wise fluctuations can be evident from the root mean square fluctuation (RMSF) plot (**Fig 6F, 6L, 6G and 6M**), where amino acids are rigidified in the complex form as compared to the innate state of the protein. RMSD, *Rg*, hydrogen bond, SASA, and RMSF of FN3K homology model (protein) with gefitinib (Pubchem ID: 123631), sorafenib (Pubchem ID: 216239) complexes at 100000 ps (100 ns) were given in **Fig 7A–7F**.

Along with other MD analysis, SASA was employed to access the solvent interactions with the protein and its complexes, which ultimately outline the information about the protein’s 3D conformation. Total SASA provides us with the solvent accessibility of the complex as a whole, whereas the buried SASA indicates the stability of the interface as a measure of the binding surface between the protein and the ligand which is formed by the binding of the ligand to the protein. **Table 3** described both the complexes such as FN3K-gefitinib and FN3K-sorafenib with stable trajectories in a similar trend with minor perturbations.

**Table 3:** SASA values were given for the homology modeled 3-dimensional human FN3K, FN3K-gefitinib, and FN3K-sorafenib complexes.

System	Protein SASA (nm^2^)	Ligand SASA (nm^2^)	Complex SASA (nm^2^)	Buried SASA (nm^2^)
FN3K	169.435	-	169.435	-
FN3K- gefitinib	171.673	7.563	171.361	8.592
FN3K- sorafenib	172.163	8.034	172.046	8.844

To further examine the binding efficiency of the synthetic hits (**gefitinib** and **sorafenib**) on FN3K protein, binding free energy calculation by MM/PBSA was carried out and the results were listed in **Table 4**. According to the MM/PBSA calculation, **sorafenib** exhibited higher binding affinity with -29.23 ± 5.42 KJ/mol to FN3K protein when compared to **gefitinib** having an affinity of—24.45 ± 7.21 KJ/mol. Among the other four terms, ΔG_polar_ and ΔG_nonpolar_ have contributed similarly, while ΔE_vdw_ and ΔE_electrostatic_ differed considerably in both cases because of the changes in Vander Waals and electrostatic interactions due to variation in the intermolecular distances. The final result of the simulation provides insights into the bioactive nature of all the ligands.

**Table 4:** Values of MM/PBSA free energies pertinent to FN3K-gefitinib and FN3K-sorafenib complexes by MD simulations.

System	ΔE_vdw_	ΔE_electrostatic_	ΔG_polar_	ΔG_nonpolar_	ΔG_binding_
	(KJ/mol)	(KJ/mol)	(KJ/mol)	(KJ/mol)	(KJ/mol)
FN3K- gefitinib	–128.21±5.76	–23.28±3.27	–47.28±6.56	–17.12±4.92	– 24.45±7.21
FN3K- sorafenib	–130.41±5.81	–22.24±5.15	–43.17±6.03	–18.42±5.26	– 29.23±5.42

Therefore, MD simulation studies confirmed the presence of stable H-bonding interactions. Among the molecular dynamics of anticancer molecules such as ‘neratinib, AZD1480, Jadomycin B, tamoxifen citrate, sorafenib gefitinib, and natural multikinase inhibitor, EGCG ‘, the SASA values and MM-PBSA values were ascertained for FN3K-gefitinib and FN3K-sorafenib complexes respectively. Thus, the summed-up result of the simulation provides insights into the bioactive nature of ligands towards FN3K homology-built protein.

**Fig 8:**
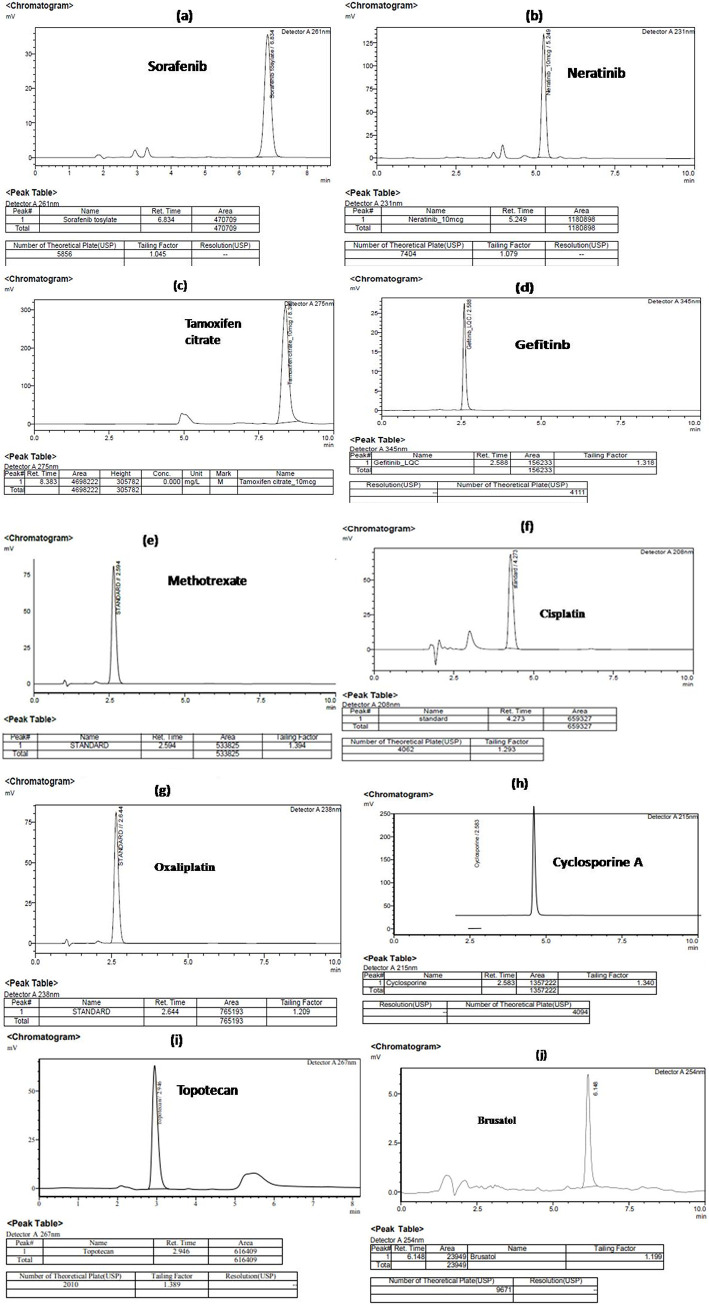
HPLC characterization of the anticancer molecules. The chromatographic peaks were observed which denotes the characteristic identification of drug molecules such as (a) sorafenib, (b) neratinib, (c) tamoxifen citrate, (d) gefitinib, (e) methotrexate, (f) cisplatin, (g) oxaliplatin, (h) cyclosporine A, (i) topotecan, (j) brusatol respectively.

Before the in vitro assays, the identification, and characterization of the selected anticancer molecules viz., such as sorafenib, neratinib, tamoxifen citrate, gefitinib, methotrexate, cisplatin, oxaliplatin, cyclosporine A, topotecan, brusatol at specific optimized chromatographic conditions in HPLC method were depicted in **Fig 8**.

### IC-50 activity determination for screened anticancer molecules

**Fig 9:**
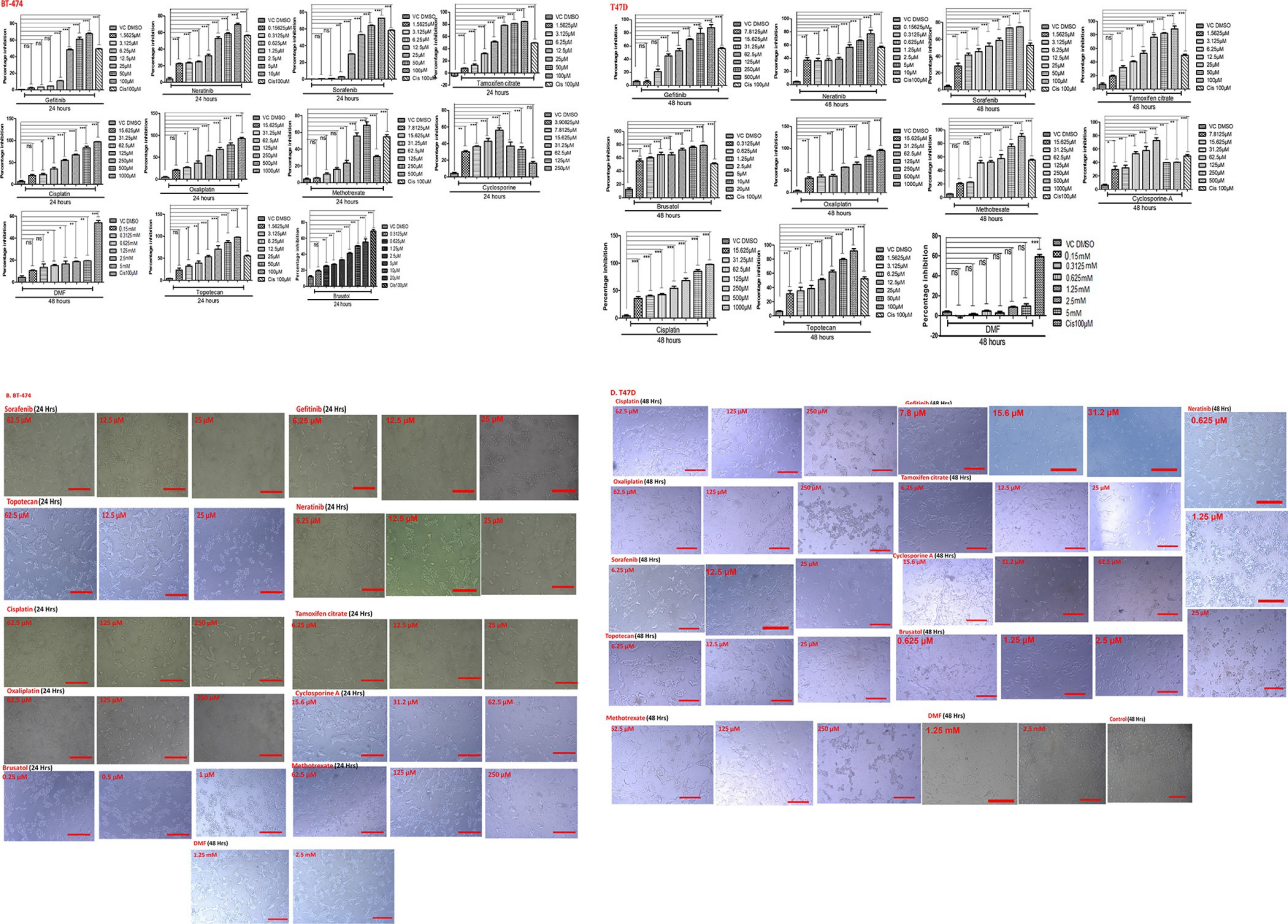
**(A), (B)**. Cytotoxicity: BT-474 and T47D breast cancer cells were cultured and seeded into 96 well plates (10000 cells/well) subsequently subjected to incubation for 24 and 48 hours respectively. Later, the cells were treated for drugs (**BT-474/T47D**: neratinib: 10 μM, 5 μM, 2.5 μM, 1.25 μM, 0.625 μM, 0.312 μM, 0.156 μM; sorafenib/tamoxifen citrate/topotecan: 1.5 μM, 3.125 μM, 6.25 μM, 12.5 μM, 25 μM, 50 μM, 100 μM; cisplatin/oxaliplatin: 15.6 μM, 31.25 μM, 62. 5 μM, 125 μM, 250 μM, 500 μM, 1000 μM); **BT-474**: gefitinib: 1.5 μM, 3.125 μM, 6.25 μM, 12.5 μM, 25 μM, 50 μM, 100 μM, methotrexate: 7.8 μM, 15.6 μM, 31.25 μM, 62. 5 μM, 125 μM, 250 μM, 500 μM; cyclosporine A: 3.9 μM, 7.8 μM, 15.6 μM, 31. 2 μM, 62. 5 μM, 125 μM, 250 μM; Brusatol: 20 μM, 10 μM, 5 μM, 2.5 μM, 1.25 μM, 0.312 μM, 0.625 μM,; **T47D**: gefitinib: 7.8 μM, 15.6 μM, 31. 2 μM, 62. 5 μM, 125 μM, 250 μM, 500 μM; cyclosporine A: 7.8 μM, 15.6 μM, 31. 2 μM, 62. 5 μM, 125 μM, 250 μM, 500 μM; methotrexate: 15.6 μM, 31.25 μM, 62. 5 μM, 125 μM, 250 μM, 500 μM, 1000 μM; Brusatol: 20 μM, 10 μM, 5 μM, 2.5 μM, 1.25 μM, 0.625 μM, 0.312 μM; vehicle control <1%; cisplatin (100 μM) was used as a positive control for 24 hours and 48 hours respectively. DMF (known FN3K inhibitor): 0.15 mM, 0.31 mM, 0.625 mM, 1.25 mM, 2.5 mM, 5 mM for 48 hours. (*p< 0.05 compared to control, **p< 0.01, ***p<0.001 compared to control, NS: nonsignificant). [Note: drug precipitation occurred at higher concentrations of cyclosporine treatment in both the cell lines]. **(B),(D).** Morphological changes such as cell shrinking, cell rounding was observed in a dose-dependent fashion when the T47D, BT-474 cells were treated with different anticancer drug molecules. In case of DMF treatment, the cytostatic effect was observed in both the cell lines as there was no 50% cell death. Magnification: 4x, 10x.

IC-50s for potent kinase inhibitors including sorafenib, tamoxifen citrate, neratinib, gefitinib, and other anticancer drugs including cisplatin, oxaliplatin, cyclosporine A, and methotrexate were identified for BT-474 and T-47D cell lines respectively (**Table 5**). Additionally, we have performed cytotoxicity for anticancer phytochemicals including topotecan, and brusatol on these breast cancer cell lines (**Fig 9**) subsequently the selected concentrations were used for treatment onto BT-474, T-47D cell lines for Western blotting to examine the expression patterns of FN3K, Nrf2 antioxidant downstream signaling proteins.

**Table 5:** IC-50 concentrations of the screened anticancer molecules against breast cancer cell lines including BT-474, and T47D.

	IC-50 (μM)—SRB assay	
Anti-breast cancer molecules	BT-474 (24 hours)	T-47D (48 hours)
Neratinib	2.5 μM	2.5 μM
Sorafenib tosylate	25 μM	25 μM
Tamoxifen citrate	12.5 μM	12.5 μM
Gefitinib	25 μM	62.5 μM
Cisplatin	125 μM	125 μM
Oxaliplatin	125 μM	125 μM
Cyclosporine A	31.25 μM	31.25 μM
Topotecan	12.5 μM	12.5 μM
Brusatol	10 μM	10 μM
Methotrexate	62.5 μM	62.5 μM

### Differential expression patterns of FN3K and Nrf2 antioxidant signalling

We specifically selected anticancer molecules including sorafenib, neratinib, tamoxifen citrate, gefitinib, methotrexate, cisplatin, oxaliplatin, and cyclosporine A to examine the expression patterns of FN3K, Nrf2-Keap1 and its downstream antioxidant signalling proteins in BT-474 cell lines. Expression of FN3K was significantly higher in ductal invasive carcinomas such as BT 474, T47D, and MCF-7.

**Fig 10:**
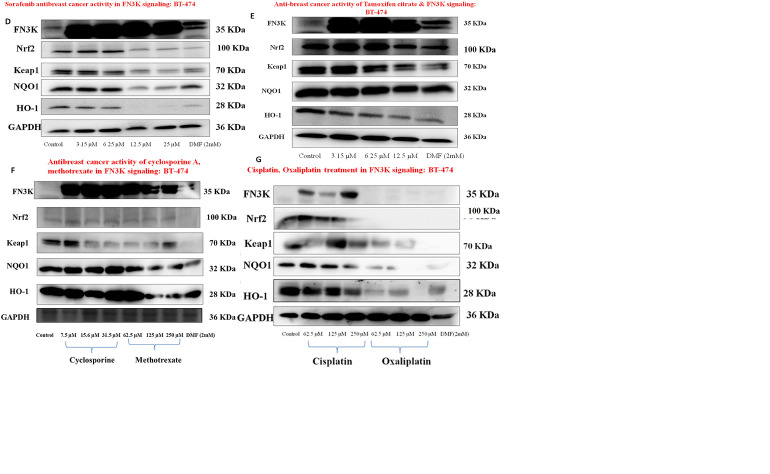
**A.** Western blot protein expression study delineated the substantial expression of FN3K in ductal invasive carcinomas such as BT-474, and T-47D (Luminal subtype) compared to the other molecular subtypes including Triple-negative breast cancers (TNBCs). In addition, the expression of Nrf2 and Keap1 was probed in TNBCs (MDA-MB-231, MDA-MB-468), MCF-7, and luminal molecular subtype cancers. FN3K expression in HepG2 cells was considered a positive control. **B**. BT-474 cells with neratinib treatment: Expression patterns of FN3K protein were increased followed by the enhancement in Keap1 expression with slight expression changes in Nrf2, subsequently the NQO1 and HO-1 antioxidant protein expression were minimized. **C, D, E**. BT-474 cells with gefitinib/sorafenib/tamoxifen citrate treatment: Expression patterns of FN3K protein were increased followed by the decline in Nrf2, Keap1, NQO1, and HO-1 antioxidant protein expressions. **F**. BT-474 cells with cyclosporine A/methotrexate treatment: Expression patterns of FN3K protein were increased followed by the decline in Keap1 compared to control without significant alteration in the Nrf2 expression pattern, whereas NQO1 and HO-1 antioxidant protein expression was not changed compared to control; In case of methotrexate, the expression of Nrf2 slightly decreased and Keap1, NQO1, the HO-1 expression pattern was downregulated compared to control. **G**. BT-474 cells with cisplatin/oxaliplatin treatment: expression of FN3K, Nrf2, and Keap1 was not completely declined with cisplatin but NQO1, and HO-1 expression was mitigated at higher concentrations; interestingly, oxaliplatin downregulated the expression of FN3K and Nrf2 consequently mitigated expression of antioxidant proteins such as NQO1 and HO-1. 1-deoxy-1-morpholinofructose (DMF) (dissolved in 50% ethanol), a competitive inhibitor of FN3K, was used as a positive control which could only inhibit ∼10% of total FN3K activity but the FN3K expression was not decreased upon DMF treatment [12]. GAPDH was used as an internal control in all the treatments. [Note: all the original blots were provided as supporting information files and attached as original blots-1, original blots-2 respectively].

Furthermore, the expression of FN3K is significantly upregulated when compared to control in all the drug-treated groups such as neratinib, sorafenib, gefitinib, tamoxifen citrate, cyclosporine A, and methotrexate. Consequently, the Nrf2 protein expression was downregulated in neratinib, sorafenib, tamoxifen citrate, and gefitinib, but no changes of expression were observed in the case of cyclosporine A or methotrexate. Keap1 expression patterns were significantly altered in all the drug-treated groups compared to the control. However, oxaliplatin completely downregulated FN3K expression in all the treated concentrations when compared to cisplatin. Nrf2 and Keap1 expression patterns also altered with these platinum derivatives in BT-474 cells. Other Nrf2 downstream antioxidant genes such as NQO1 and HO-1 were also mitigated in their expression patterns upon drug treatments (**Fig 10A–10G)**.

FN3K expression in T47D cells: Furthermore, we have examined the FN3K expression patterns alone in the T47D, another ductal invasive carcinoma cell line. Kinase inhibitors such as neratinib, sorafenib, gefitinib, tamoxifen citrate, and cyclosporine A enhanced the FN3K expression compared to control; topotecan, a camptothecin derivative of anticancer drug and methotrexate also upregulated the FN3K expression. Interestingly, both cisplatin and oxaliplatin treatment have downregulated FN3K expression (**S3 Fig)**.

Nrf2 inhibitor: Brusatol treatment onto BT-474, T-47D upregulated the expression of FN3K (**S3 Fig).**

### NQO1 activity measurements

**Fig 11:**
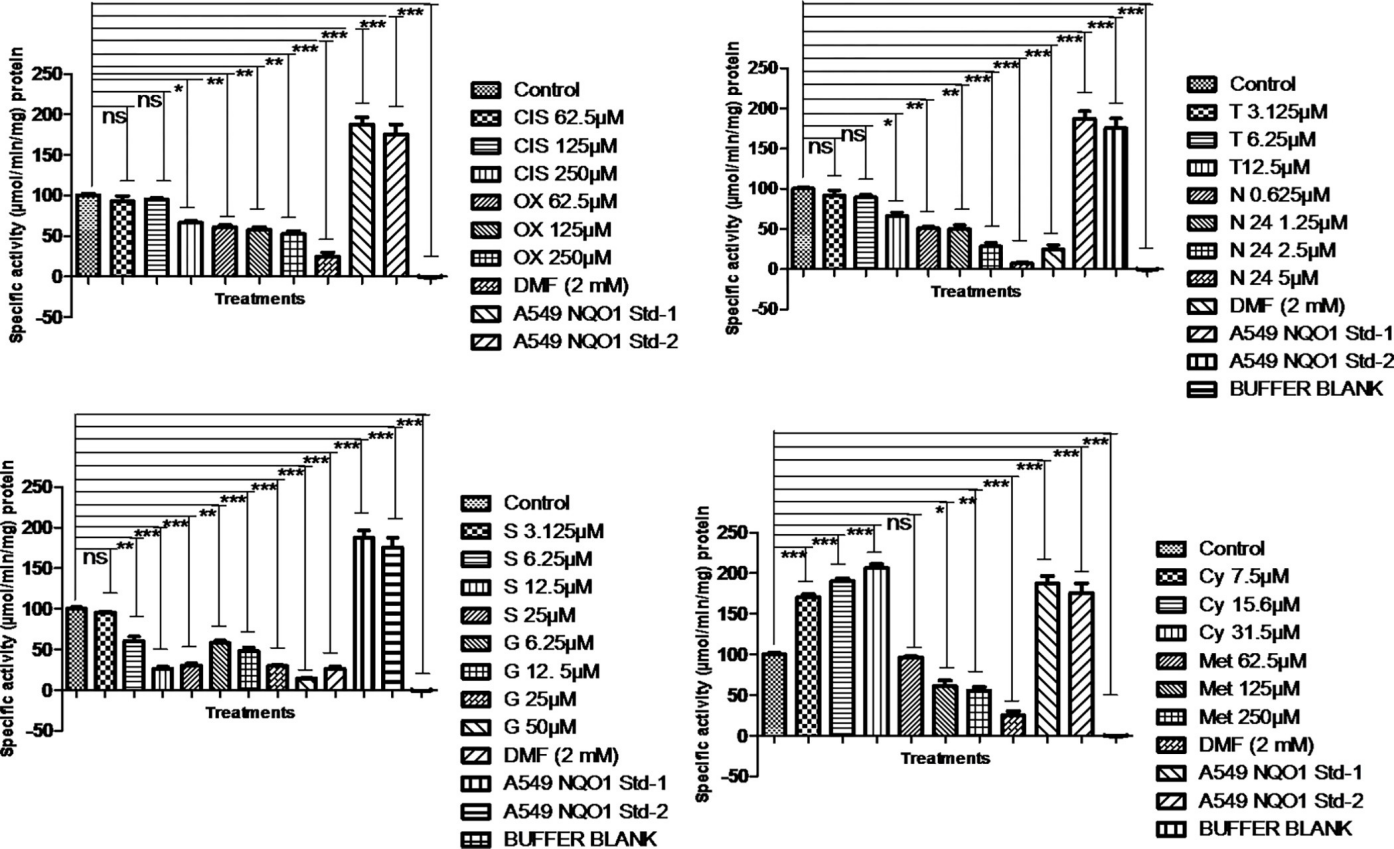
The activity of NQO1 was modulated with the treatments of sorafenib, tamoxifen citrate, neratinib, DMF, gefitinib, cisplatin, oxaliplatin, cyclosporine, and methotrexate. The NQO1 was declined in sorafenib, tamoxifen citrate, neratinib, DMF, gefitinib, cisplatin, oxaliplatin, methotrexate and although its activity enhanced with cyclosporine A but consequently declined in a dose-dependent manner compared to control. (*p< 0.05 compared to control, **p< 0.01, ***p<0.001 compared to control, NS: nonsignificant), A549 cell lysates for NQO1 activity as standards were used during the assay. S: sorafenib, T: Tamoxifen citrate, N: Neratinib, G: gefitinib, CIS: cisplatin, OX: Oxaliplatin, Cys: cyclosporine A, Met: methotrexate; DMF (2.5 mM), an FN3K inhibitor. Control: BT-474 lysates without treatment.

NQO1 activity was executed as per the procedures described by Prochaska, H.J et al and Shen, J. et al [26, 27]. Drug-treated lysates were analyzed for NQO1 activity and the specific drugs including sorafenib, tamoxifen citrate, neratinib, DMF, gefitinib, cisplatin, oxaliplatin decreased the NQO1 activity. Although NQO1 activity enhanced with cyclosporine A but consequently this enhancement was observed in a dose-dependent manner in cyclosporine A treatment and the patterns of NQO1 activity were declined with methotrexate when compared to control (**Fig 11**).

## Discussion

Breast carcinoma (BC) continues to exhibit resistance to the existing therapies due to dose-related toxicity and adverse effects on non-target tissues. Hence, identifying key protein(s) that play vital roles in the progression of breast carcinoma is highly significant. Protein glycation is minimally explored for the development of anti-cancer agents. A recent report by Sanghvi V., et al 2019 has demonstrated that the oncogenic activity of Nrf2 is regulated by deglycation by FN3K in hepatocellular carcinomas (HCCs) [4]. Thus, FN3K has a significant role in deglycating the Nrf2 in HCCs. Studies from our laboratory have demonstrated the upregulation of Nrf2 in breast cancers [6, 28]. In this study, we reported the FN3K modulators by screening several anticancer molecules subsequently docked onto the human FN3K protein homology model.

A report by Roskoski Jr R et al 2019 demonstrated the structure-dependent binding, three-dimensional catalytic domains of several protein kinases using homology modeling for ascertaining the ligand-binding efficacy of several FDA-approved kinase inhibitors [8]. Several protein kinase inhibitors are preferred for treating chronic diseases, cancers, and malignant and nonmalignant diseases [8]. The most significant targets of the kinase inhibitors include ‘ALK, B-Raf, BCR-Abl, epidermal growth factor receptor (EGFR), and vascular endothelial growth factor receptor (VEGFR) [8]. Human FN3K consists of an equivalent P-loop Cys, which is also redox sensitive [29]. Authors in this study reported a significant role for the “ATP binding site” in the redox regulation of FN3Ks. P-loop is stabilized in an extended conformation by “Cys-mediated disulfide bonds” connecting two chains to form a covalently linked dimer. The P-loop of FN3Ks is characterized by conserved Gly residues in addition to the redox-sensitive Cys residue; the presence of both these features might be necessary for the strand exchange dimer [8].

A study by Kornev et al described the active, conformations, and functional residues pertinent to several protein kinases with the aid of a local spatial alignment algorithm [30, 31]. According to their report, a catalytic spine is formed by the 8 hydrophobic amino acid residues whereas the regulatory spine is formed by four hydrophobic amino acid residues. These units are the stable and catalytic spine that could enable the positioning of ATP whereas the R-spine could interact with the protein substrate to mediate the catalytic reaction. A few studies by Liao and Van Liden et al segregated the amino acid residues between small and large lobes of protein kinases into the front cleft, gate area, and back cleft [32, 33]. The front cleft is composed of a glycine-rich amino acid loop and (HRD(x)_4_N) residues whereas the gate area is composed of an activation segment of DFG [8]. The back cleft region is formed by the αC-β4 back loop, β4- and β5-strands, and αE-helix located in the small lobe, and large respectively. Designing or docking therapeutic small molecule inhibitors to attain selectivity through drug interactions is a significant challenge in medicinal chemistry to mitigate disease processes including cancers [34–37]. Binding pockets located in the catalytic region of kinases could play a prominent role in predicting the efficacy of kinase inhibitors/activators or to design novel protein kinase inhibitors with utmost drug affinity. Previous studies described the ligand-binding affinity of several kinases like ‘cyclin-dependent kinase [38, 39], EGFR family of protein-tyrosine kinases [40–42], Janus kinase inhibitors [34], MEK1/2 dual specificity kinase inhibitors [43], Raf protein-serine threonine kinases [37], VEGFR1/2/3 protein-tyrosine kinases [8, 44].

In our study, FN3K is a kinase in which several kinase inhibitors and other anticancer molecules interacted through hydrogen bonding, hydrophobic interactions, and dipole interactions indicating a higher ligand-binding affinity. In the current study, we generated the homodimer of the FN3K homology 3D model with different sequence similarities and selected the chain A of the human FN3K 3D model with the highest sequence similarity (40.6%) for further validation using Ramachandran plots for the alignment of amino acids in beta helices and alpha helices. The template and target sequence of the human FN3K were validated phylogenetically using cladogram construction in Clustal W tools and Ramachandran plots. In the binding energies of the screened anticancer molecules through molecular docking onto the designed FN3K protein exhibited stable confirmations as indicated by the RMSD, Rg, and RMSF in the MD simulations. The kinase inhibitors are neratinib, sorafenib, gefitinib, tamoxifen citrate, AZD1480, Jadomycin B, and EGCG’ which exhibited a significant interacting ability with designed human FN3K modeled protein. Initially, molecular dynamics simulation run for 20 ns for anticancer molecules such as ‘neratinib, AZD1480, Jadomycin B, tamoxifen citrate, sorafenib gefitinib, and natural multikinase inhibitor, EGCG’ has attained a stable trajectory. However, we extended the simulation for 100 ns specifically for sorafenib and gefitinib with substantial binding efficacy with modeled FN3K and the stable trajectories were evident throughout the 100 ns duration. SASA values and MM-PBSA values were ascertained for FN3K-gefitinib and FN3K-sorafenib complexes respectively. The summed-up result of simulation related to the above molecules with modeled human FN3K provides insights into the bioactive nature of ligands towards FN3K homology-built protein. Nrf2 has a significant oncogenic role in several cancers due to its ability to get influenced by metabolic reprogramming to trigger antioxidant responses toward the chemotherapy [4]. The oncogenic role of Nrf2 is vividly responsible for tumor progression [5]. FN3K could enhance the deglycation of Nrf2 consequently increasing the antioxidant role of Nrf2 in HCC, and offering protection for cancer cells during chemotherapy [4]. Therefore, the development of small molecule inhibitors is a significant strategy to inhibit selectively FN3K in HCC, a strategy which may be used to target breast cancers. Kinase inhibitors such as sorafenib, gefitinib, neratinib, tamoxifen citrate, and platinum derivatives including cisplatin, oxaliplatin, other anticancer drugs such as cyclosporine A, & methotrexate, and brusatol, topotecan of natural origin were examined for the anti-breast cancer activity against luminal type breast cancer cell lines, BT-474, T-47D. Subsequently, we have screened and selected already proven anticancer molecules including neratinib, sorafenib, gefitinib, cyclosporine A, tamoxifen citrate, cisplatin, oxaliplatin due to their broad-spectrum activity and evaluated for their efficacy in modulating the FN3K expression in vitro. For instance, **sorafenib** is a multikinase inhibitor involved in impairing the autophosphorylation of receptor tyrosine kinases such as VEGFR1, 2, and 3, PDGFRβ, and RET as these receptor tyrosine kinases typically involved in angiogenesis and tumor progression even in breast cancers [45, 46]. A report by Sun, X. et al 2016 delineated that the blocking p62/Keap1/Nrf2 signaling resulted in enhanced sorafenib-induced HCC progression [47].

In our study, the expression of FN3K was substantially enhanced followed by the mitigation of Nrf2 expression and downstream target genes including HO-1, and NQO-1 with sorafenib. **Neratinib** is another multikinase inhibitor that can potentially block the activity of HER2, HER4, and EGFR and is reported to be implicated in breast cancers [48–51]. **Gefitinib** [52] is another EGFR-tyrosine kinase inhibitor [53] implicated against liver and lung cancers [52] and it has exhibited its efficacy in mitigating the breast cancer cell proliferation of MCF-7 and BT-474 [54, 55]. **Tamoxifen citrate** could inhibit the protein kinase C activity and is implicated as an anticancer drug as it has a significant role as a selective estrogen response modifier, and also an anti-angiogenic factor [56]. This drug can mitigate breast cancer progression [57–59]. However, the tamoxifen-resistance is one of the significant aspects in breast cancer cells and the resistant mechanisms are being maintained by the recurrent activation of Nrf2-mediated antioxidant response elements in breast cancer cells through ERK signaling [60]. **Cyclosporine A** is an enzyme pyruvate kinase inhibitor that substantially can impair breast cancer progression as pyruvate kinase enzyme expression is higher in the breast cancer cells like MCF-7, MDA-MB-231, and MDA-MB-435 [61, 62]. In the present study, treatment of these drugs against BT-474 at optimum dose ranges have mitigated the FN3K expression consequently reduced the Nrf2, HO-1, and NQO-1 expression patterns.

Other anticancer drugs like platinum derivatives including **cisplatin** and **oxaliplatin** were also examined against their ability for modulating the FN3K expression in breast cancers after determining their cytotoxicity [63–65]. A stable expression of Nrf2 in cancer cells could confer chemoresistance to cisplatin, etoposide, and doxorubicin [66]. Nrf2 activation through sulforaphane mitigated the cisplatin-invoked cancer cell death in Nrf2 proficient cells when compared to Nrf2-Cas9 cells [67]. FN3K expression and Nrf2 expression patterns were not mitigated with increasing doses of cisplatin in BT-474 cells. In the case of oxaliplatin [68], in the current study, the expression of Nrf2 and FN3K declined in a dose-dependent manner. **Methotrexate** is an immunosuppressant and is implicated to mitigate metastatic breast cancer [69] and inflammation as it can act as an antioxidant and scavenges superoxides, and it has been concluded that the methotrexate can inhibit Nrf2 activation in the cells treated with malondialdehyde and acetaldehyde during the inflammation in vitro models [70].

A previous study by Sanghvi et al (2019) reported that the knockdown of FN3K could promote the downregulation of Nrf2 target proteins in murine MYC/sgKeap1 HCC liver tumor isografts and similarly in three pairs of FN3K-proficient and FN3K-deficient human xenografts (Huh1, H460, and H3255) models [4]. Nrf2 inhibitors can induce the chemosensitization of chemotherapeutic molecules in HCC [71]. **Brusatol** is a potent Nrf2 inhibitor that can induce impairment of melanoma growth through the regulation of Akt-Nrf2 signaling when given along with UV irradiation [72]. **Topotecan** is a semisynthetic anticancer drug and is a derivative of camptothecin which can impair the topoisomerase I activity and induce double-strand DNA breaks. A recent study by Birandra K. Sinha et al 2020 reported the underlying mechanisms of topotecan-induced cancer cell death by examining the gene expression through microarray analysis. The study concluded that the topotecan treatment onto MCF-7 cells mitigated the expression of P53-regulating genes, estrogen receptor alpha (ERα/ESR1), and antiapoptotic genes (Bcl2) [73]. In addition, enhanced expression patterns of ‘ROS-related sensor genes [73] and proapoptotic genes in ZR-75-1 cell lines could be the most significant factors implicated in mediated topotecan-induced cancer cell death [73].

In this study, we have examined the activity of brusatol and topotecan cytotoxicity activity against breast cancers BT-474 and T47D invasive type breast cancers. We selected several natural, synthetic kinase inhibitors and screened the most potent multikinase inhibitors such as sorafenib, neratinib, tamoxifen citrate, and gefitinib potentially enhanced the deglycating enzymatic protein, FN3K consequently modulated the expression of Nrf2-downstream genes such as Keap1, HO-1, and NQO1. With sorafenib treatment, the expression of Keap1 and FN3K is upregulated whereas tamoxifen citrate also enhanced the expression of FN3K with concomitant modulation in the Nrf2 expression. It has been observed that the substantial expression of FN3K can result in the deglycation of Nrf2 resulting in the overexpression of AREs to enhance the cancer cell antioxidant potential [4].

Treatment of anticancer drugs such as cisplatin [74], oxaliplatin [75], and topotecan [75] can alter the expression of mRNA pertinent to FN3K [https://ctdbase.org/]. So, we have ascertained the IC-50 using cytotoxicity assays for these drugs and treated the invasive ductal breast carcinoma cells to examine the FN3K protein expression. In our study, FN3K expression is substantially suppressed with oxaliplatin during the treatment process in BT-474 cells. However, the expression of Nrf2 and consequently ARE-related gene expression was minimized in ductal invasive carcinomas such as BT-474. Cisplatin did not decrease the FN3K expression in BT-474. Furthermore, the expression patterns of FN3K were upregulated with kinase inhibitors, methotrexate but downregulated with both cisplatin/oxaliplatin in T47D cells. But, brusatol is an Nrf2 inhibitor of natural origin significantly upregulated the FN3K expression in both BT-474 and T4-7D cells (**S3 Fig**) indicating the combinatorial implications of both FN3K and Nrf2 inhibitors in breast cancers by examining Nrf2-glycation patterns.

It has been concluded that the Nrf2 inhibition could impair the cancer cell migration and consequently induce sensitization of cancer cells to chemotherapy [6]. Knockdown of Nrf2 genes could induce A549 sensitization to cisplatin and etoposide. In addition, blocking the activity of Nrf2 by trigonelline can induce susceptibility of pancreatic cancer cells to apoptosis. In our study, the NQO1 activity declined with the treatment of sorafenib, tamoxifen citrate, neratinib, DMF, gefitinib, cisplatin, oxaliplatin, and although its activity enhanced with cyclosporine A but consequently mitigated in a dose-dependent manner with the treatment of cyclosporine A. Patterns of NQO1 activity was declined with methotrexate treatment than control. Our study reported the expression of FN3K in ductal carcinomas, which are invasive types. A previous study depicted the knockdown of FN3K could induce a higher glycated Nrf2 in the cancer cells [4]. Therefore, the development of small molecule inhibitors is a significant strategy to inhibit selectively FN3K in breast cancers. Substantial studies are required to examine the activity of Nrf2 inhibitors/semisynthetic camptothecin derivatives like irinotecan, and topotecan along with FN3K inhibitors (oxaliplatin) in combinatorial regimens to target several cancers.

## Conclusion

Screening of several kinase inhibitors, and anticancer drugs by molecular docking and molecular dynamic simulations was executed and subsequently examined the screened molecules for their ADME properties and the FN3K protein expression patterns. The current study demonstrated that oxaliplatin significantly downregulated the FN3K protein expression, a deglycating enzyme involved in Nrf2 glycation, a process implicated in tumor progression. In addition, the breast cancer cell death was substantially higher although the expression of FN3K enhanced with certain kinase inhibitors used for breast cancer treatment and the Nrf2 signalling was modulated consequently NQO1 and HO-1 antioxidant protein expression patterns also declined. It is crucial to examine the combinatorial regimen of natural anticancer molecules/Nrf2 blockers/kinase inhibitors with oxaliplatin to examine glycation patterns of Nrf2. Therefore, the antioxidant responses can be mitigated to alleviate breast cancer progression.

### Summary

- FN3K expression is significantly upregulated when compared to control in all the drug-treated groups such as neratinib, sorafenib, gefitinib, tamoxifen citrate, cyclosporine A, and methotrexate. Consequently, the Nrf2 protein expression was also modulated in neratinib, sorafenib, tamoxifen citrate, and gefitinib, but no changes of expression were observed in the case of cyclosporine A or methotrexate. Keap1 expression patterns were significantly altered in all the drug-treated groups compared to the control.
- However, oxaliplatin completely downregulated FN3K expression in all the treated concentrations when compared to cisplatin in BT-474, and T-47D. Nrf2 and Keap1 expression patterns also altered with these platinum derivatives in BT-474 cells. Other Nrf2 downstream antioxidant genes such as NQO1 and HO-1 were also mitigated in their expression patterns upon drug treatments.
- In the case of T47D cells, both oxaliplatin and cisplatin both downregulated FN3K expression but brusatol (Nrf2 Inhibitor) enhanced the FN3K expression in BT-474, T47D.
- NQO1 activity was declined in sorafenib, tamoxifen citrate, neratinib, DMF, gefitinib, cisplatin, oxaliplatin, and methotrexate, and although its activity enhanced with cyclosporine A in a dose-dependent manner compared to control.

Note: Supplementary figures and tables were attached.

## Supporting information

S1 Fig Supplementary for DMF as a reference (a known FN3K inhibitor) for docking sites onto the AtFN3K protein. (TIF)

S2 Fig Refined chain A MODEL-1 produced from Galaxyweb comparatively considered as the best model for docking and MD simulations. (TIF)

S3 Fig Upregulated expression of FN3K was observed with the treatment of kinase inhibitors including gefitinib, neratinib, sorafenib, cyclosporine A, tamoxifen citrate, and with other anticancer molecules such as topotecan in T47D cell line. Cisplatin/oxaliplatin treatment resulted in the mitigated expression of FN3K in T47D cells. Brusatol is an Nrf2 inhibitor that upregulated the FN3K expression in luminal-type breast cancer cells such as BT-474 and T47D. DMF, a competitive inhibitor of FN3K, was used as a positive control. GAPDH was used as an internal control.
(TIF)

S4 Fig (TIF)

S1 Table HPLC characterization of sorafenib, tamoxifen citrate, neratinib, gefitinib, cisplatin, oxaliplatin, cyclosporine A, methotrexate, topotecan, brusatol—chromatography conditions. (DOCX)

S1 File Original blots-1: The original blots were provided as supporting information files and attached as original blots-1. (PDF)

S2 File Original blots-2: The original blots were provided as supporting information files and attached as original blots-2. (PDF)

S1 Graphical abstract (TIF)
