# Supplementary figures and images for: Screening fructosamine-3-kinase (FN3K) inhibitors, a deglycating enzyme of oncogenic Nrf2: Human FN3K homology modelling, docking and molecular dynamics simulations
Source: PLoS One. 2023 Nov 1;18(11):e0283705. doi: 10.1371/journal.pone.0283705 (PMC10619859; doi:10.1371/journal.pone.0283705)

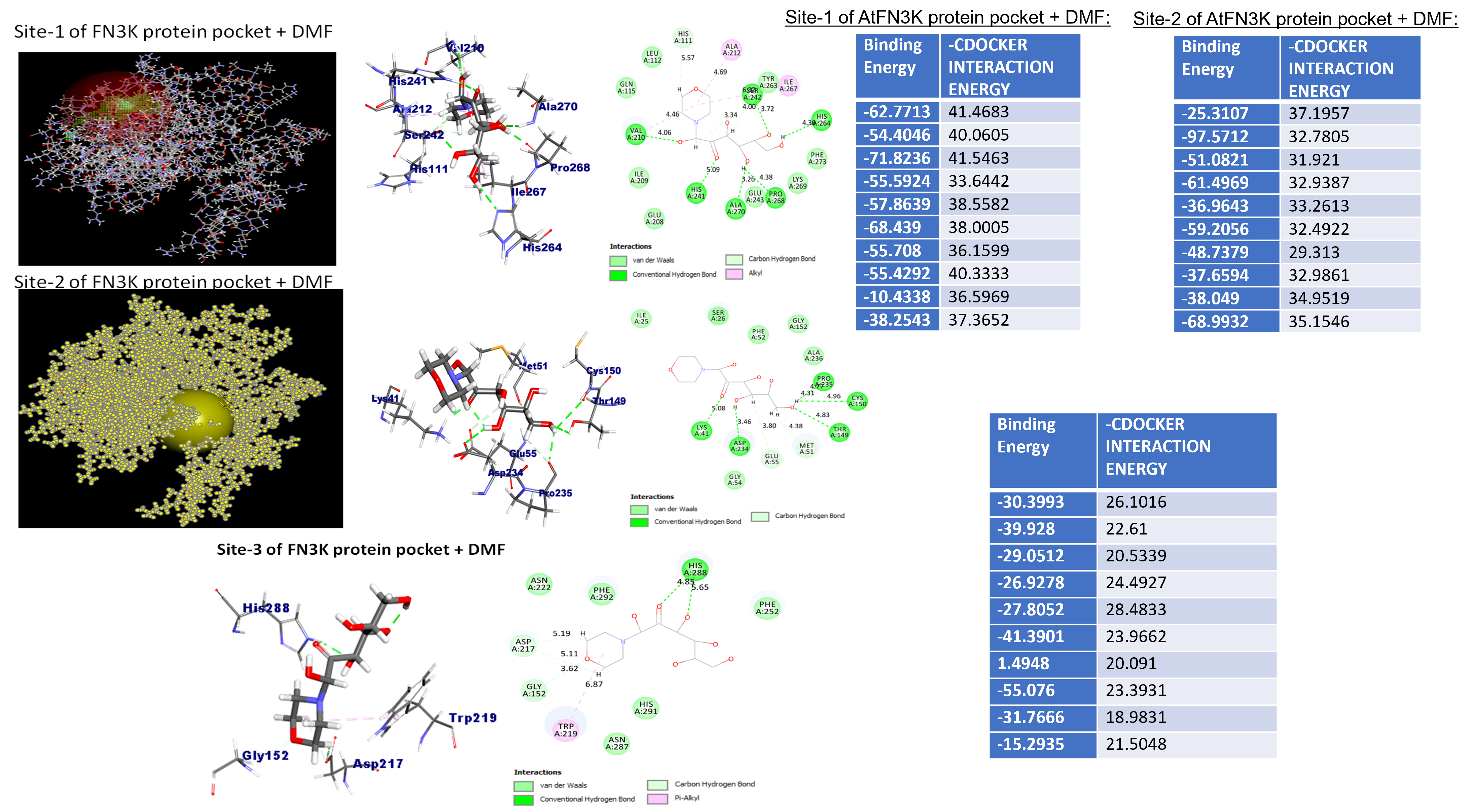

Supplement: S1 Fig — (TIF) [file pone.0283705.s001.tif]

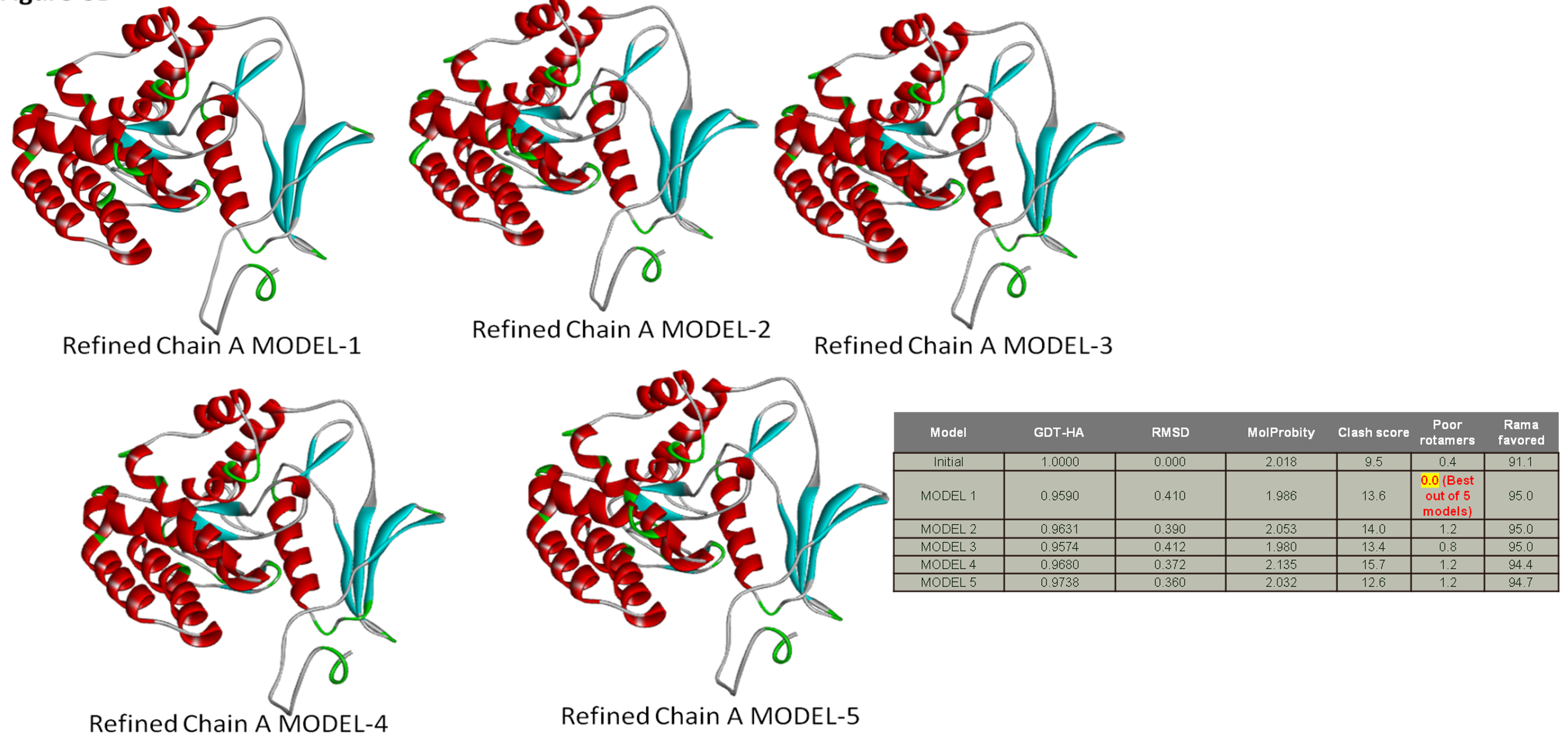

Supplement: S2 Fig — (TIF) [file pone.0283705.s002.tif]

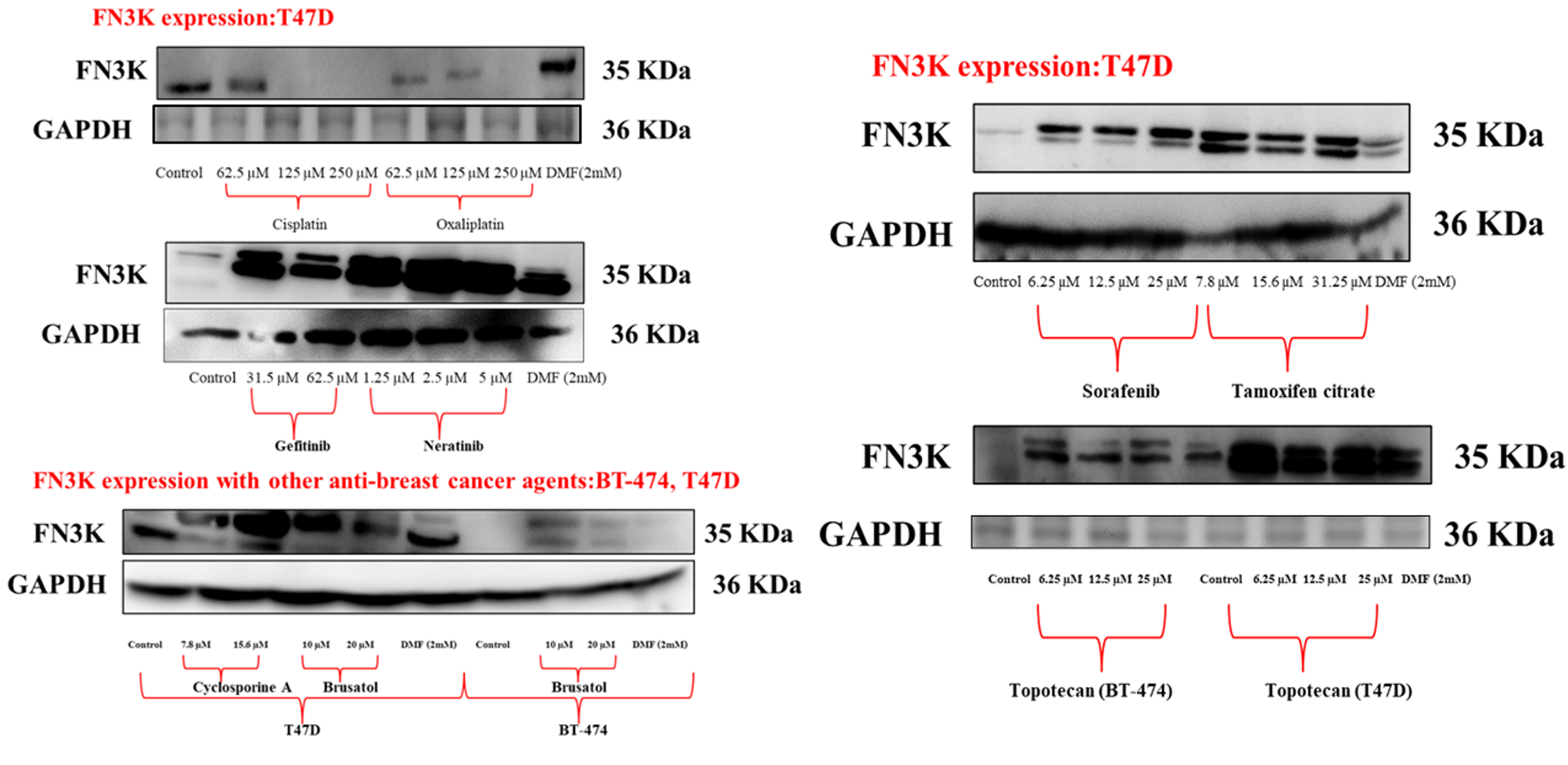

Supplement: S3 Fig — Cisplatin/oxaliplatin treatment resulted in the mitigated expression of FN3K in T47D cells. Brusatol is an Nrf2 inhibitor that upregulated the FN3K expression in luminal-type breast cancer cells such as BT-474 and T47D. DMF, a competitive inhibitor of FN3K, was used as a positive control. GAPDH was used as an internal control. (TIF) [file pone.0283705.s003.tif]

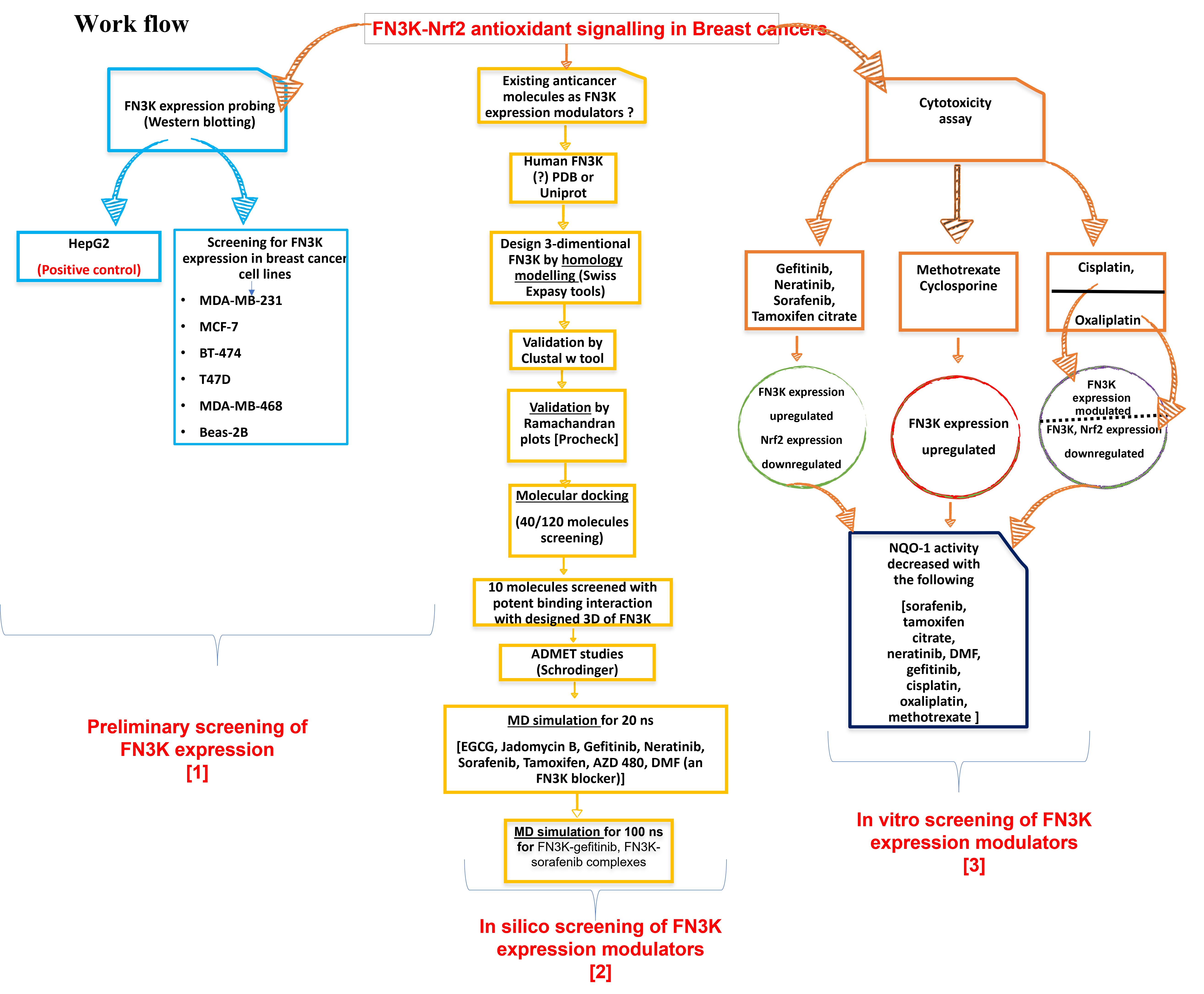

Supplement: S4 Fig — (TIF) [file pone.0283705.s004.tif]

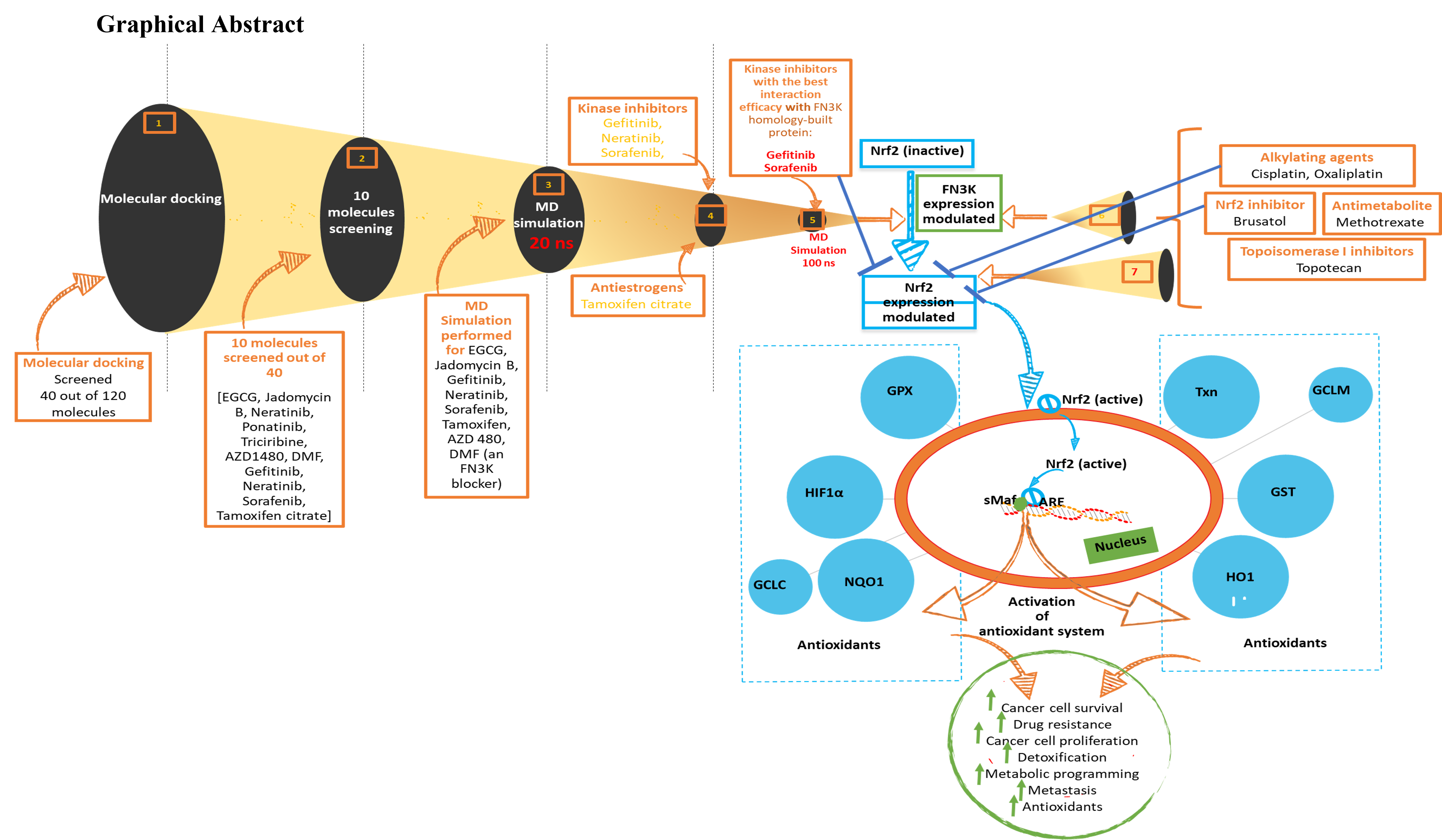

Supplement: S1 Graphical abstract — (TIF) [file pone.0283705.s008.tif]
