# Supplementary material for: Screening fructosamine-3-kinase (FN3K) inhibitors, a deglycating enzyme of oncogenic Nrf2: Human FN3K homology modelling, docking and molecular dynamics simulations
Source: PLoS One. 2023 Nov 1;18(11):e0283705. doi: 10.1371/journal.pone.0283705 (PMC10619859; doi:10.1371/journal.pone.0283705)
Supplement: S1 Table — (DOCX) [file pone.0283705.s005.docx]

**S1 Table.** HPLC characterization of sorafenib, tamoxifen citrate, neratinib, gefitinib, cisplatin, oxaliplatin, cyclosporine A, methotrexate, topotecan, brusatol - chromatography conditions**.**

Sorafenib

| **Final optimized HPLC Conditions for** Sorafenib | |
| --- | --- |
| Column | Kromasil C18(25 cmx 4.6 mm,5 µm); |
| Pump mode | Isocratic |
| Wavelength | 260 nm |
| Injection volume | 20 µL |
| Flow rate | 1.0 mL/minute |
| Column Temperature | 30˚C |
| Mobile phase | acetonitrile and 20 mmol/L ammonium acetate in a proportion 53:47 (vol/vol) |
| Runtime | 10 minutes. |
| Sample concentration | 10 µg/ml |

Gefitinib

| **Final optimized HPLC Conditions for** Gefitinib | |
| --- | --- |
| Column | Inertsil C8 (25 cmx 4.6 mm,5 µm); |
| Pump mode | Isocratic |
| Wavelength | 246 nm |
| Injection volume | 20 µL |
| Flow rate | 1.0mL/minute |
| Column Temperature | 50˚C |
| Mobile phase | 50 mM aqueous ammonium acetate: acetonitrile as the mobile phase |
| Runtime | 12 minutes. |
| Sample concentration | 10µg/ml |

Neratinib

| **Final optimized HPLC Conditions for** Neratinib | |
| --- | --- |
| Column | Altima column (150 mm × 4.6 mm × 5μ) |
| Pump mode | Isocratic |
| Wavelength | 215 nm |
| Injection volume | 10 µL |
| Flow rate | 1.0mL/minute |
| Column Temperature | 35˚C |
| Mobile phase | (monopotassium phosphate) and acetonitrile (60:40 v/v) with 0.1% formic acid |
| Runtime | 10minutes. |
| Sample concentration | 10 µg/ml |

Tamoxifen citrate

| **Final optimized HPLC Conditions for** Tamoxifen citrate | |
| --- | --- |
| Column | Phenomenex C8 (250 cm X 4.6 mm) |
| Pump mode | Isocratic |
| Wavelength | 265 nm |
| Injection volume | 10 µL |
| Flow rate | 0.8mL/minute |
| Column Temperature | 35˚C |
| Mobile phase | methanol: Milli-Q Plus system (Millipore) water: triethyl amine 90:10:0.1% |
| Runtime | 10minutes. |
| Sample concentration | 10 µg/ml |

Chromatographic conditions for topotecan

| **Final optimized HPLC Conditions for topotecan** | |
| --- | --- |
| Column | C18(25 cmx 4.6 mm,5 µm); |
| Pump mode | gradient |
| Wavelength | 267 nm |
| Injection volume | 10 µL |
| Flowrate | 0.8mL/minutes |
| Column Temperature | 35˚C |
| Mobile phase | A(0.05% Formic acid):B(Acetonitrile) 50:50 |
| Runtime | 8 minutes |
| Sample concentration | 10µg |

Chromatographic conditions for Cyclosporine A

| **Final optimized HPLC Conditions for Cyclosporine A** | |
| --- | --- |
| Column | C8(15 cmx 4.6 mm,5 µm); |
| Pump mode | gradient |
| Wavelength | 215 nm |
| Injection volume | 20 µL |
| Flowrate | 1mL/minutes |
| Column Temperature | 45˚C |
| Mobile phase | A(0.3% trifluoracetic acid):B(Acetonitrile) 60:40 |
| Runtime | 10 minutes |
| Sample concentration | 10µg |

Chromatographic conditions for Brusatol

| **Final optimized HPLC Conditions for brusatol** | |
| --- | --- |
| Column | Cyano(25 cmx 4.6 mm,5 µm); |
| Pump mode | gradient |
| Wavelength | 254 nm |
| Injection volume | 10 µL |
| Flowrate | 0.7mL/minutes |
| Column Temperature | 40˚C |
| Mobile phase | A(0.5% Formic acid):B(methanol) 50:50 |
| Runtime | 10 minutes |
| Sample concentration | 20µg |

Chromatographic conditions for Methotrexate

| **Final optimized HPLCConditions for methotrexate** | |
| --- | --- |
| Column | C18(25 cmx 4.6 mm,5 µm); |
| Pump mode | gradient |
| Wavelength | 267 nm |
| Injection volume | 10 µL |
| Flowrate | 1mL/minutes |
| Column Temperature | 50˚C |
| Mobile phase | A(phosphate buffer):B(Acetonitrile) 50:50 |
| Runtime | 10 minutes |
| Sample concentration | 10µg |

Chromatographic conditions for Oxaliplatin

| **Final optimized HPLC Conditions for oxaliplatin** | |
| --- | --- |
| Column | C8(15 cmx 4.6 mm,5 µm); |
| Pump mode | gradient |
| Wavelength | 238 nm |
| Injection volume | 10 µL |
| Flowrate | 0.8mL/minutes |
| Column Temperature | 35˚C |
| Mobile phase | A (acetate buffer):B(Acetonitrile) 30:70 |
| Runtime | 10 minutes |
| Sample concentration | 10µg |

Chromatographic conditions for Cisplatin

| **Final optimized HPLC Conditions for cisplatin** | |
| --- | --- |
| Column | C18(25 cmx 4.6 mm,5 µm); |
| Pump mode | gradient |
| Wavelength | 208 nm |
| Injection volume | 10 µL |
| Flowrate | 1mL/minutes |
| Column Temperature | 35˚C |
| Mobile phase | A(0.1% Formic acid):B(Acetonitrile)C(methanol); 20:50:30 |
| Runtime | 10 minutes |
| Sample concentration | 10µg |
