# Supplementary material for: Screening fructosamine-3-kinase (FN3K) inhibitors, a deglycating enzyme of oncogenic Nrf2: Human FN3K homology modelling, docking and molecular dynamics simulations
Source: PLoS One. 2023 Nov 1;18(11):e0283705. doi: 10.1371/journal.pone.0283705 (PMC10619859; doi:10.1371/journal.pone.0283705)

# Western blots Prior to treatment - FN3K

## FN3K

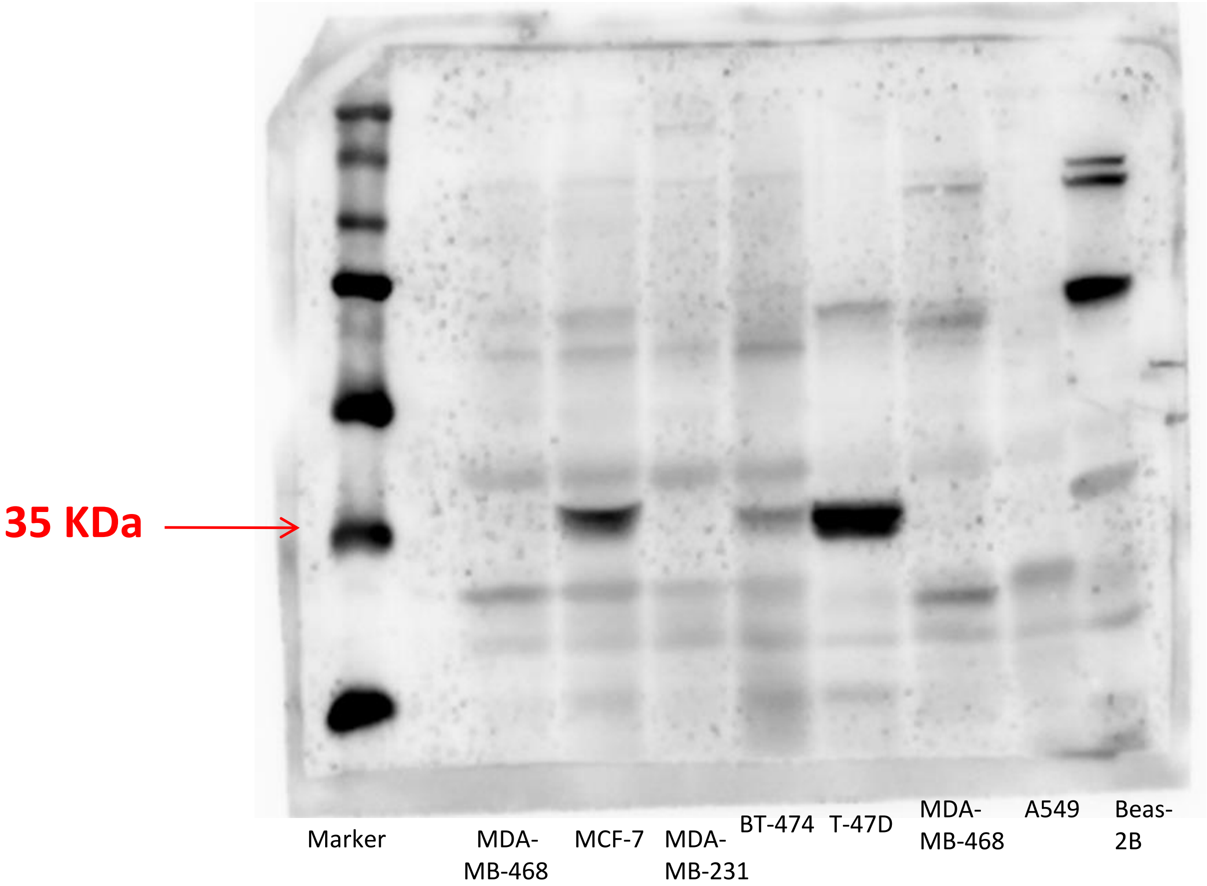

Nrf2

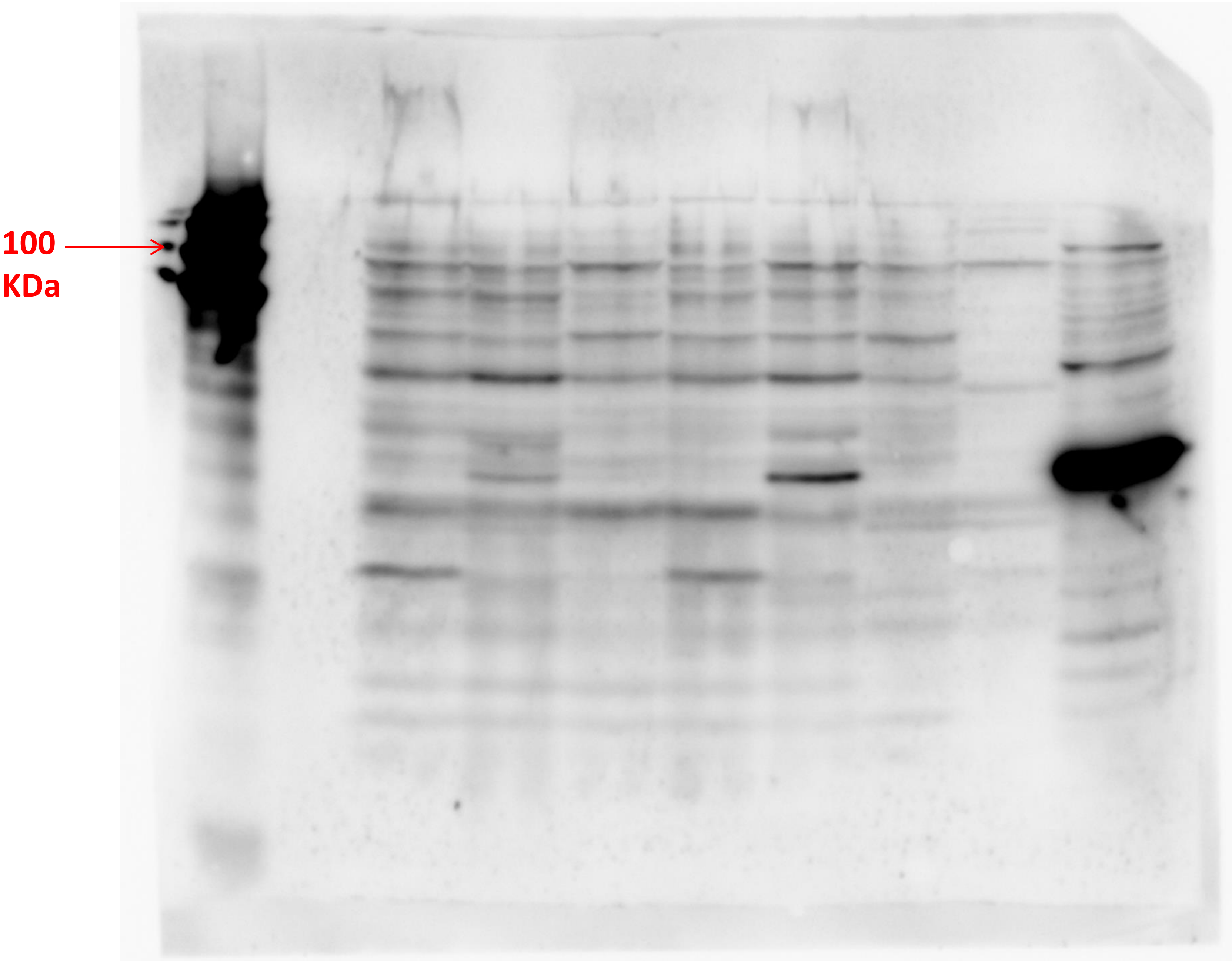

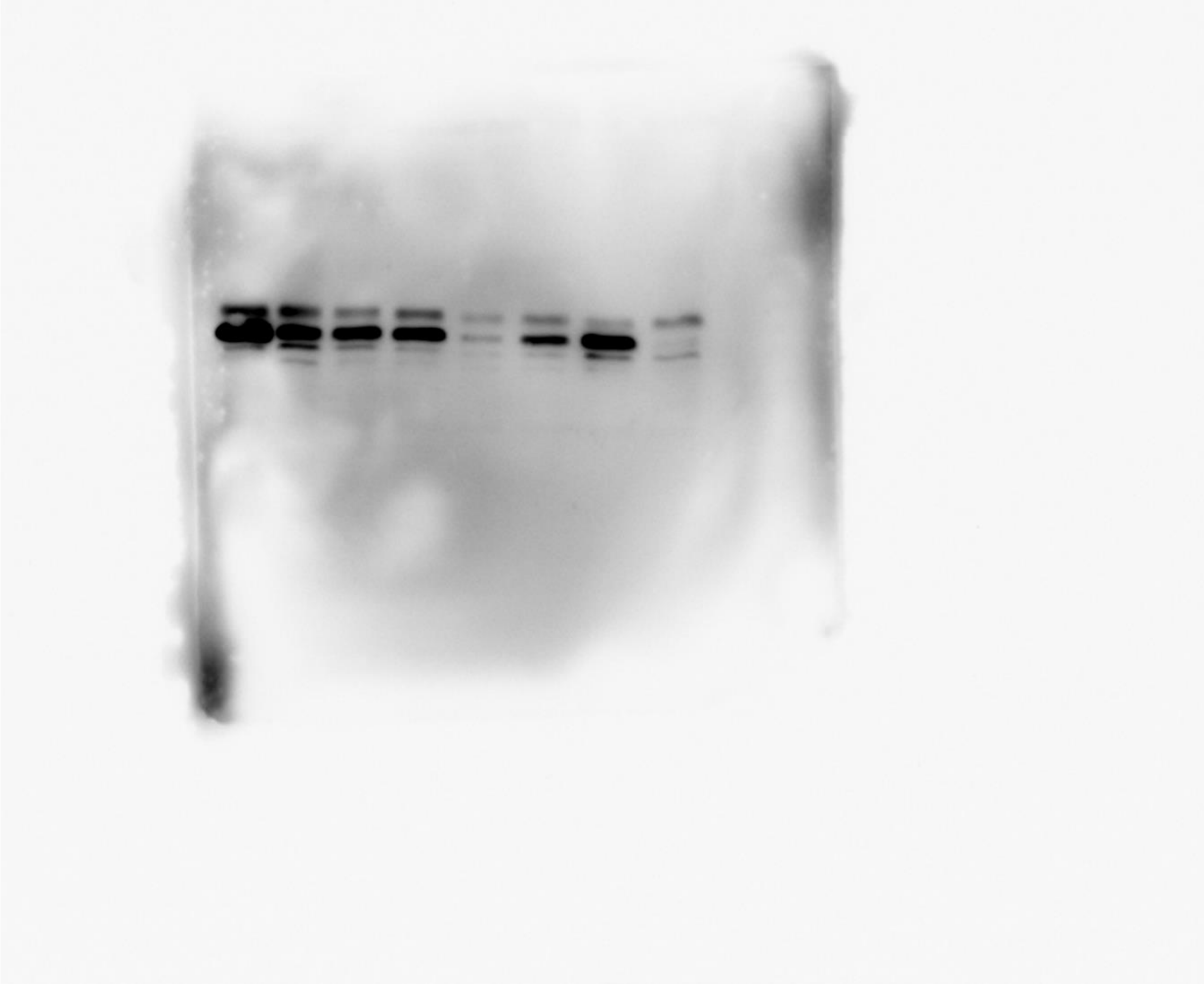

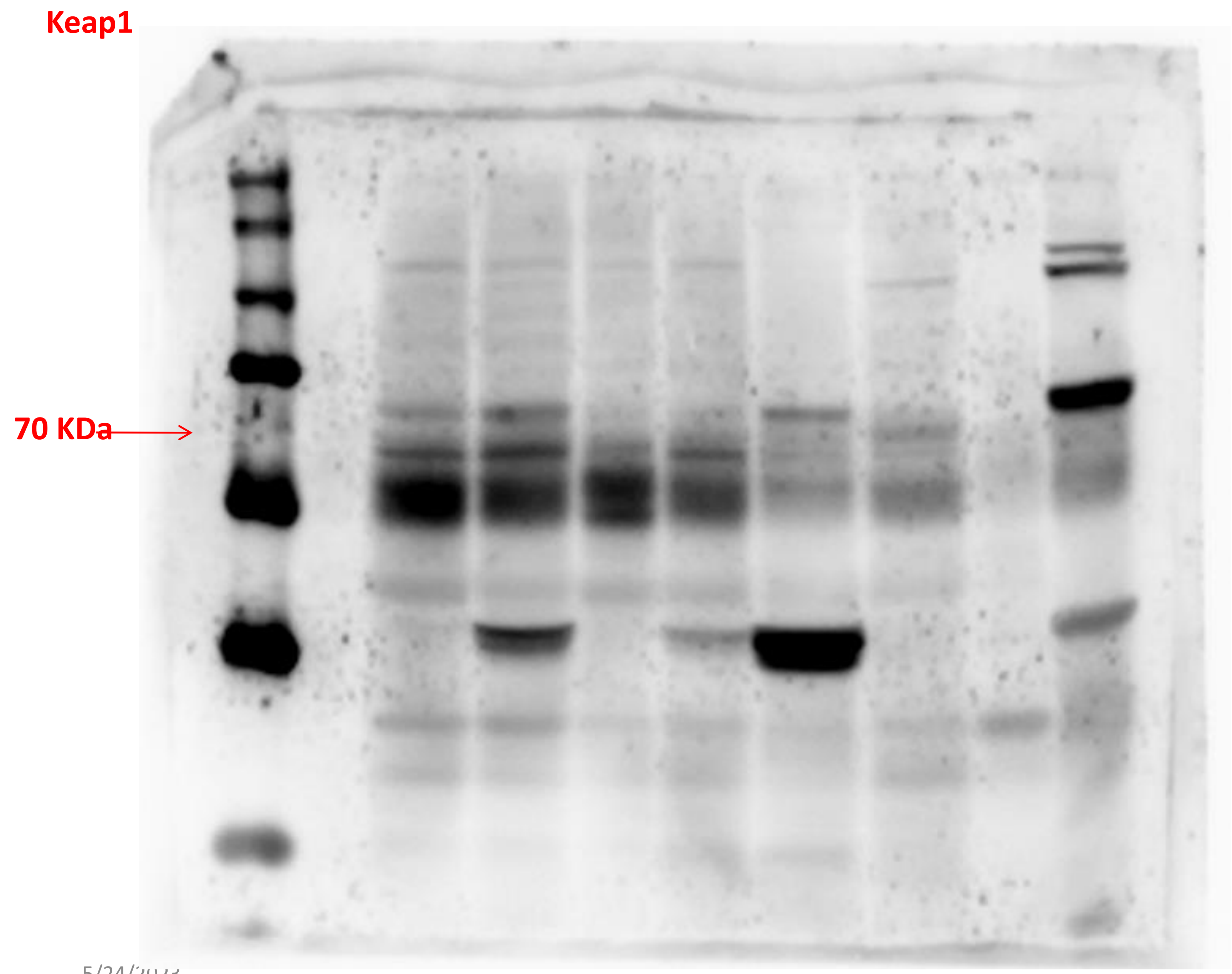

Keap1
70 KDa

GAPDH

36 KDa →

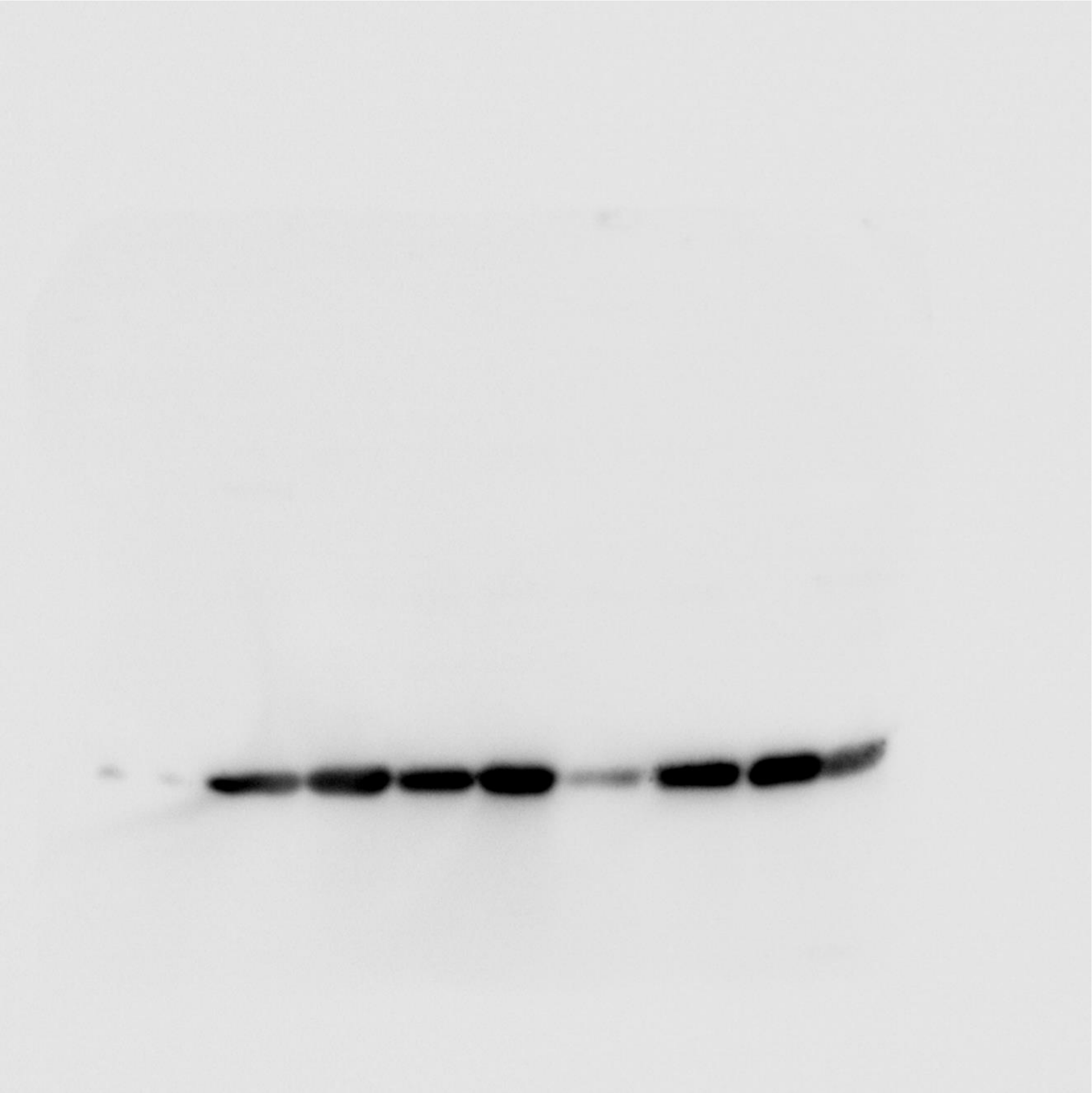

5/24/2023

**Western blots after treatment - FN3K:**
**BT474**
**ductal invasive carcinoma cell line**

Sorafenib

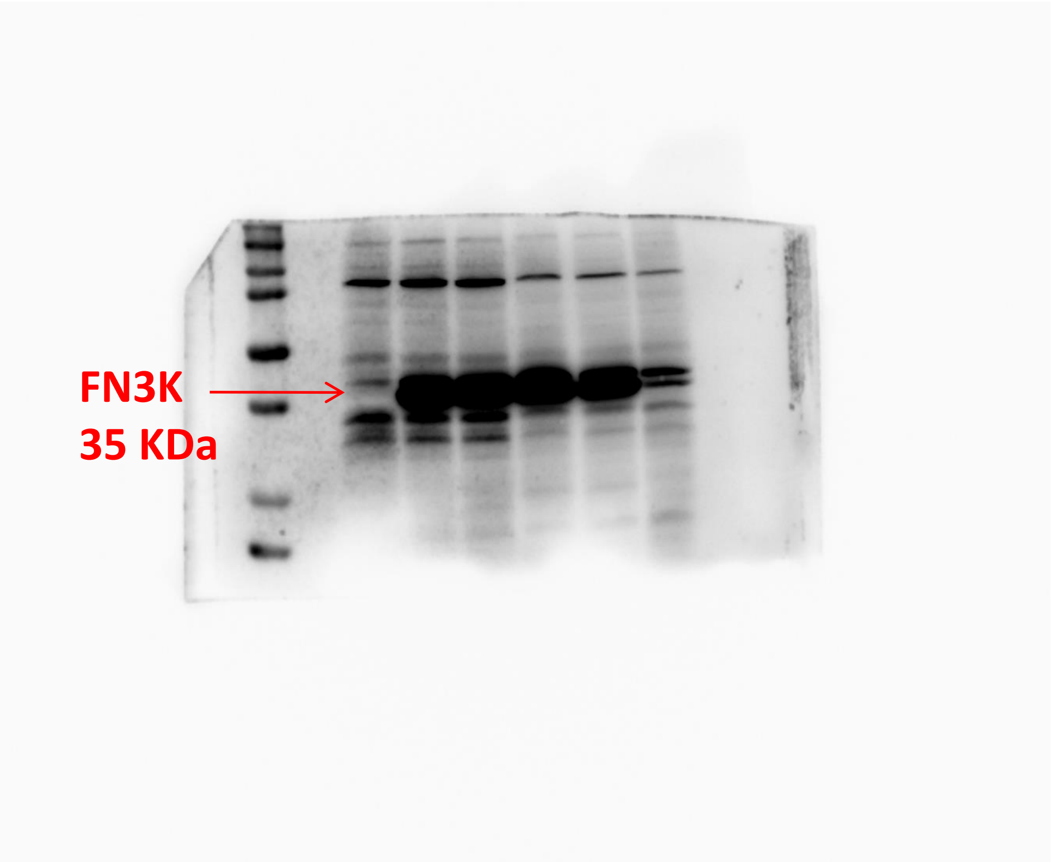
FN3K
35 KDa

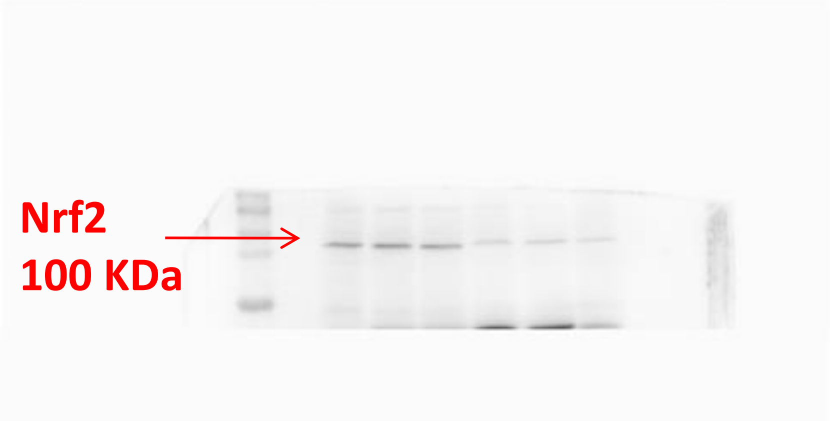
Nrf2
100 KDa

Keap1
70 KDa

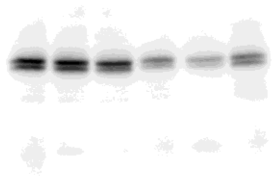

NQO-1
32 KDa

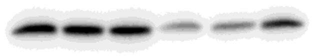

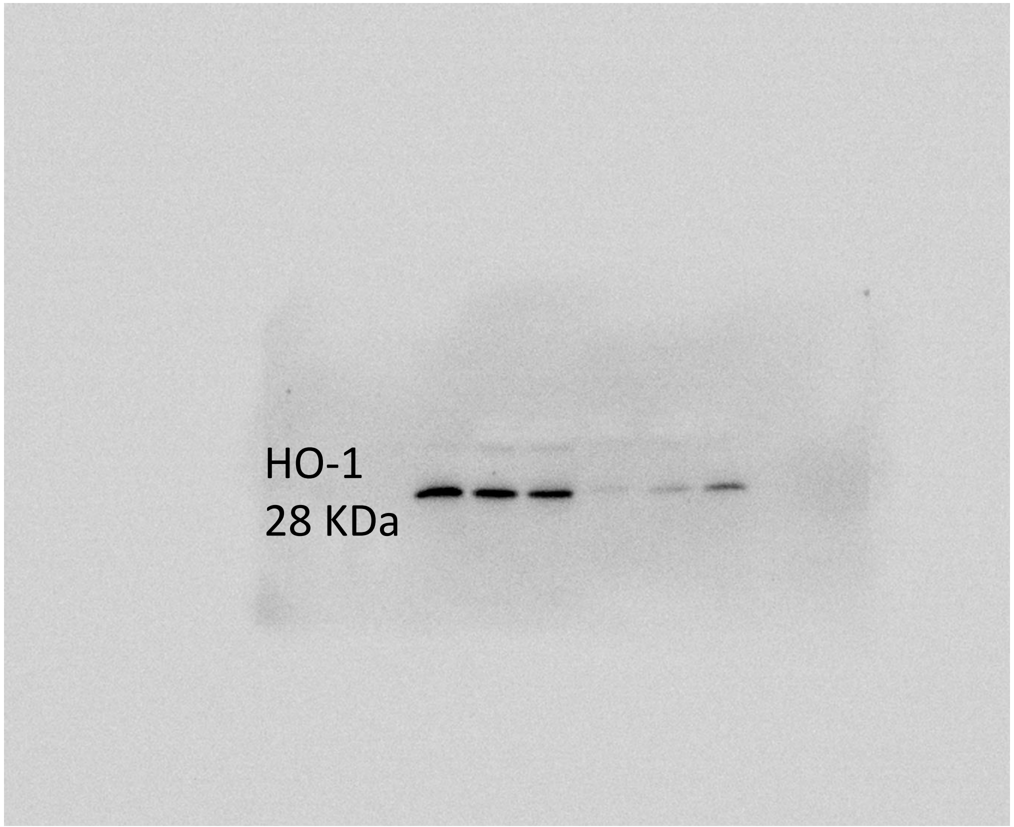
HO-1
28 KDa

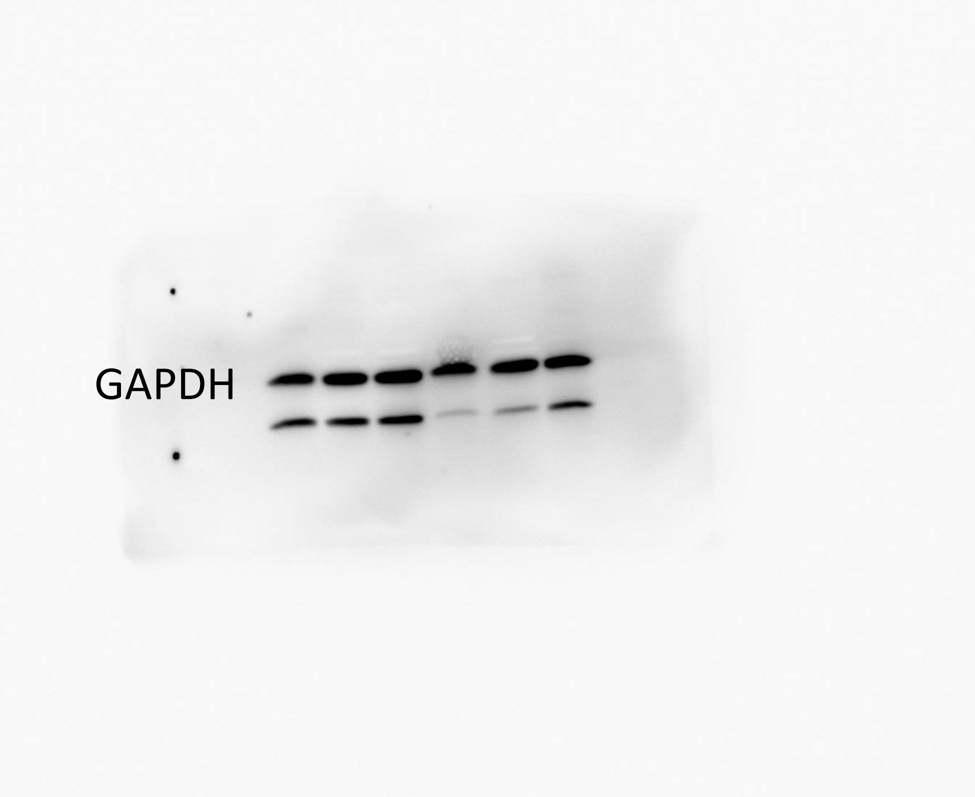
GAPDH

Gefitinib

# FN3K

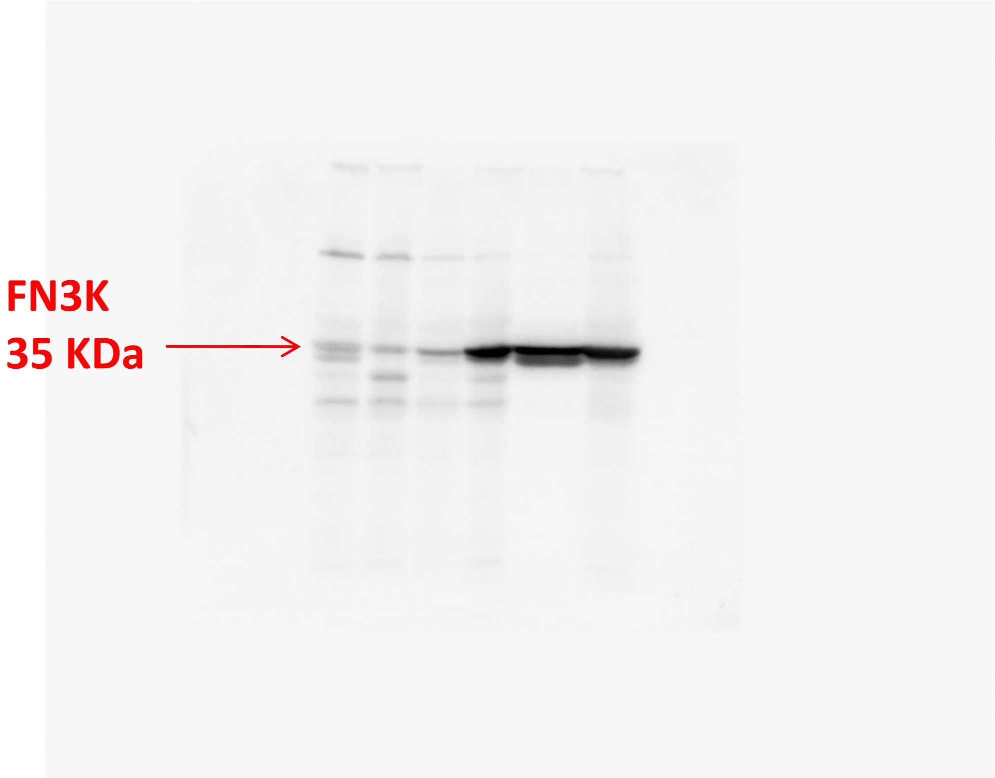

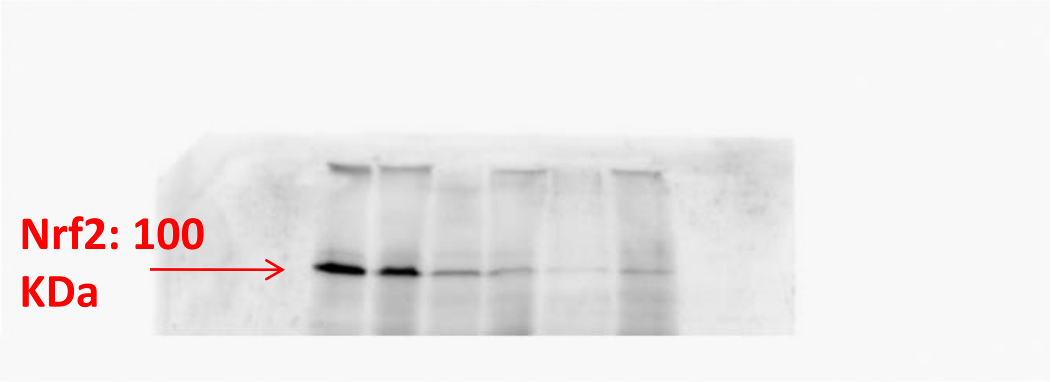
Nrf2: 100
KDa

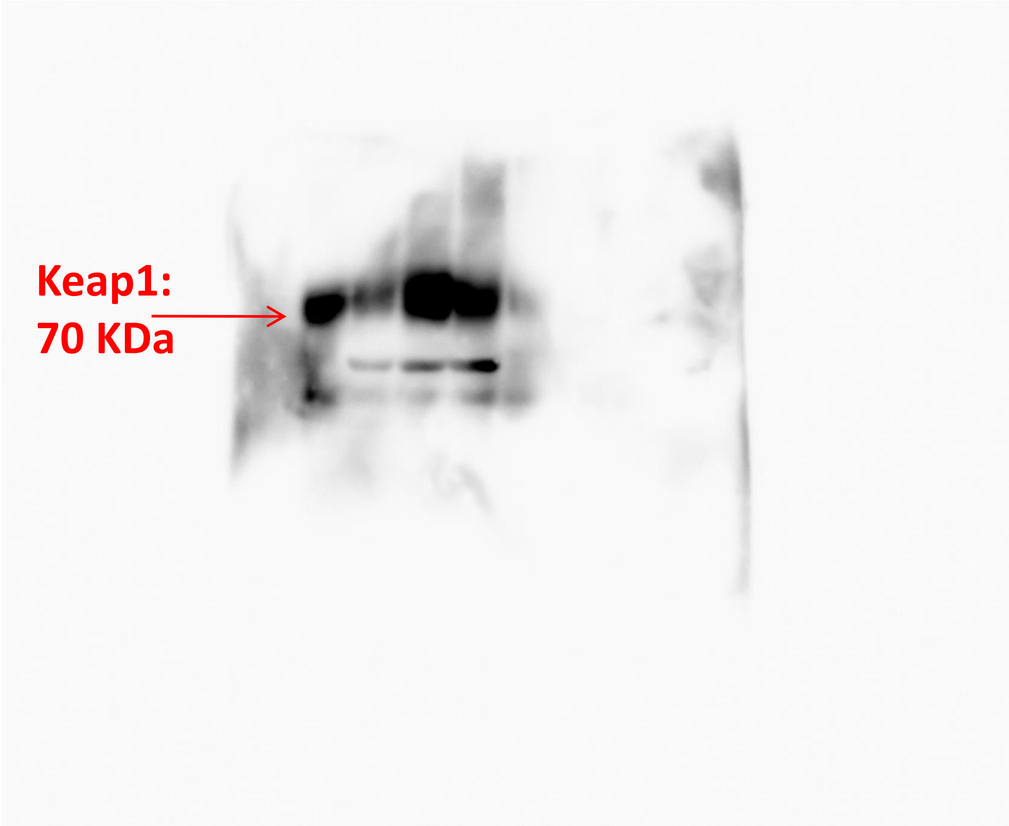
Keap1:
70 KDa

NQO-1

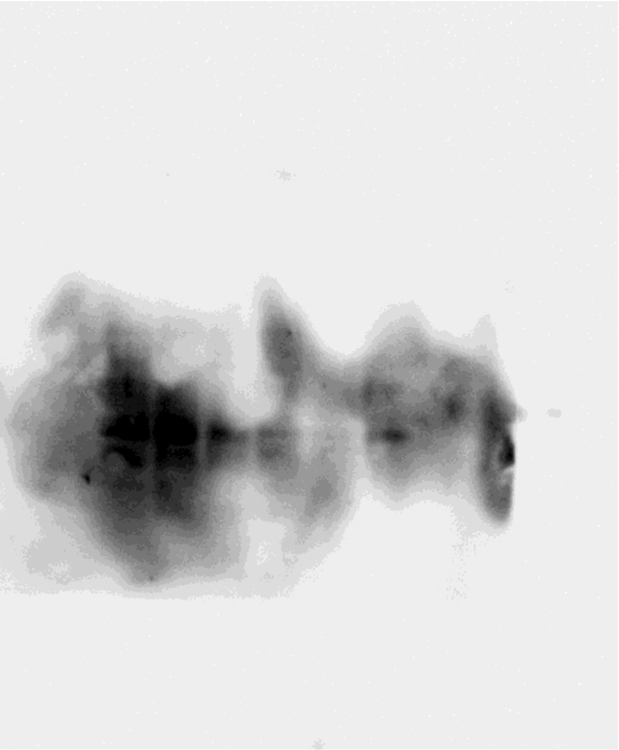

HO-1

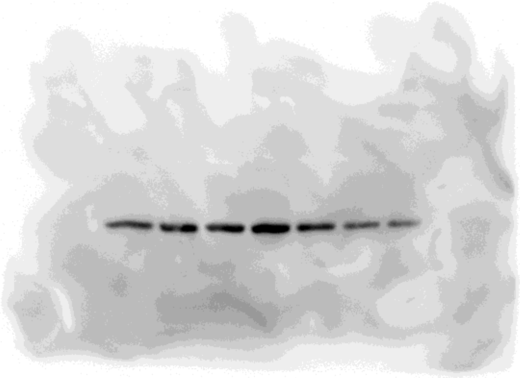

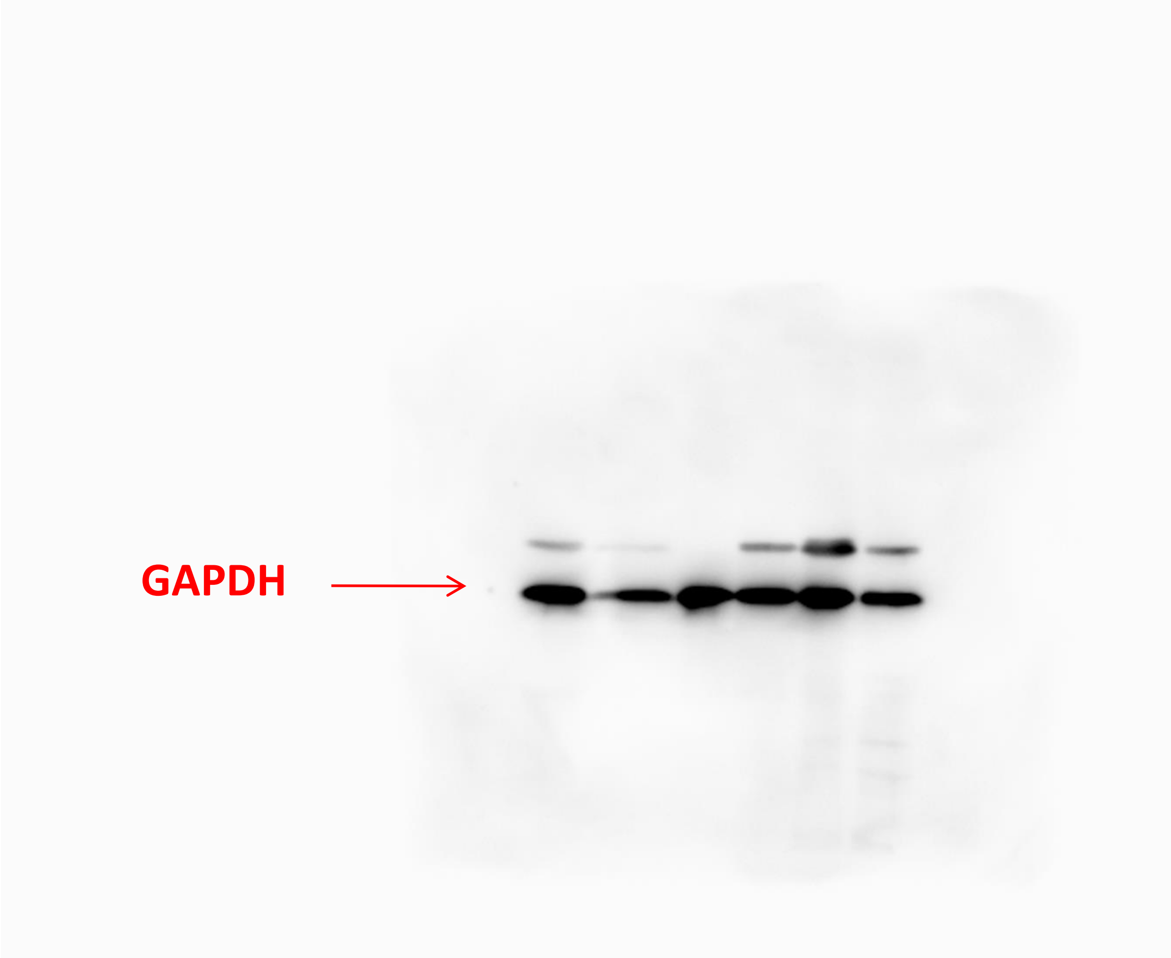
GAPDH

Tamoxifen citrate

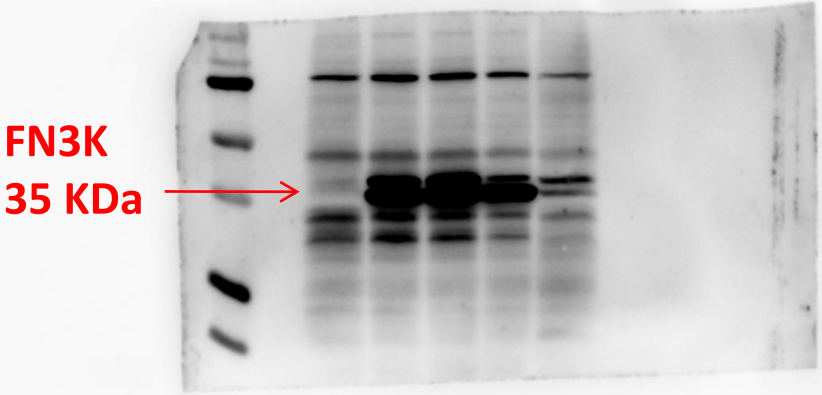
FN3K
35 KDa

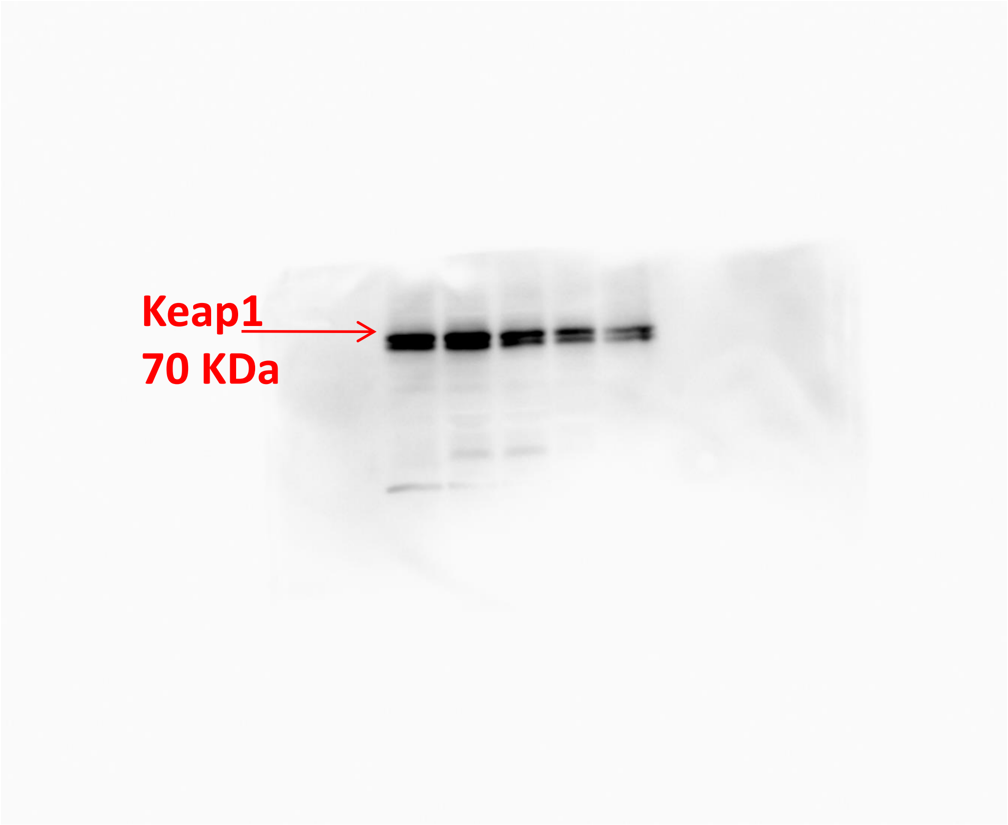
Keap1
70 KDa

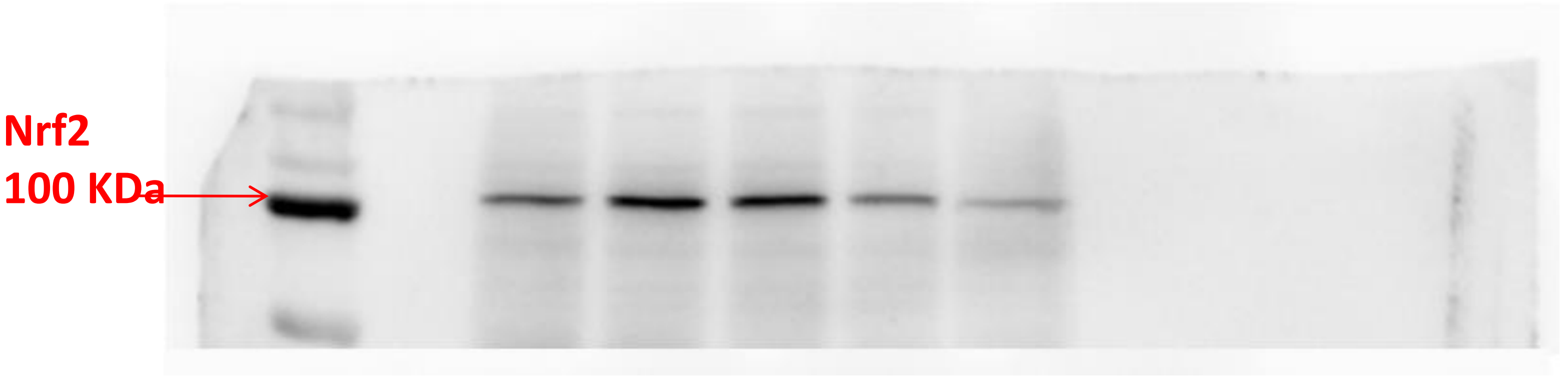
Nrf2
100 KDa

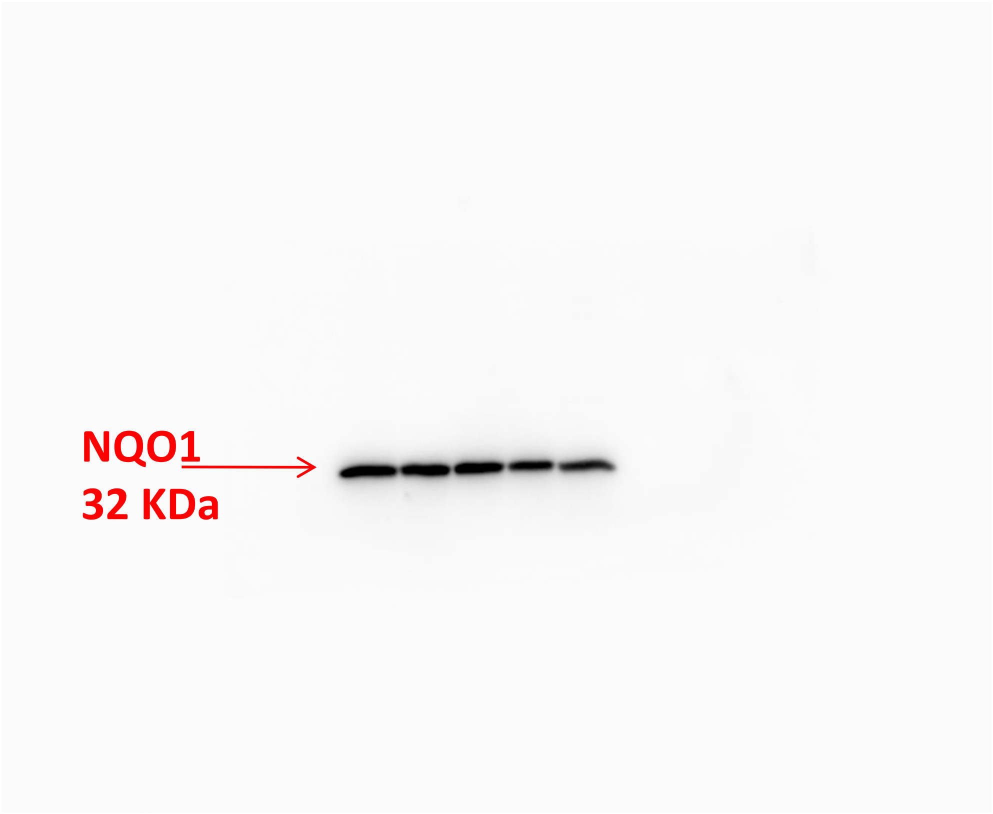
NQO1
32 KDa

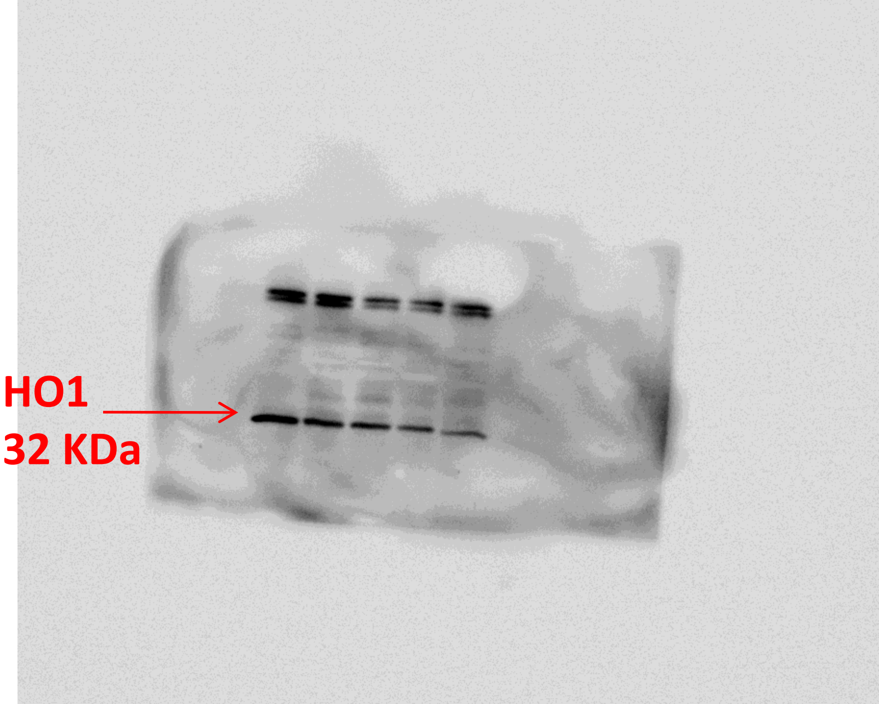
HO1
32 KDa

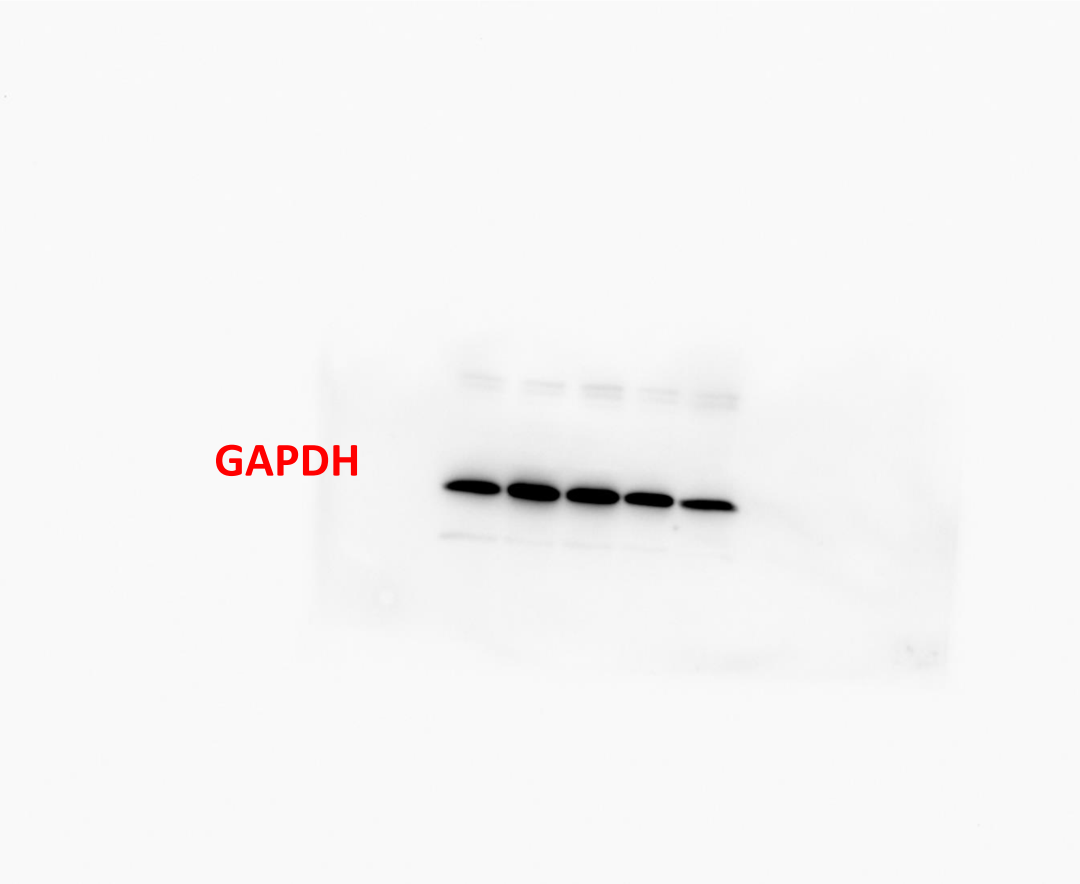
GAPDH

Neratinib

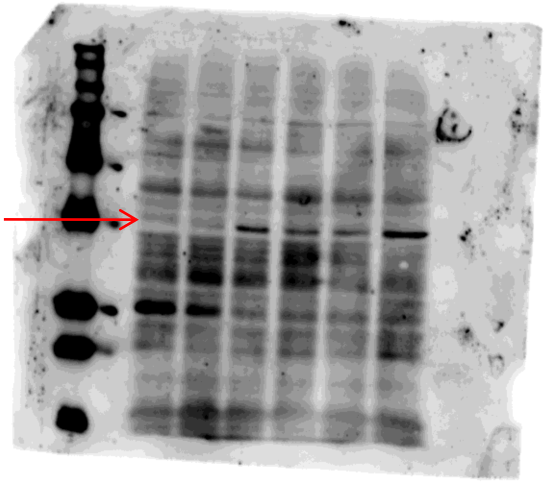
FN3K
35 KDa

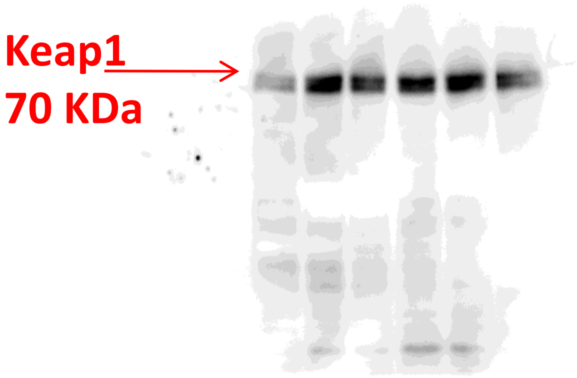
Keap1
70 KDa

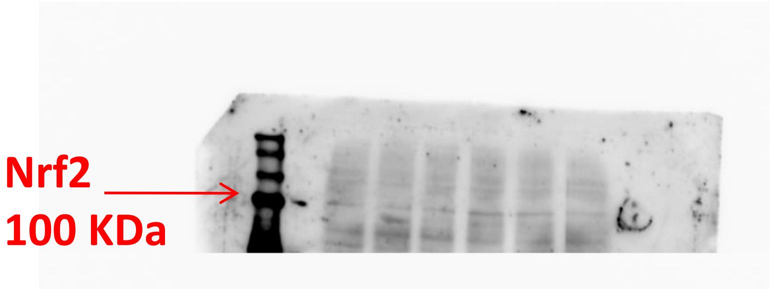
Nrf2
100 KDa

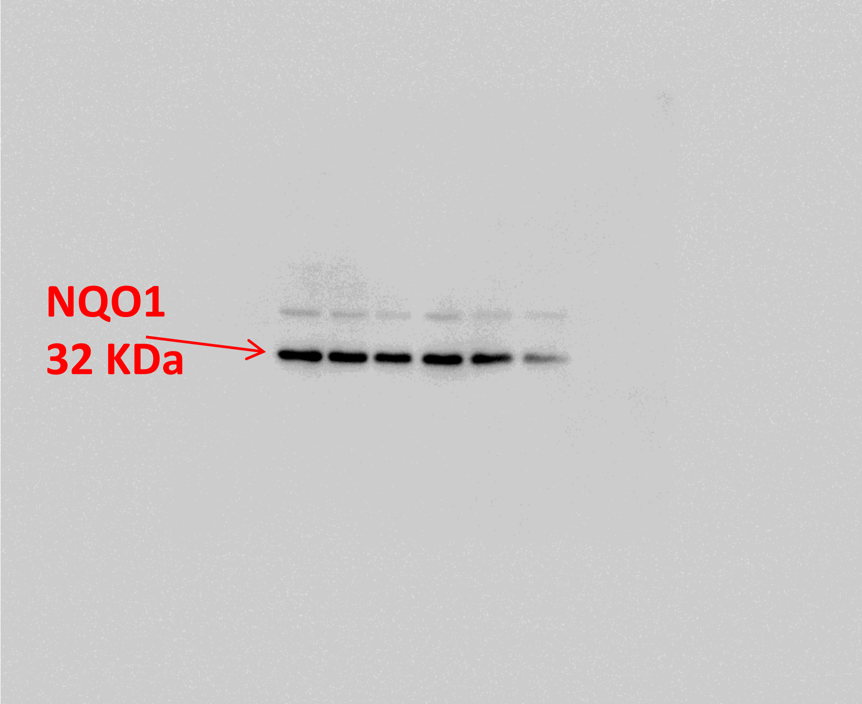
NQO1
32 KDa

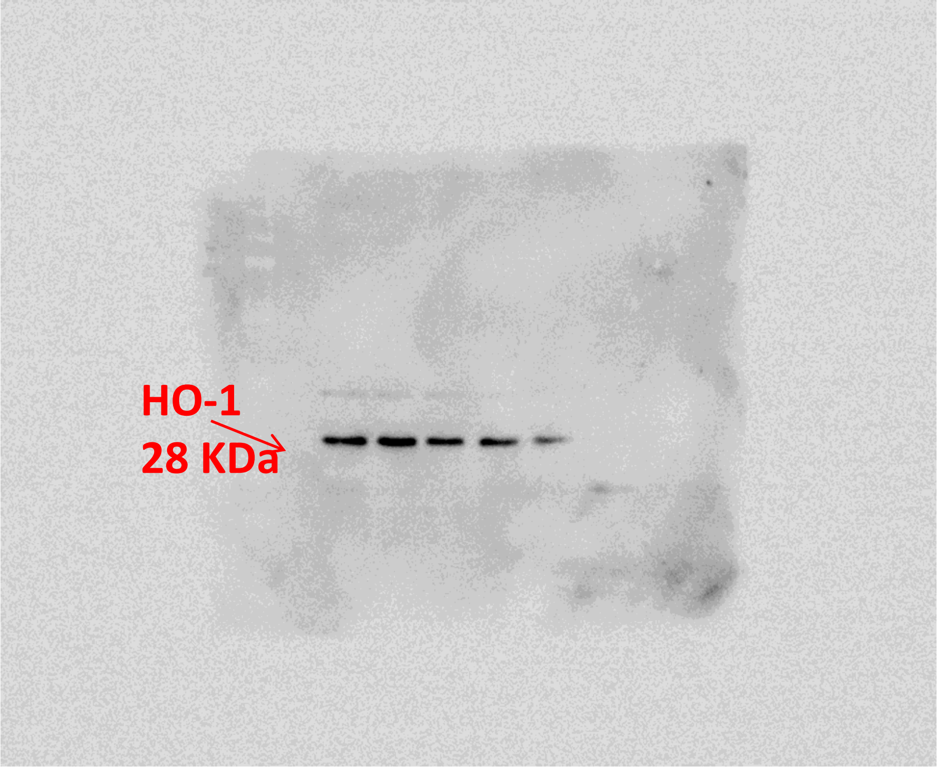
HO-1
28 KDa

GAPDH

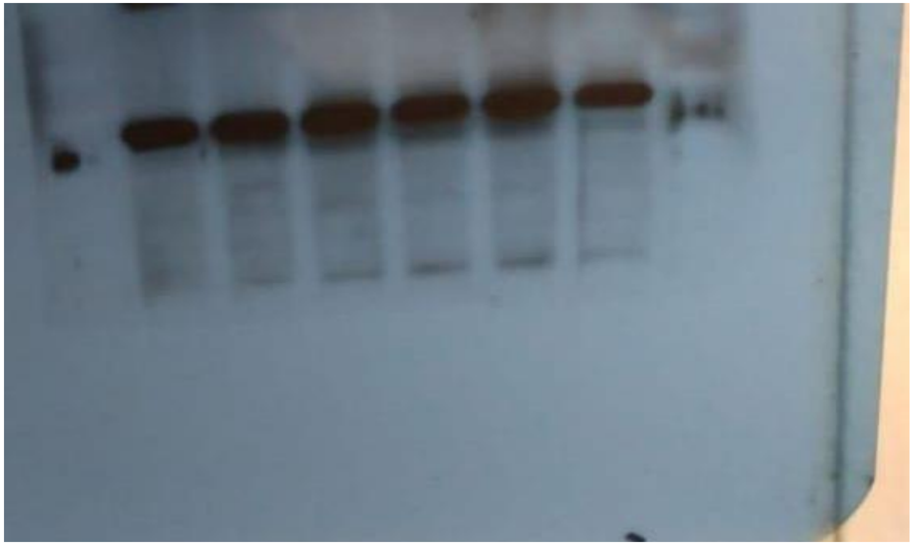

Supplement: S1 File — (PDF) [file pone.0283705.s006.pdf]
