# Supplementary material for: Screening fructosamine-3-kinase (FN3K) inhibitors, a deglycating enzyme of oncogenic Nrf2: Human FN3K homology modelling, docking and molecular dynamics simulations
Source: PLoS One. 2023 Nov 1;18(11):e0283705. doi: 10.1371/journal.pone.0283705 (PMC10619859; doi:10.1371/journal.pone.0283705)

Cyclosporine, Methotrexate

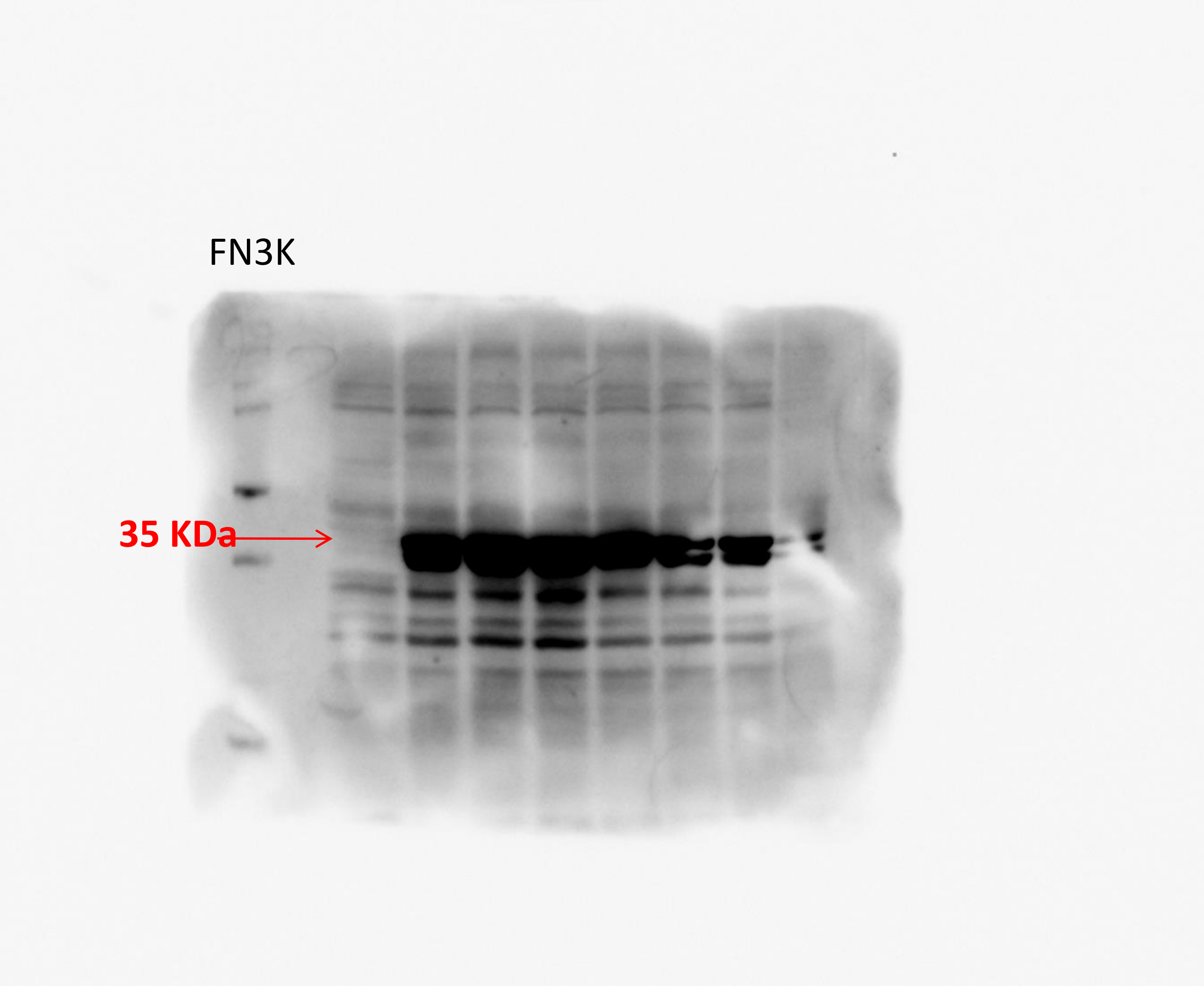
FN3K
35 KDa

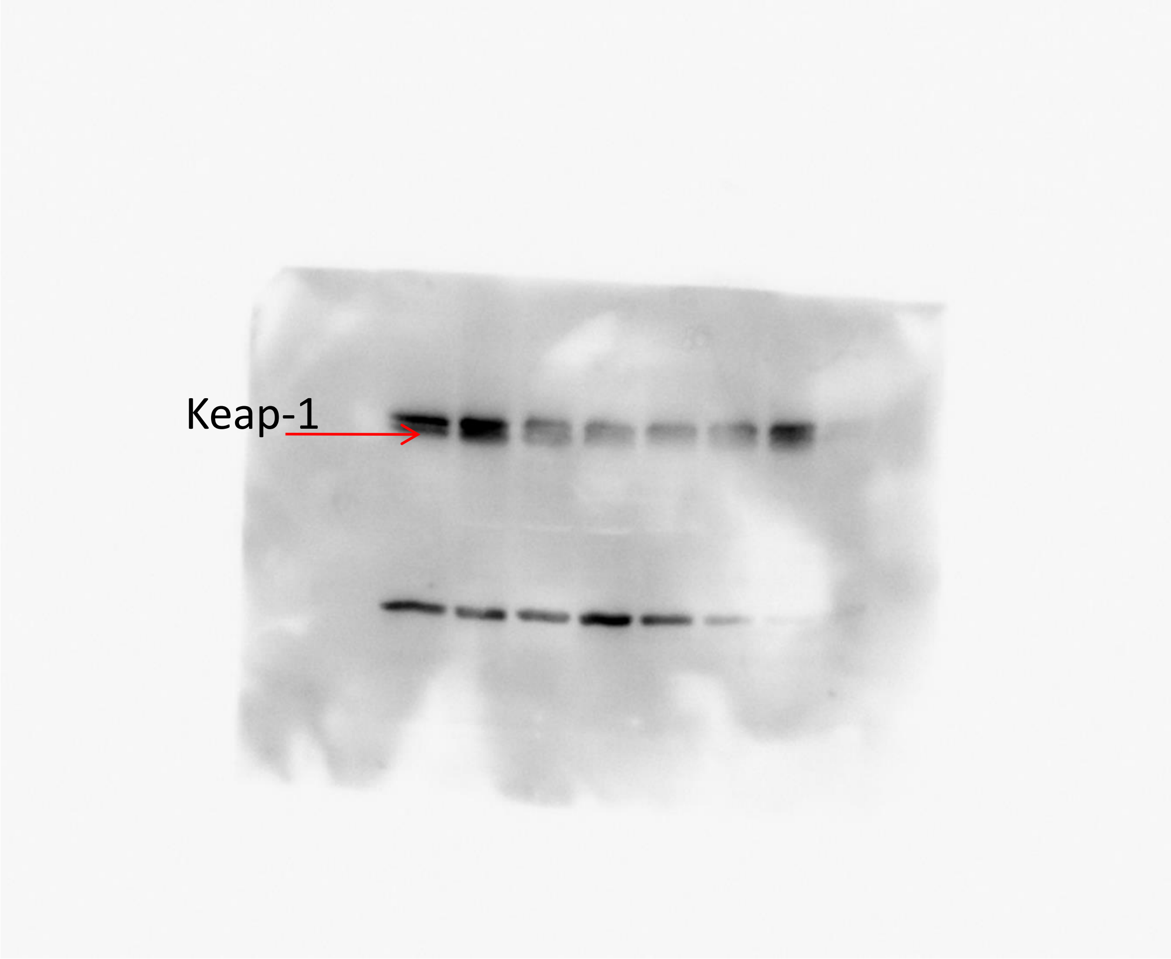
Keap-1

Nrf-2

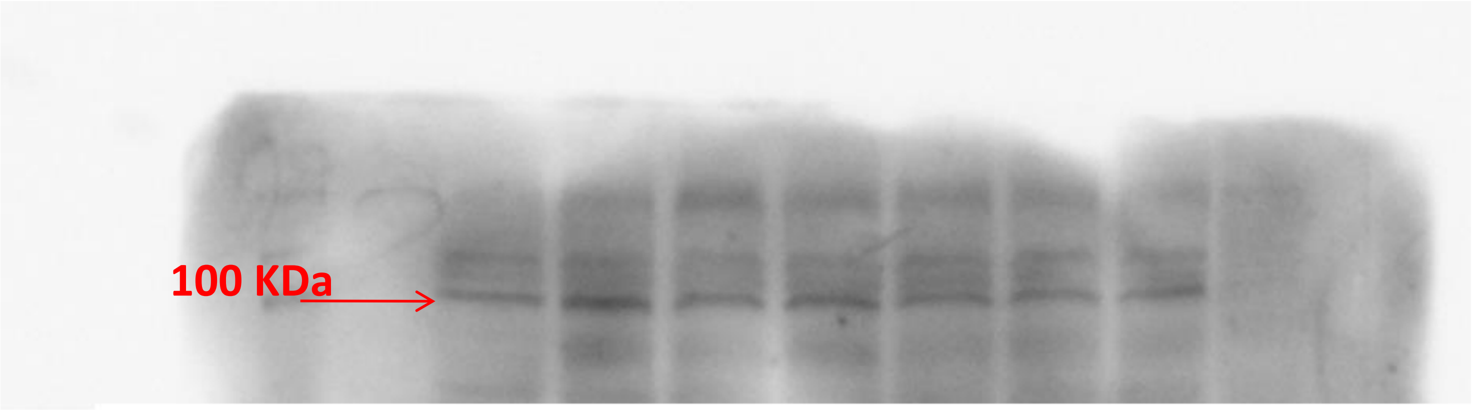

NQO-1

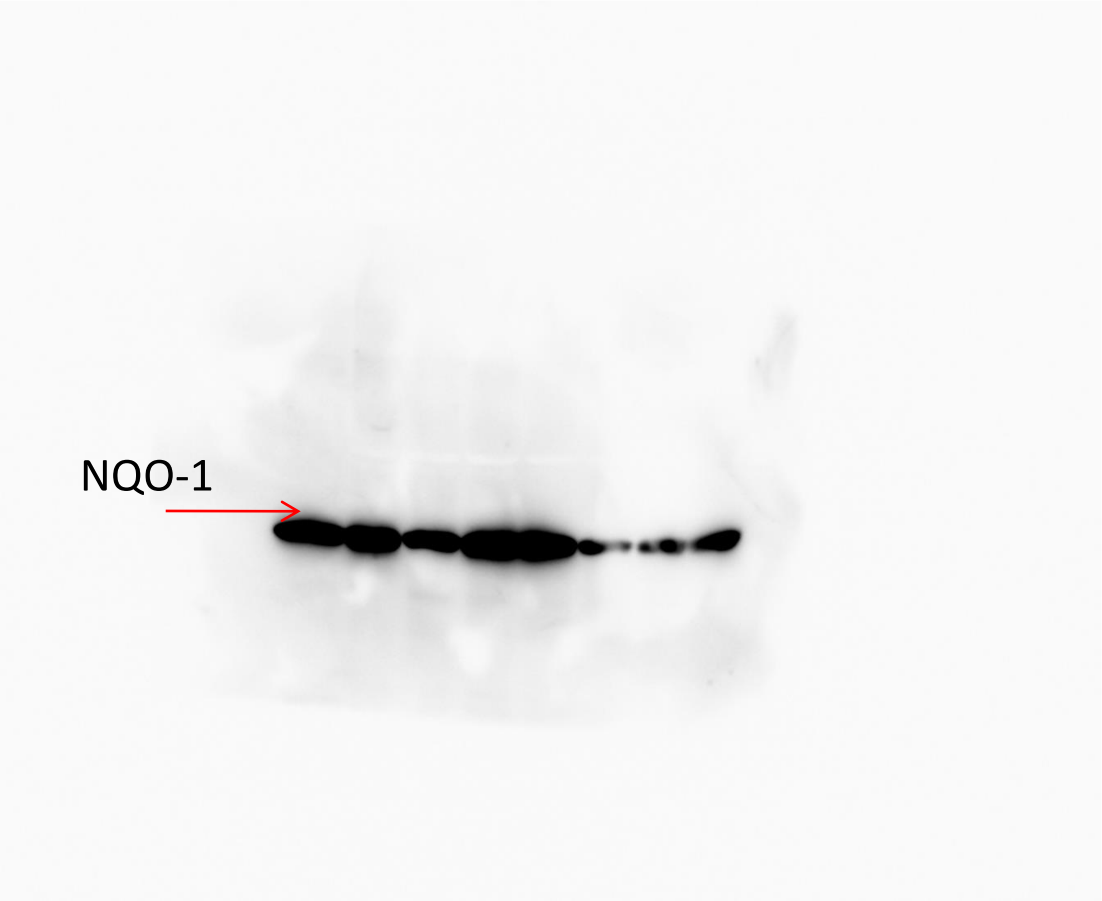

HO-1

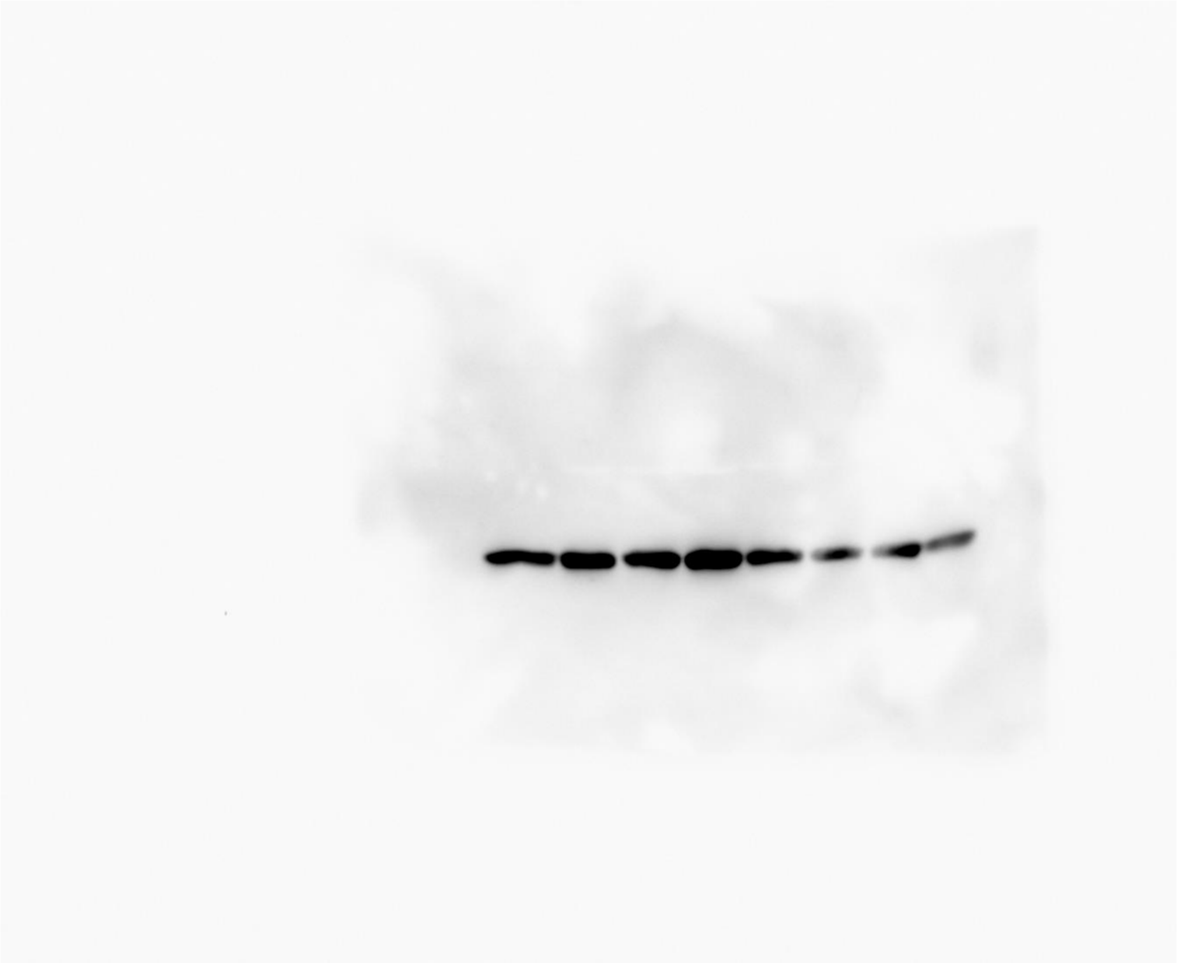

GAPDH

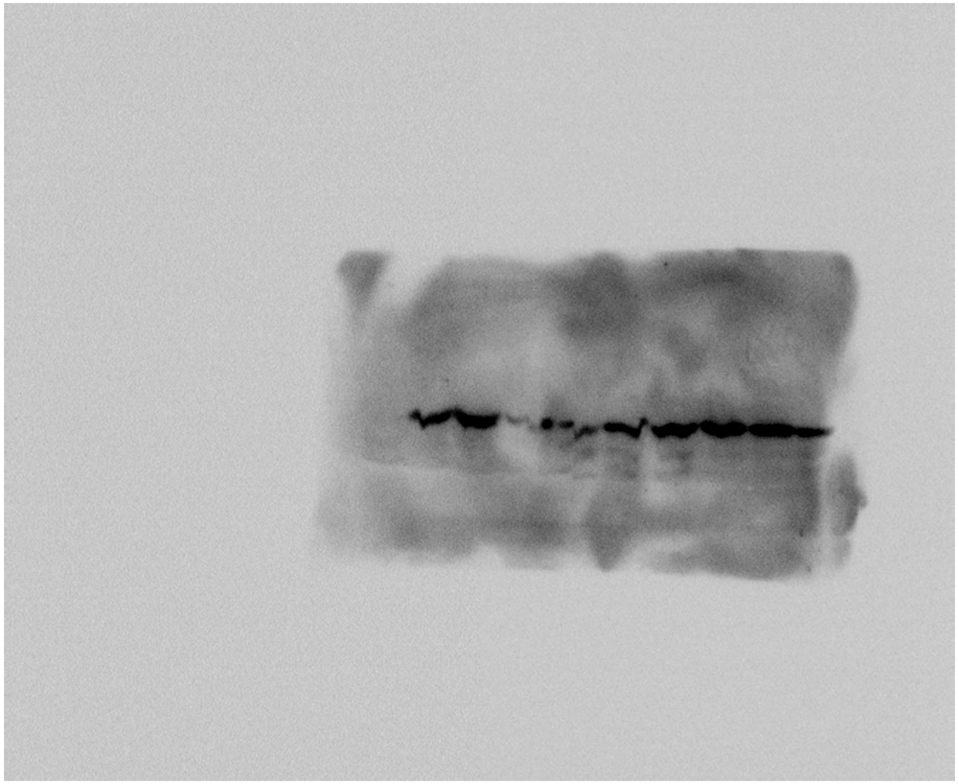

Cisplatin, Oxaliplatin

FN3K

35 KD

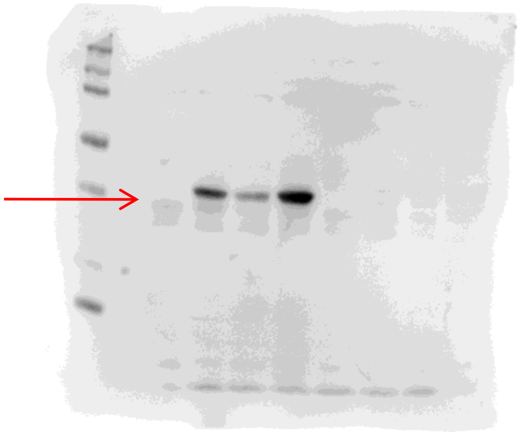

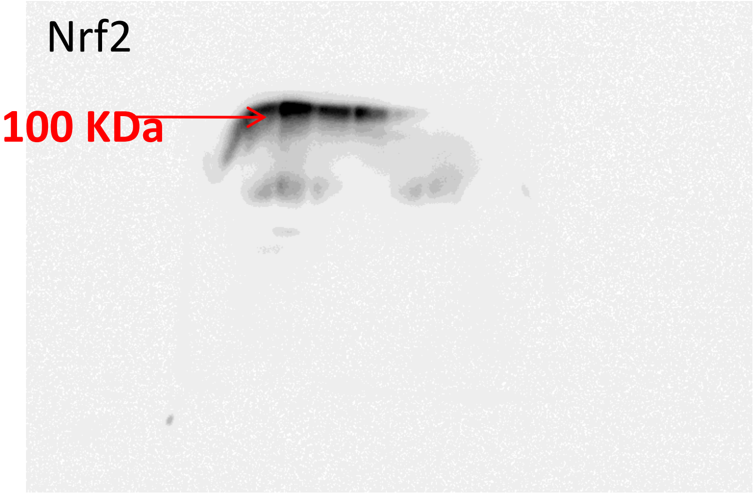
Nrf2
100 KDa

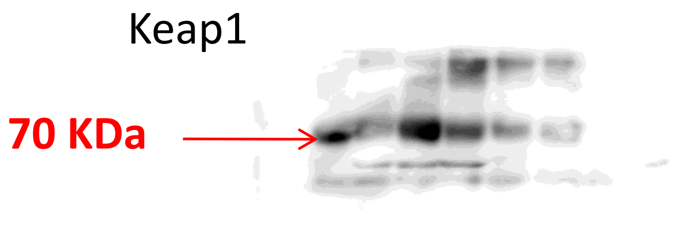

Keap1
70 KDa

NQO-1

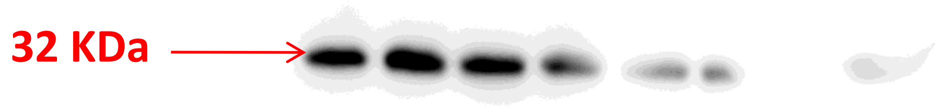

HO-1

28 KDa

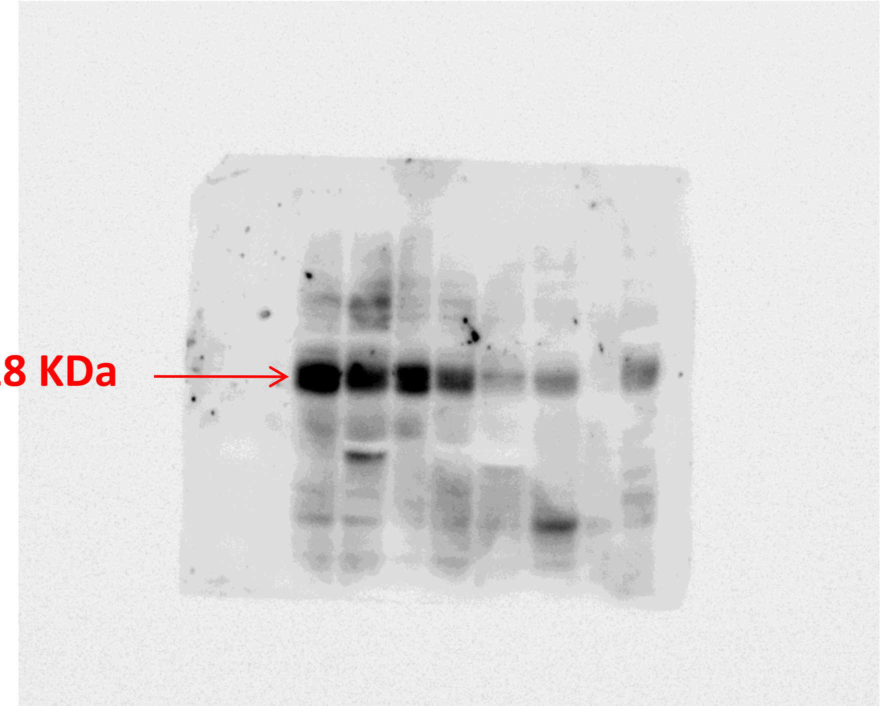

GAPDH

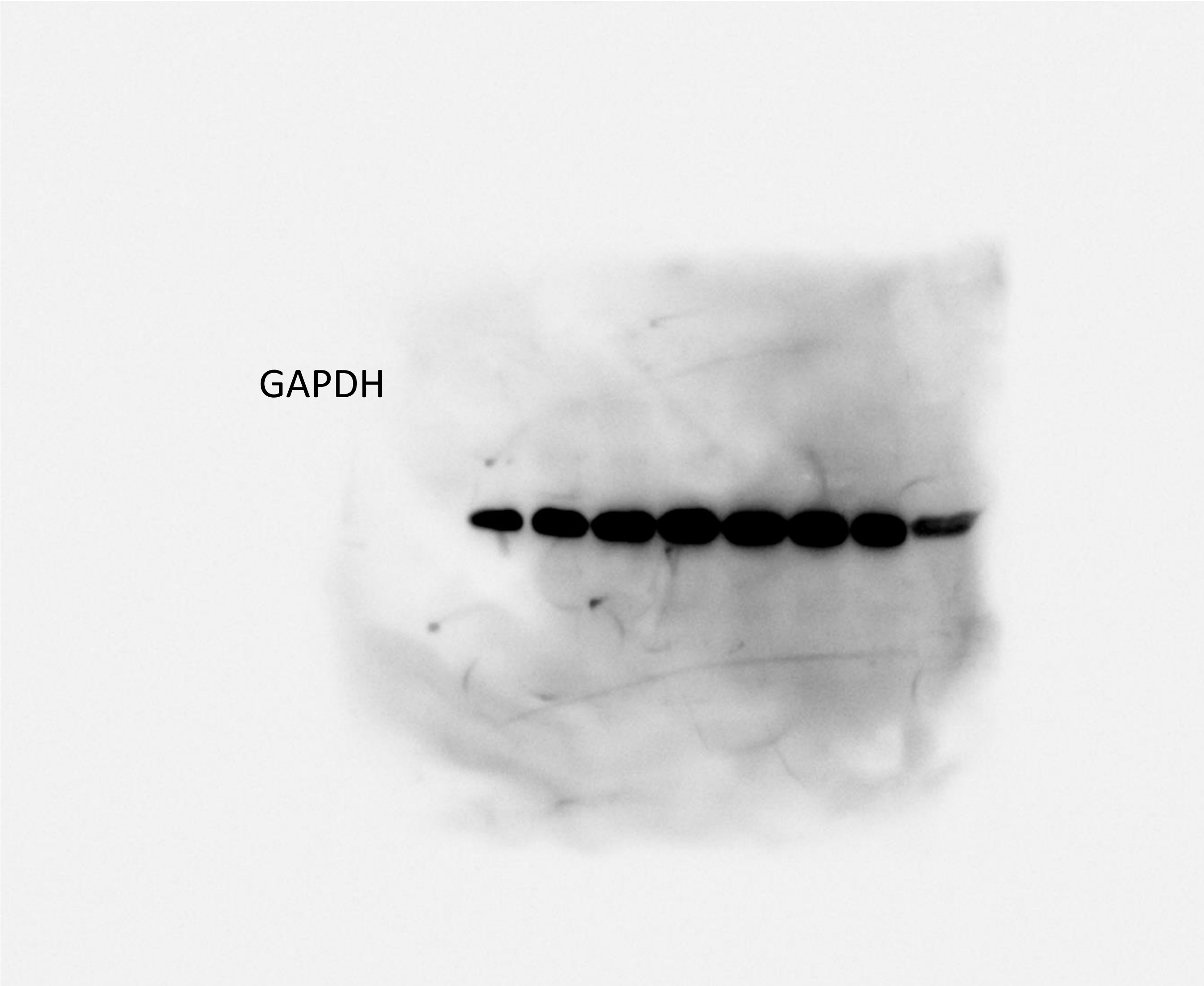

T47D cell line + treatment

Cisplatin, Oxaliplatin

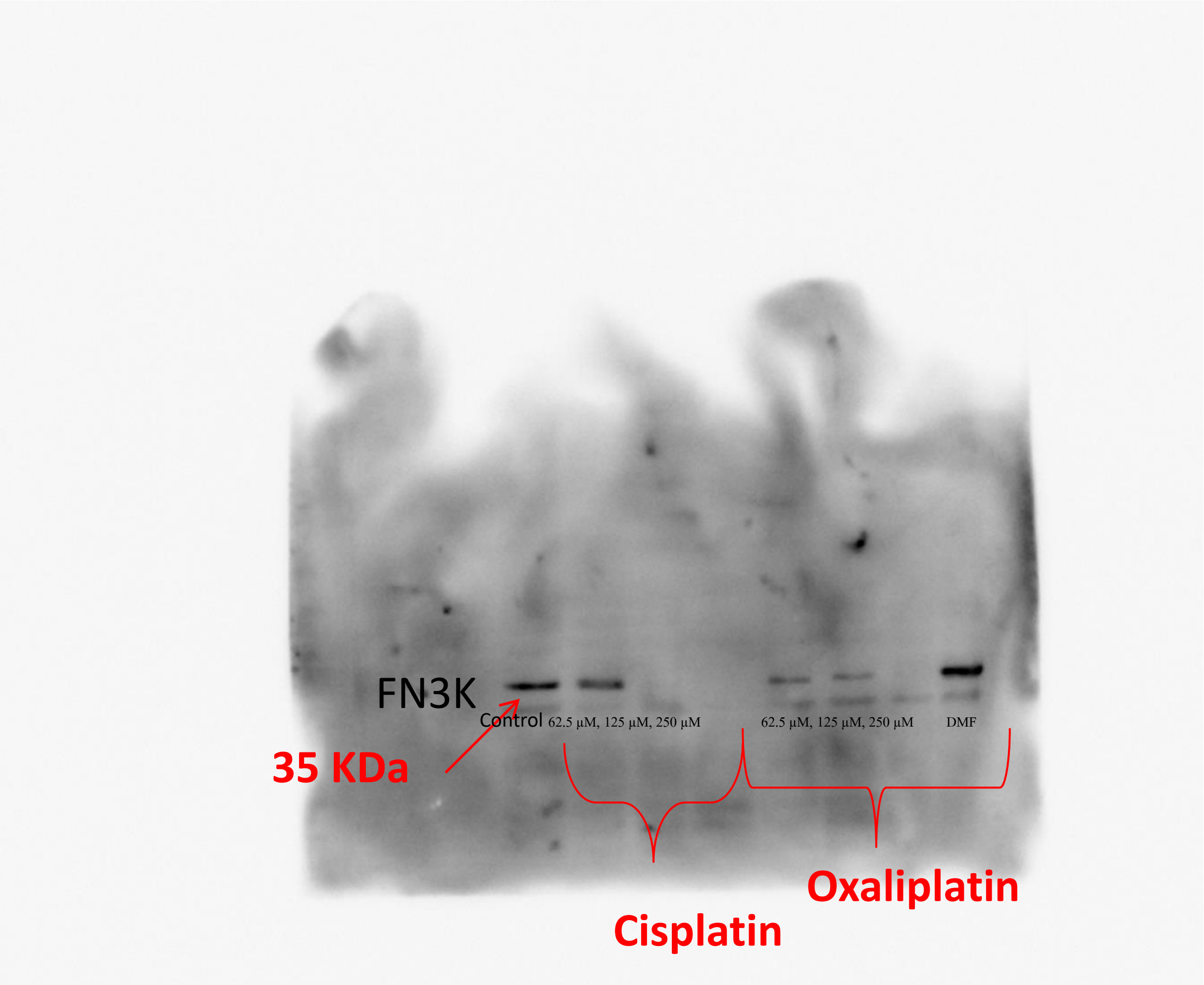
FN3K
Control 62.5 µM, 125 µM, 250 µM
62.5 µM, 125 µM, 250 µM
DMF
35 KDa
Cisplatin
Oxaliplatin

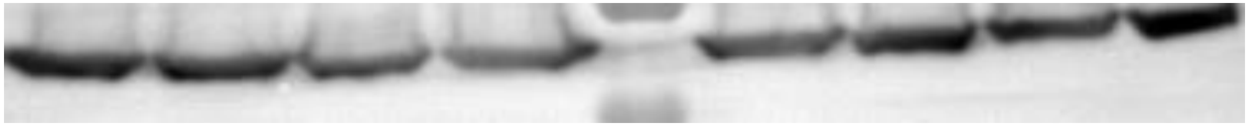

GAPDH

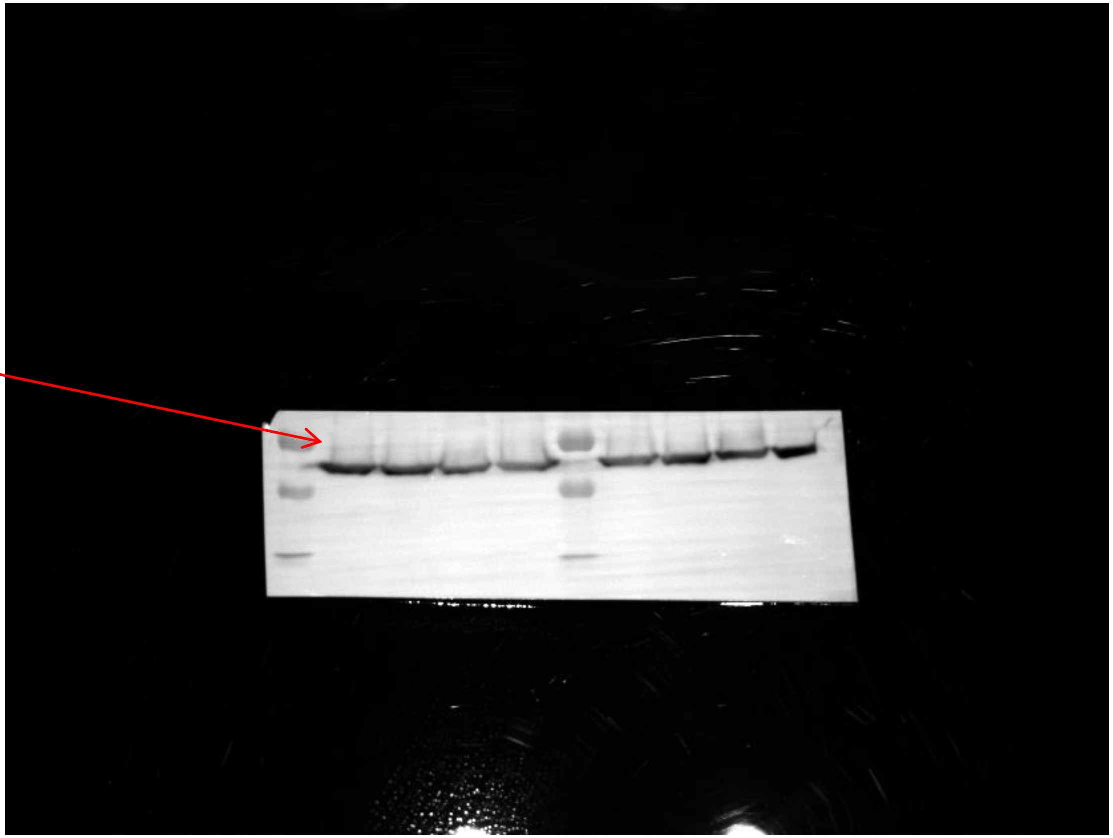

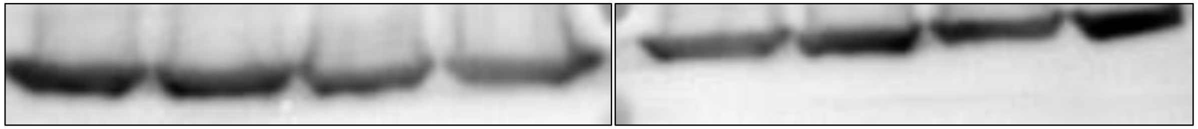

Gefitinib, Neratinib + T47D

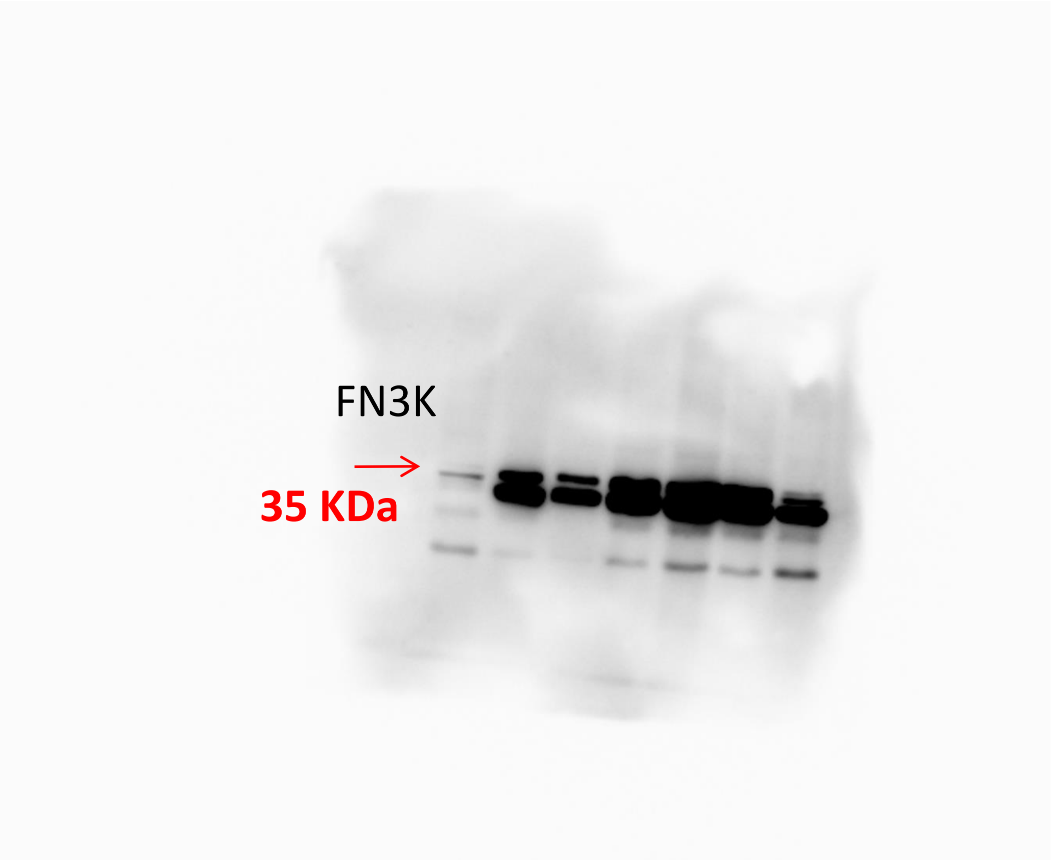
FN3K
35 KDa

GAPDH

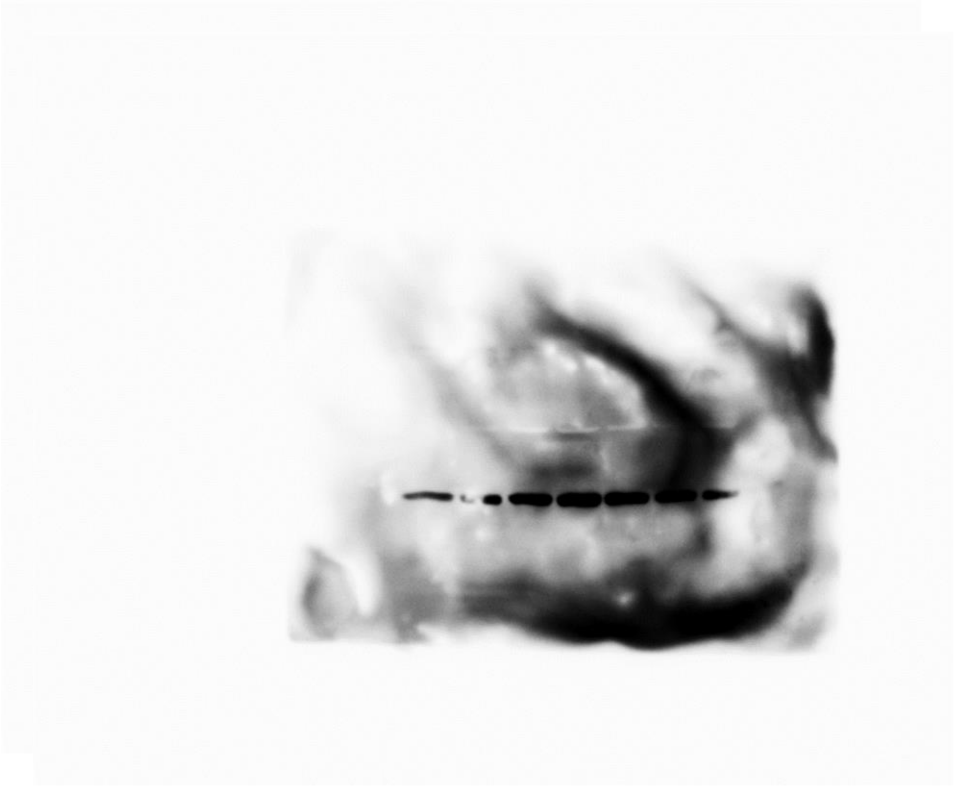

Sorafenib, Tamoxifen Citrate + T47D

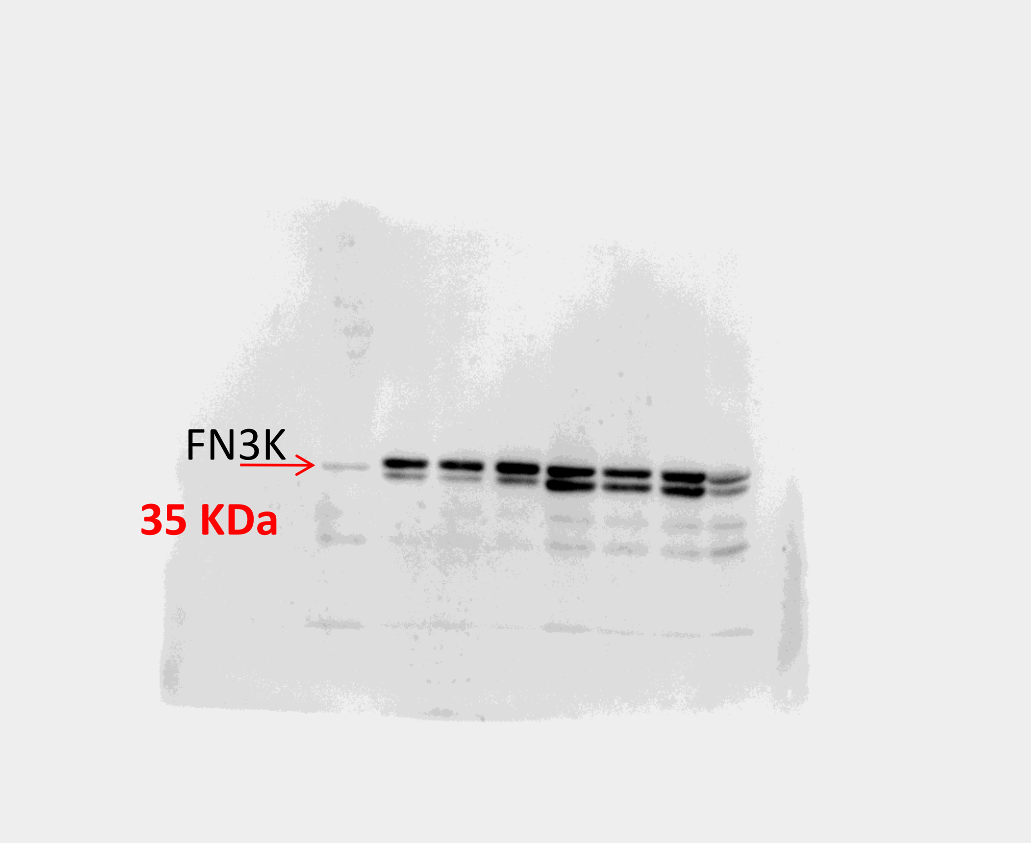
FN3K
35 KDa

GAPDH

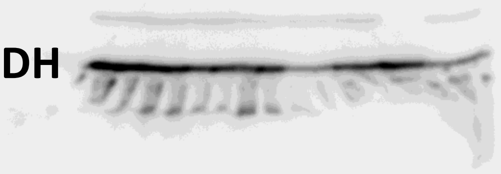

Topotecan + BT-474 + T47D

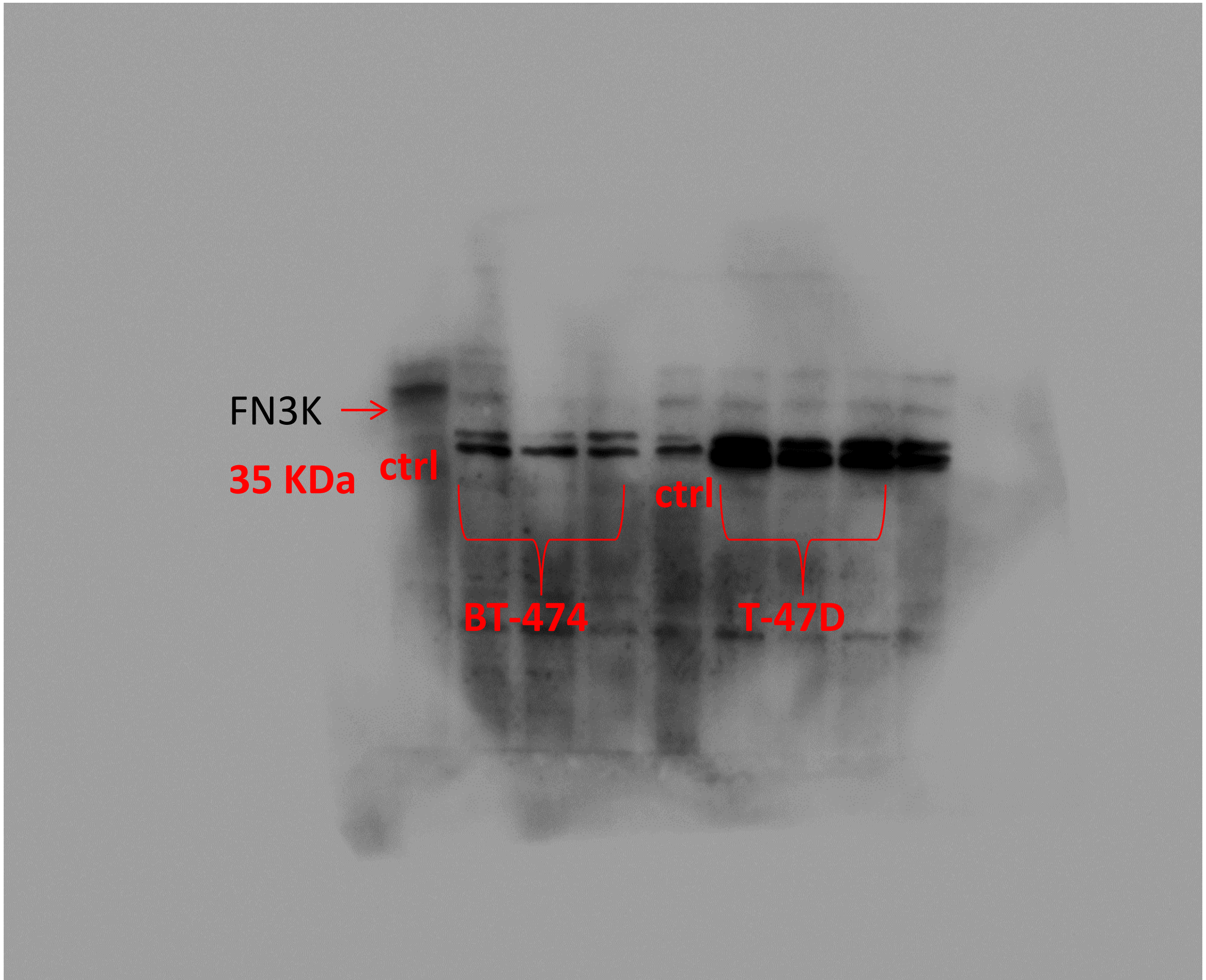
FN3K
35 KDa
ctrl
ctrl
BT-474
T-47D

GAPDH

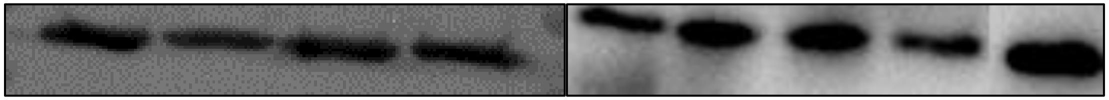

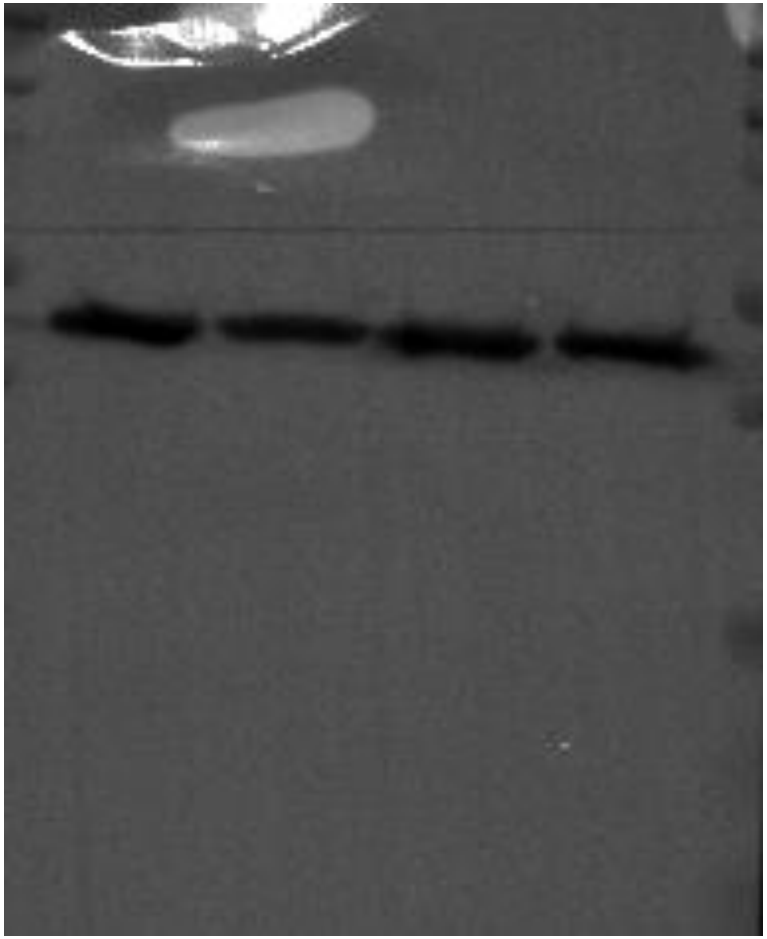

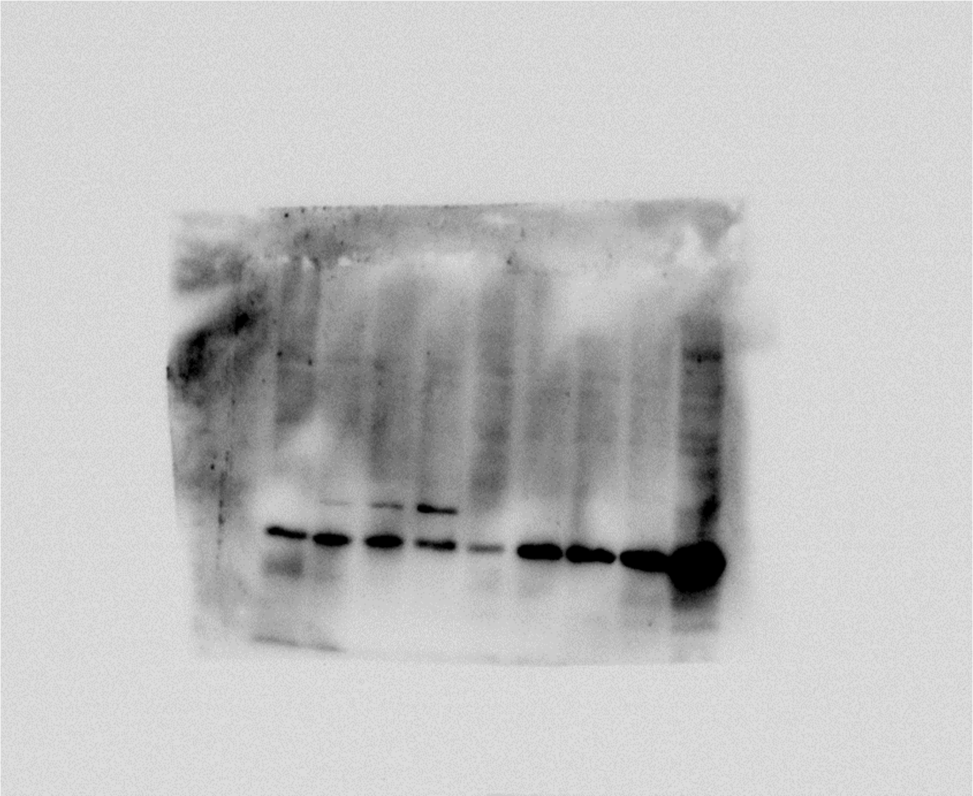

Cyclosprorine+T47D; Brusatol + T47D; Brusatol + BT-474

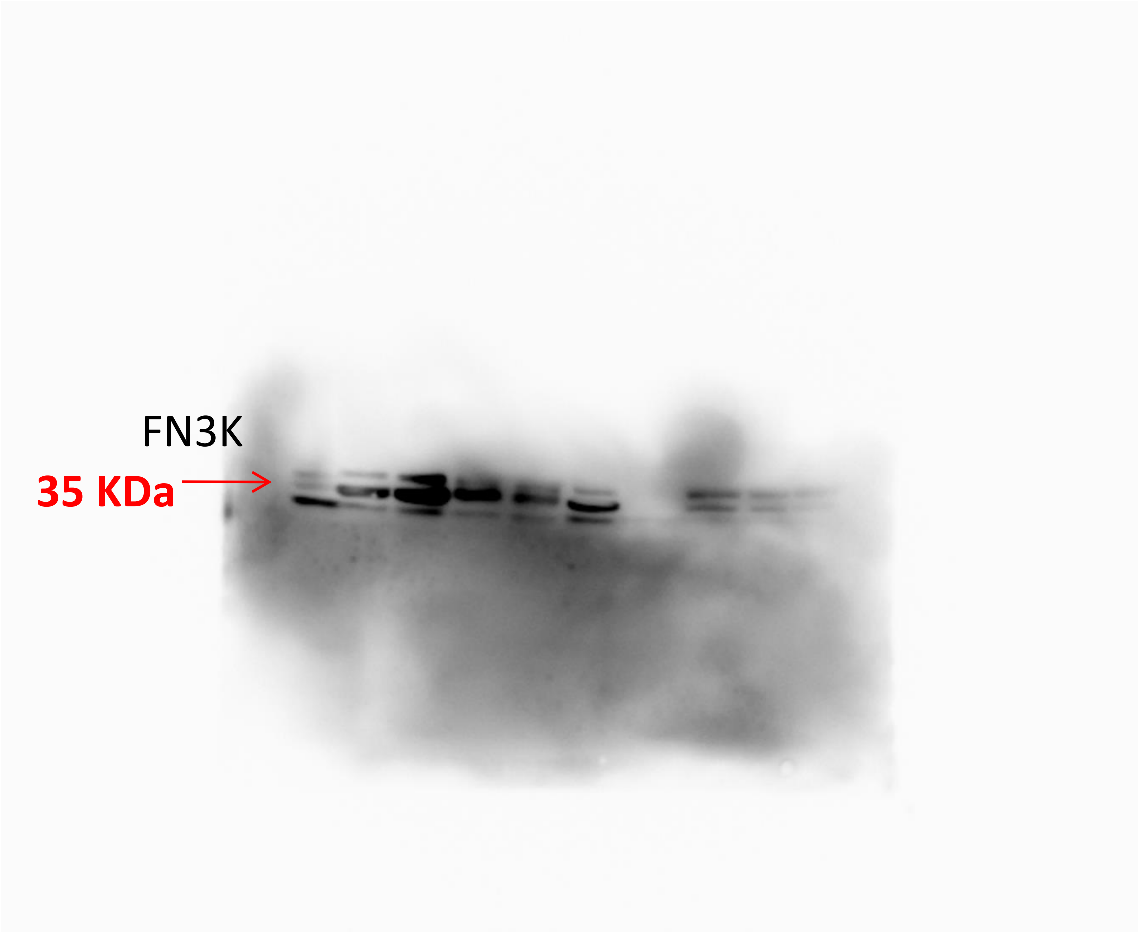
FN3K
35 KDa

GAPDH

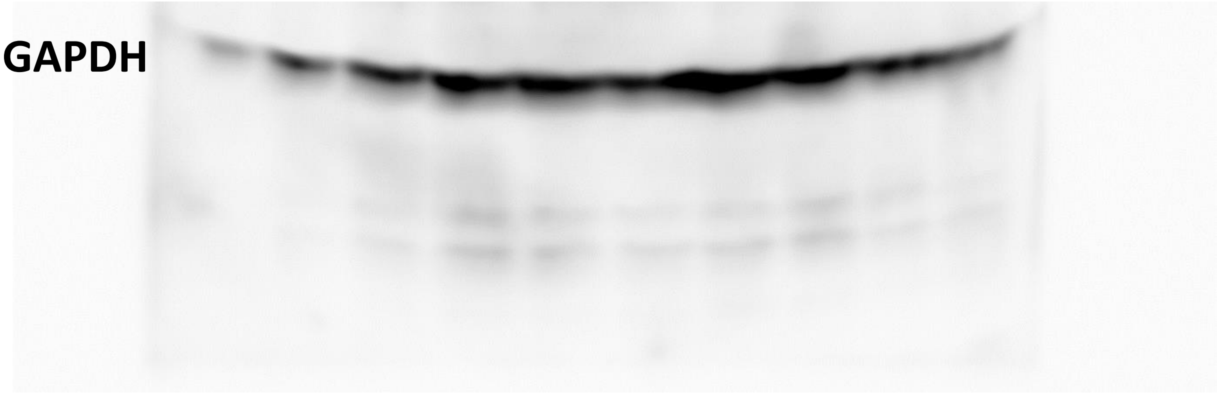

Supplement: S2 File — (PDF) [file pone.0283705.s007.pdf]
